# Radical Coupling Reactions of Hydroxystilbene Glucosides and Coniferyl Alcohol: A Density Functional Theory Study

**DOI:** 10.3389/fpls.2021.642848

**Published:** 2021-03-02

**Authors:** Thomas Elder, Jorge Rencoret, José C. del Río, Hoon Kim, John Ralph

**Affiliations:** ^1^USDA-Forest Service, Southern Research Station, Auburn, AL, United States; ^2^Instituto de Recursos Naturales y Agrobiología de Sevilla, CSIC, Seville, Spain; ^3^Department of Energy Great Lakes Bioenergy Research Center, Wisconsin Energy Institute, University of Wisconsin, Madison, WI, United States; ^4^Department of Biochemistry, University of Wisconsin, Madison, WI, United States

**Keywords:** lignin, hydroxystilbene glucosides, density functional theory, quinone methides, rearomatization

## Abstract

The monolignols, *p*-coumaryl, coniferyl, and sinapyl alcohol, arise from the general phenylpropanoid biosynthetic pathway. Increasingly, however, authentic lignin monomers derived from outside this process are being identified and found to be fully incorporated into the lignin polymer. Among them, hydroxystilbene glucosides, which are produced through a hybrid process that combines the phenylpropanoid and acetate/malonate pathways, have been experimentally detected in the bark lignin of Norway spruce (*Picea abies*). Several interunit linkages have been identified and proposed to occur through homo-coupling of the hydroxystilbene glucosides and their cross-coupling with coniferyl alcohol. In the current work, the thermodynamics of these coupling modes and subsequent rearomatization reactions have been evaluated by the application of density functional theory (DFT) calculations. The objective of this paper is to determine favorable coupling and cross-coupling modes to help explain the experimental observations and attempt to predict other favorable pathways that might be further elucidated via *in vitro* polymerization aided by synthetic models and detailed structural studies.

## Introduction

Lignin is canonically formed by the oxidation and polymerization of *p*-coumaryl, coniferyl, and sinapyl alcohols derived from the general phenylpropanoid biosynthetic pathway (Ralph et al., 2019; Vanholme et al., 2019). Increasingly, however, it is being found that other phenolic compounds, formed either naturally or through genetic modifications, can behave as true lignin monomers and are incorporated into the polymer during lignification of plant cell wall. As of a 2019 review, 35 phenolic monomers had been found in natural lignins (Vanholme et al., 2019). These monomers, like the cinnamyl alcohols, arise through the general phenylpropanoid pathway (del Río et al., 2020), and include monolignol acetates (del Río et al., 2007), benzoates (Kim et al., 2020), *p-*hydroxybenzoates (Lu et al., 2015), *p-*coumarates (del Río et al., 2008) and ferulates (Karlen et al., 2016), caffeyl alcohol (Chen et al., 2012), 5-hydroxyconiferyl alcohol (Ralph et al., 2001), hydroxycinnamaldehydes, and dihydroconiferyl alcohol (Ralph et al., 1997). In addition to these, there have been recent reports of phenolic compounds that are fully incorporated into the lignin polymer, produced through hybrid processes that combine the phenylpropanoid and acetate/malonate ketide or the amino acid pathway (del Río et al., 2020). To date, the lignin monomers that have been identified include flavonoids and hydroxystilbenes, that are produced by the former biosynthetic pathway, and hydroxycinnamic amides that are formed by the latter pathway.

The flavone tricin was the first lignin monomer discovered that comes from one of these hybrid metabolic pathways. Tricin was initially identified in wheat straw lignin, but appeared in the lignin of all commelinid monocotyledons examined (del Río et al., 2012; Lan et al., 2015, 2016b). Tricin and monolignols were found to cross-couple *in vitro* via 4′−*O*−β coupling, and tricin was found in a high molecular weight fraction of isolated maize lignin (Lan et al., 2016a). Subsequently, tricin has been reported to be widely distributed in the lignins of monocotyledons and the amounts of tricin incorporated into the lignin are greater than the readily extractable levels, possibly leading to a new source for a valuable chemical (Lan et al., 2016b). The isolation of metabolites from lignifying tissue in maize revealed cross-coupling between tricin, monolignols and the γ-acylated derivatives thereof (Lan et al., 2016a). Although such structures are known to occur as flavonolignans, it was found that these isolates were racemic rather than optically active, providing additional evidence for tricin-lignin cross-coupling, leading to the term “tricin-oligolignols” and flavonolignins.

Among the hydroxycinnamic amides, feruloyltyramine was identified in several plants of the Solanaceae, including tobacco and potato tubers, and were reported to couple with lignin models through 8−*O*−4′ and 8−5′ linkages (Negrel et al., 1996; Ralph et al., 1998; Kudanga et al., 2009; del Río et al., 2020). Likewise, the presence of diferuloylputrescine was detected in the lignin of maize grain fibers (del Río et al., 2018). Diferuloylputrescine can be oxidized by peroxidases, and homo-coupling and cross-coupling reactions with ferulates and monolignols are proposed to occur through the 4−*O*-, 5- and 8-positions. Indeed, an 8−5′ phenylcoumaran linkage has been identified, with a preliminary assignment of an 8−*O*−4′ bond.

The hydroxystilbenes piceatannol and, to a lesser extent, resveratrol and isorhapontigenin, have been found in lignins isolated from palm fruit endocarps (del Río et al., 2017). Piceatannol was released from the isolated lignins by DFRC (derivatization followed by reductive cleavage) (Lu and Ralph, 1997), indicating its incorporation into the polymer. Piceatannol can homo-couple and cross-couple with monolignols, through 8−*O*−4′ and β−*O*−4′ linkages, forming benzodioxane rings after the post-coupling rearomatization reactions involving internal trapping of the quinone methide intermediate. Homo-coupling can occur throughout the extended conjugated system, including producing 8−10′-coupled units resulting in a phenylcoumaran ring. Additionally, β−8′ cross-coupling has been proposed as a feasible reaction, but to date this linkage has not been identified in the lignin.

**FIGURE 1:**
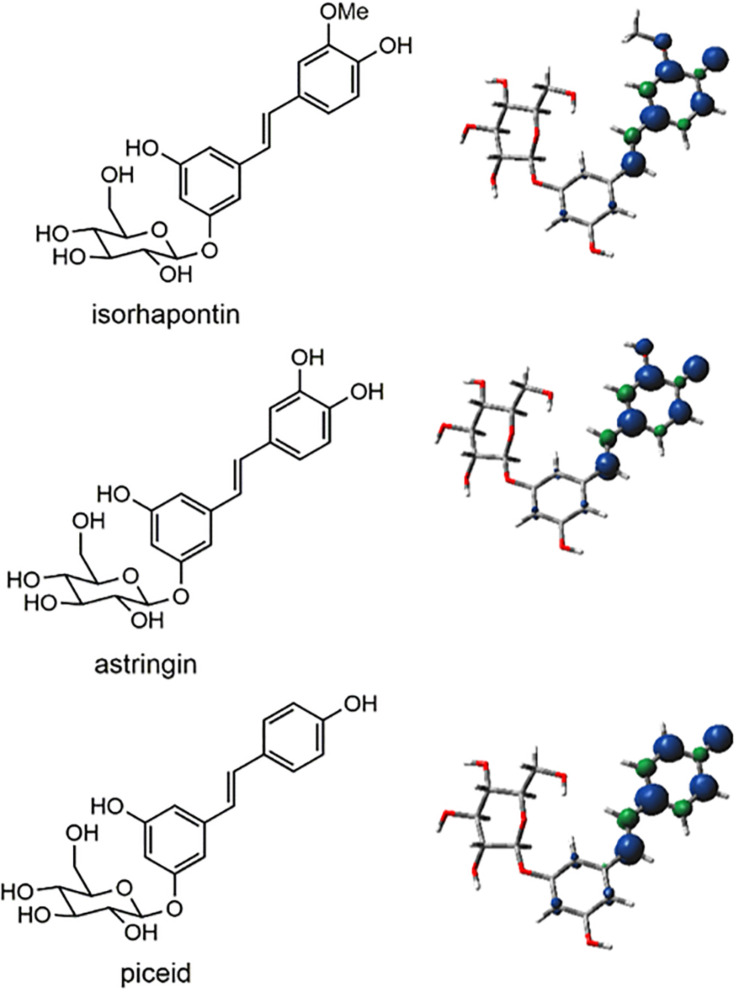
Structures of hydroxystilbene glucosides and spin density plots, in which the blue spheres represent regions of unpaired electron density.

The subject of the current paper, hydroxystilbene glucosides, have been recently reported to occur in the bark lignin of Norway spruce (Rencoret et al., 2019). The hydroxystilbene glucosides, astringin (piceatannol-*O*-glucoside), piceid (resveratrol-*O*-glucoside), and isorhapontin (isorhapontigenin-*O*-glucoside), are substrates for peroxidases, resulting in phenoxy radicals in which there can be considerable delocalization of the unpaired electron as in their respective aglycones (Elder et al., 2019), and as shown in Figure 1, leading to numerous possible interunit linkages arising from homo- and cross-coupling of hydroxystilbene glucosides and monolignols. Results from nuclear magnetic resonance (NMR) spectroscopy on lignin fractions isolated from Norway spruce bark revealed the presence of G-lignin, small amounts of H-lignin, and hydroxystilbenes. In addition, signals for glucose were observed, verifying the presence of hydroxystilbene glucosides. Furthermore, the glucosylation was shown to occur through a phenolic position on the resorcinol ring of the hydroxystilbenes, i.e., on phenols that are not involved in extended conjugation and are therefore not involved in traditional radical coupling. NMR has shown coupling of hydroxystilbene glucosides to produce benzodioxane and phenylcoumaran units. With respect to the latter, a phenylcoumaran structure arising from 8−10′ coupling was conclusively identified in the lignin from Norway spruce bark. Although other possible phenylcoumaran structures arising from other linkages (i.e., 8−5′ and 8−12′) could occur, their identification is difficult due to the similarities in NMR signals. Cross-coupling of astringin and coniferyl alcohol to produce benzodioxane units within the lignin polymer has also been reported. Additional evidence for the incorporation of hydroxystilbene glucosides into the lignin of Norway spruce bark was provided by Diffusion-ordered Spectroscopy, that confirmed that they were part of the polymer.

Based on these observations, the objective of the current work is concerned with evaluating and comparing the thermodynamics of the proposed coupling reactions to determine if there are differences between coupling modes of the hydroxystilbene glucoside reactants. This will be accomplished by the application of contemporary methods in computational chemistry. Such methods have been applied to the general chemistry of lignin (Sangha et al., 2012, 2014; Younker et al., 2012; Berstis et al., 2016; Elder et al., 2016; Gani et al., 2019) and more specifically to novel lignin monomers (Elder et al., 2019, 2020).

## Materials and Methods

**FIGURE 2:**
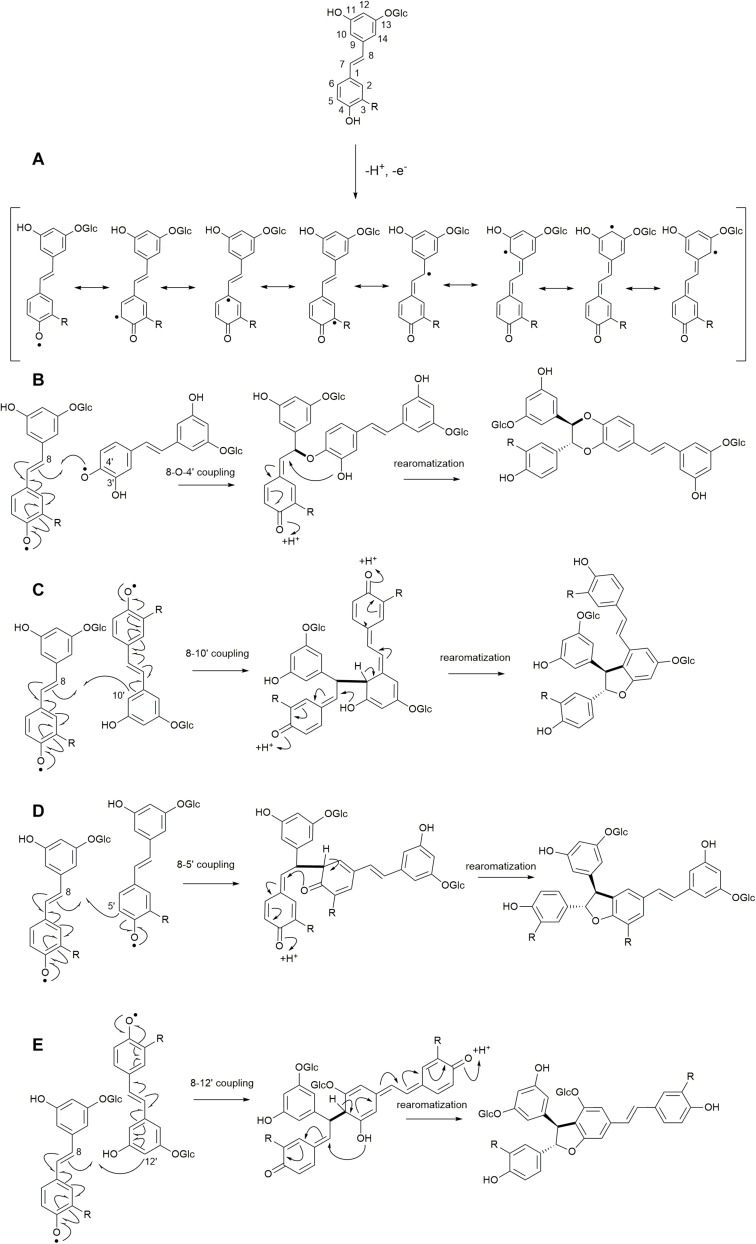
**(A)** Dehydrogenation reactions of hydroxystilbene glucosides and resonance forms of the radicals, **(B)** 8−*O*−4′ homo-coupling of astringin (R=OH), and cross-coupling of astringin and isorhapontin (R=OMe), **(C)** 8−10′ homo-coupling of astringin (R=OH) and isorhapontin (R=OMe), **(D)** 8−5′ homo-coupling of astringin (R=OH) or isorhapontin (R=OMe), **(E)** 8−12′ homo-coupling of astringin (R=OH) or isorhapontin (R=OMe). (Adapted from Rencoret et al., 2019).

**FIGURE 3:**
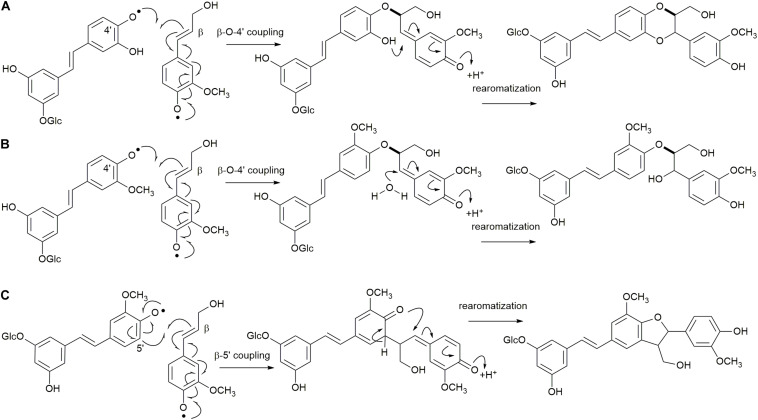
**(A)** 4′−*O*−β Cross-coupling of coniferyl alcohol and astringin, **(B)** 4′−*O*−β cross-coupling of coniferyl alcohol and isorhapontin, **(C)** 5′-β cross-coupling of coniferyl alcohol and isorhapontin. (Adapted from Rencoret et al., 2019).

The reactions to be assessed are as shown in Figures 2, 3. Figure 2A illustrates the initial dehydrogenation step, essential for any inclusion into the lignin polymer. Calculations were carried out on astringin, piceid, and isorhapontin. Figures 2B–E represent coupling reactions between the hydroxystilbene glucosides through different linkages. Figure 2B shows quinone methide formation resulting from 8−*O*−4′ coupling between two astringin free radicals or an astringin and an isorhapontin radical, both of which rearomatize to the benzodioxane products. The reactions depicted in Figures 2C–E are combinations between coupling of an astringin (or isorhapontin) radical and another astringin (or isorhapontin) radical, as discussed in Rencoret et al. (2019). The cross-coupling reactions depicted in Figure 3 all involve the coniferyl alcohol radical cross-coupling with an astringin (Figure 3A) or an isorhapontin radical (Figures 3B,C).

Given the size and numerous rotational degrees of freedom within these structures, the first step in the analysis was a conformational search using a 1000 step Monte Carlo procedure and MMFF (Merck Force Field) minimization. The lowest 100 energy conformers, within a 40 kJ mol^–1^ window were subsequently optimized at the HF/3−21^∗^ level of theory. These calculations were performed as implemented in Spartan’18 (“Spartan’18, 2019”). An automated version of this approach has been described (Hehre et al., 2019) in which accurate Boltzmann weighting of conformationally flexible molecules was reported. The 10 lowest energy conformations were next refined using the M06−2X density functional theory (DFT) method and the 6−31+G(d) basis set, with the GD3 empirical dispersion correction. The lowest energy conformation from this step was further optimized with M06−2X/6−311++G(d,p), the GD3 correction, and frequency calculation to verify the identification of a stationary point and to extract thermal corrections for enthalpy and Gibbs free energy at 298.15 K. All DFT calculations were performed using Gaussian 16 (Gaussian 16 et al., 2019). The default optimization criteria were used throughout. All calculations were done in gas phase. As all reactants and products are uncharged, the solvent effect on the energetics is expected to be minor.

## Results and Discussion

The first step in any of these coupling reactions is, of necessity, the initial dehydrogenation step, the results of which are shown in Table 1. For comparison purposes the hydroxystilbenes are included as well. The differences among the hydroxystilbene glucosides are small, with a range of 0.4 kcal mol^–1^ for Gibbs free energy, in the order astringin > isorhapontin > piceid. For comparison purposes the differences among the hydroxystilbenes are slightly greater at 1.5 kcal mol^–1^ for Gibbs free energy, with the same ordering as for the corresponding hydroxystilbene glucosides. These values are not inconsistent with the bond dissociation energy for coniferyl alcohol, such that the dehydrogenation of the hydroxystilbene glucosides would not represent a thermodynamic obstacle to their incorporation into the lignin polymer.

**TABLE 1:** Gibbs free energy for dehydrogenation reactions.

	R_1_	R_2_	Gibbs Free Energy of reaction (kcal mol^–1^)
Piceid	−H	−OGlc	75.8
Astringin	−OH	−OGlc	76.6
Isorhapontin	−OMe	−OGlc	76.0
Resveratrol	−H	−OH	75.0
Piceatannol (Elder et al., 2019)	−OH	−OH	76.4
Isorhapontigenin	−OMe	−OH	76.0
Coniferyl alcohol (Elder et al., 2019)			77.1

### Reactions of the Hydroxystilbene Glucosides

#### Quinone Methide Formation

**FIGURE 4:**
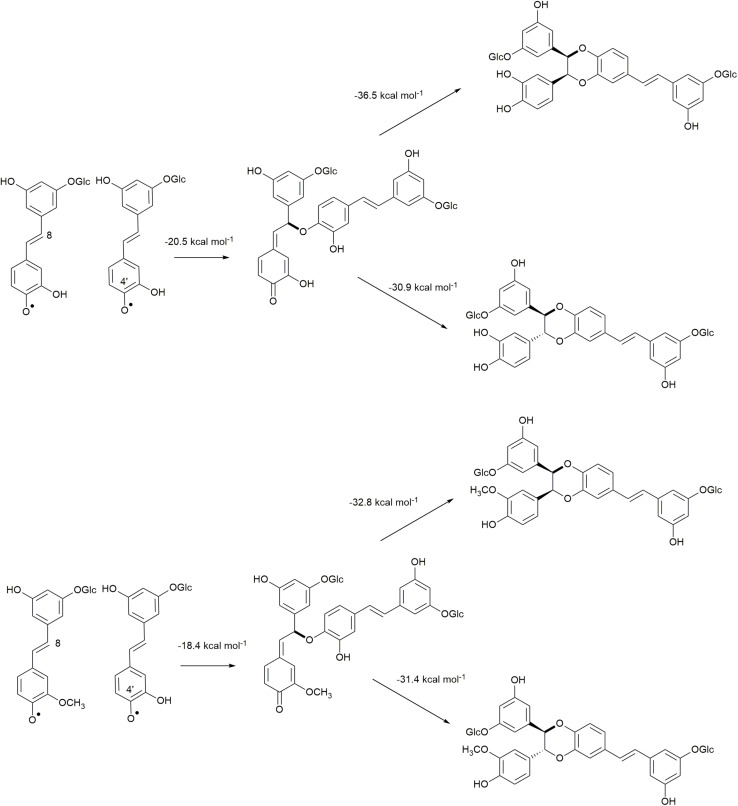
Quinone methide formation of 8–*O*–4′ coupling and rearomatization reactions of homo-coupled astringin and cross-coupled astringin-isorhapontin.

The 8−*O*−4′ coupling reactions between two astringin radicals and by astringin-isorhapontin radicals are shown in Figure 4, in which homo-coupling of astringin is found to be slightly (2.2 kcal mol^–1^) more exergonic than cross-coupling of astringin with isorhapontin. Both of these reactions are, however, less exergonic than the analogous homo-coupling of piceatannol at −24.7 kcal mol^–1^ (Elder et al., 2019).

**FIGURE 5:**
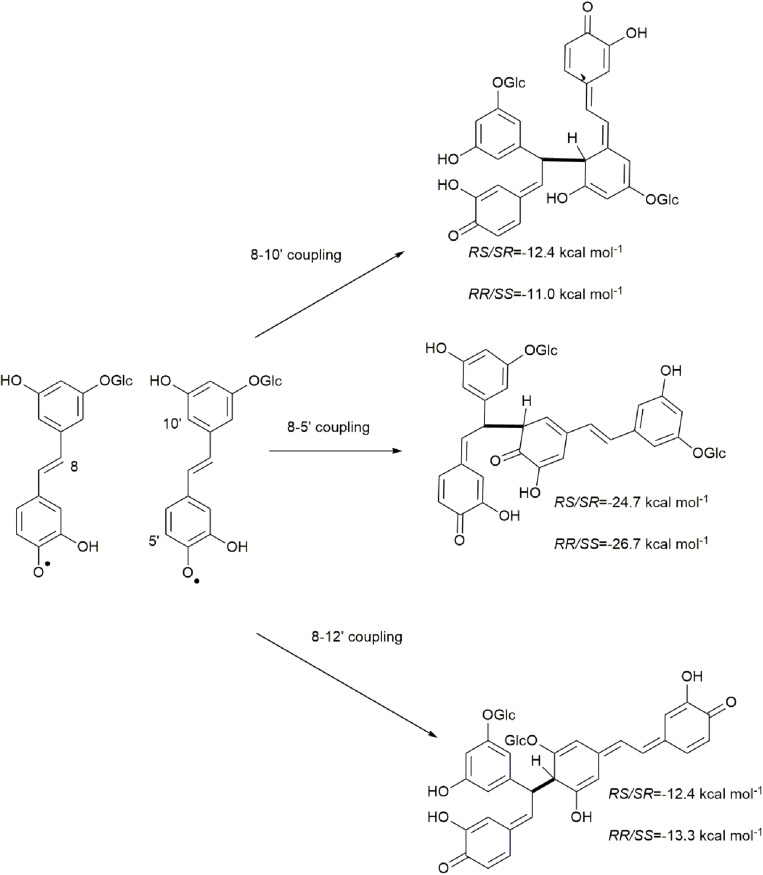
Quinone methide formation of 8−10′, 8−5′, and 8−12′ homo-coupled astringin.

**FIGURE 6:**
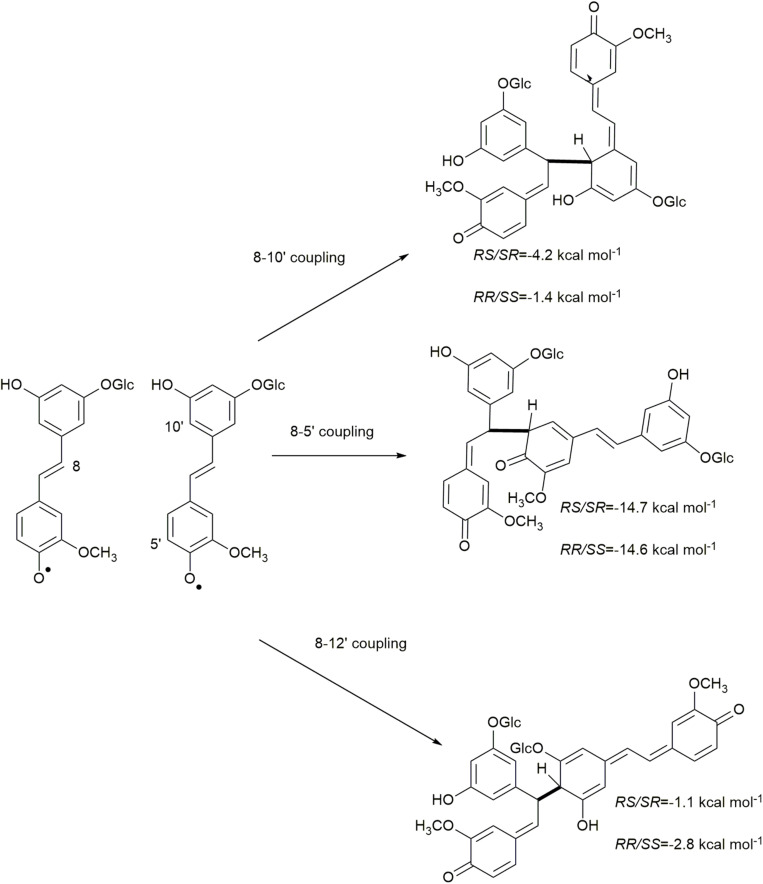
Quinone methide formation of 8−10′, 8−5′, and 8−12′ homo-coupled isorhapontin.

Due to electron delocalization (Figures 1, 2A) the hydroxystilbene glucosides astringin and isorhapontin can also couple through 8−10′, 8−5′, and 8−12′ linkages, as shown in Figures 2B–E. The Gibbs free energies of reaction for these couplings are as shown in Figures 5, 6. The homo-couplings of astringin (Figure 5) are uniformly more exergonic than isorhapontin (Figure 6). For both hydroxystilbene glucosides, the quinone methides formed by 8−10′ and 8−12′ coupling are less exergonic than from the 8−5′ coupling. This is probably due to greater retention of aromaticity in the 8−5′ reaction. It can be seen that the exergonicity of the 8−10′ and 8−12′ isorhapontin quinone methides are quite low, but these results are consistent with the 8−10′ homo-coupling of piceatannol (Elder et al., 2019). It will also be noticed that these reactions generate two chiral centers. There are small differences in energy with stereochemistry. Among the stereoisomers of the astringin quinone methides the *RS/SR* of the 8−10′ is 1.4 kcal mol^–1^ more stable, the *RR/SS* of the 8−5′ is 2.0 kcal mol^–1^ more stable and the *RR/SS* of the 8−12′ is 0.9 kcal mol^–1^ more stable than the other stereoisomers (Figure 5). The stereoisomers of the isorhapontin quinone methides (Figure 6) are slightly more variable with the *RS/SR* of the 8−10′ 2.8 kcal mol^–1^ more stable, the *RS/SR* of the 8−5′ 0.2 kcal mol^–1^ more stable and the *RR/SS* of the 8−12′ 1.7 kcal mol^–1^ more stable than the other stereoisomers (Figure 6). These values do not differ markedly from those found for the 8−10′ quinone methides of piceatannol in which the *RS/SR* stereoisomer is 1.2 kcal mol^–1^ more stable (Elder et al., 2019). Given that the definition of “chemical accuracy” can vary from 1 to 2 kcal mol^–1^ (Lewars, 2011; Foresman and Frisch, 2015) and the thermochemical accuracy of M06−2X can range from 0.37 to 1.32 kcal mol^–1^ across a range of datasets (Zhao and Truhlar, 2008), predicting the reaction preferences for the various stereoisomers based on energy cannot be determined within the accuracy of the method.

#### Rearomatization

The Gibbs free energy of rearomatization for the 8−*O*−4′ coupled quinone methides are shown in Figure 4. Among these, the most exergonic involves the formation of the *RS/SR* (*cis*) stereoisomer of homo-coupled astringin, which is 5.6 kcal mol^–1^ more stable than the *RR/SS* stereoisomer. The rearomatization of homo-coupled isorhapontin has free energies of about −31 kcal mol^–1^ for both stereoisomers that differ by only 1.4 kcal mol^–1^, with the *RS/SR* (*cis*) isomer being the more stable. Both of these values are more exergonic than the analogous reaction of piceatannol, which has a value of ∼20 kcal mol^–1^, in which the *trans* isomer is slightly (0.8 kcal mol^–1^) more stable, in agreement with NMR results (del Río et al., 2017; Elder et al., 2019).

**FIGURE 7:**
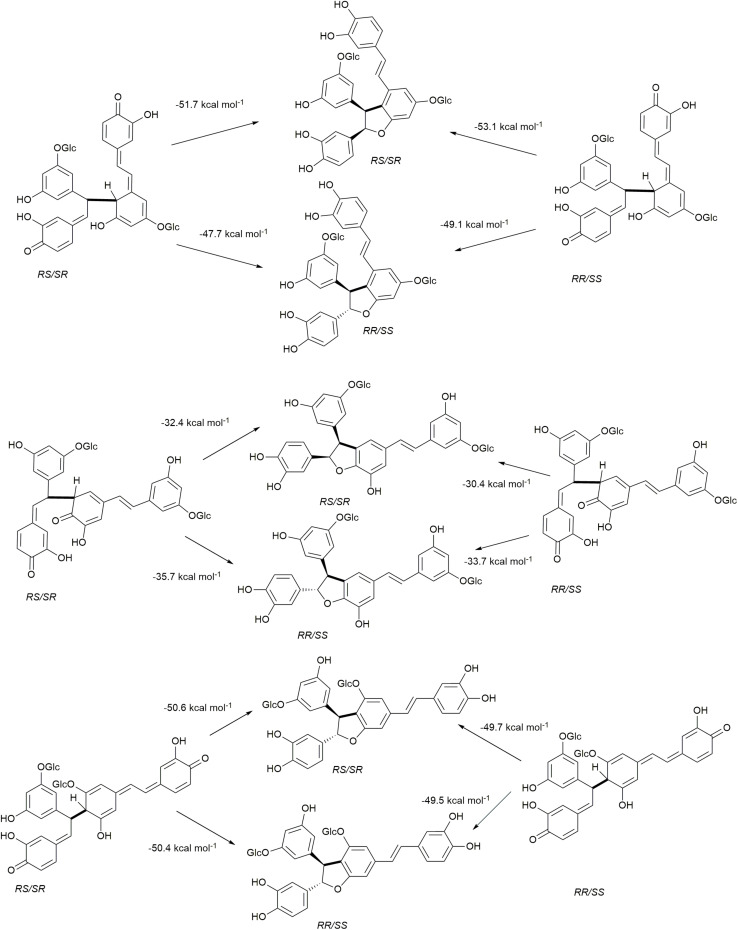
Rearomatization of 8−10′, 8−5′, and 8−12′ homo-coupled astringin quinone methides.

**FIGURE 8:**
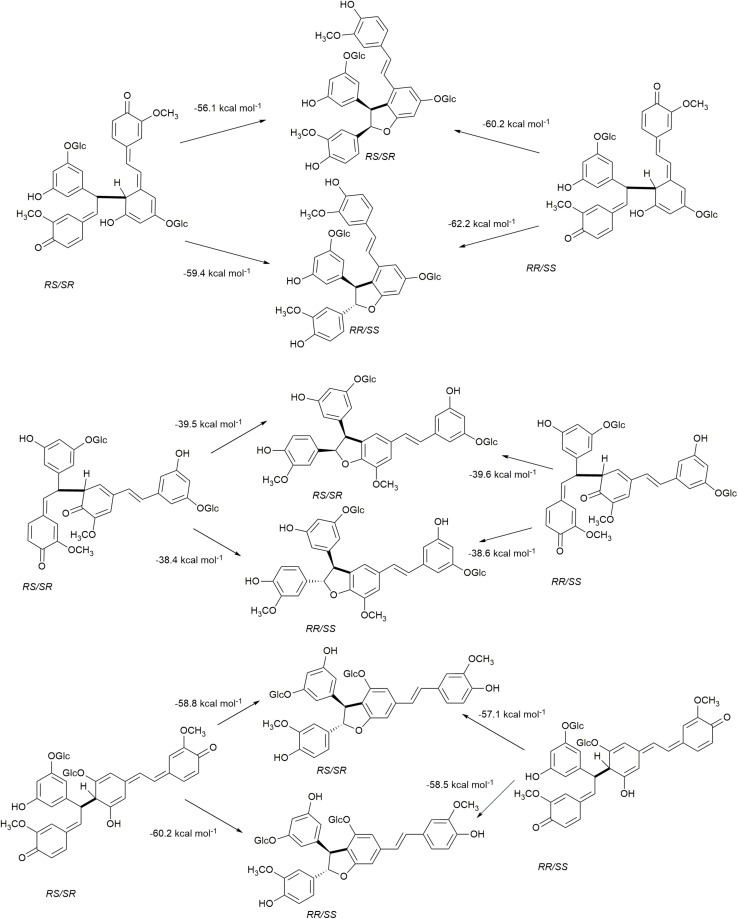
Rearomatization of the 8−10′, 8−5′, and 8−12′ homo-coupled isorhapontin quinone methides.

The rearomatization energies for 8−10′, 8−5′, and 8−12′ homo-coupled astringin quinone methides are shown in Figure 7. As the nucleophilic trapping reaction can occur of either side of C-α, both stereoisomers of the quinone methides can produce both stereoisomers of the products. The energies of rearomatization mirror the stability of the quinone methides, with the less stable 8−10′ and 8−12′ reactants exhibiting markedly higher exergonicity. The effect of stereochemistry on product stability is somewhat variable with the 8−10′ coupling exhibiting the largest (4.0 kcal mol^–1^) and 8−12′ the smallest (0.2 kcal mol^–1^) differences with the *cis* isomer more stable in each case. The stereoisomers of the 8−5′ coupled products differ by 3.4 kcal mol^–1^ with the *trans* phenylcoumaran isomer being the more stable as has been noted previously (Li et al., 1997; Ralph et al., 2009). The rearomatization of the 8−10′, 8−5′, and 8−12′ homo-coupled isorhapontin quinone methides (Figure 8) are uniformly more exergonic than the astringin analogs. The *trans* isomers of the 8−10′ and 8−12′ are the more stable, by 3.3 and 1.4 kcal mol^–1^, respectively, whereas the *cis* isomer of the 8−5′ product is the more stable by 1.1 kcal mol^–1^. For comparison purposes, the rearomatization energy of 8−10′ homo-coupled piceatannol quinone methides ranges from −51.3 to −54.5 kcal mol^–1^ (Elder et al., 2019), which is in general agreement with the current work.

### Cross-Coupling Reactions of Hydroxystilbene Glucosides and Coniferyl Alcohol

#### Quinone Methide Formation

**FIGURE 9:**
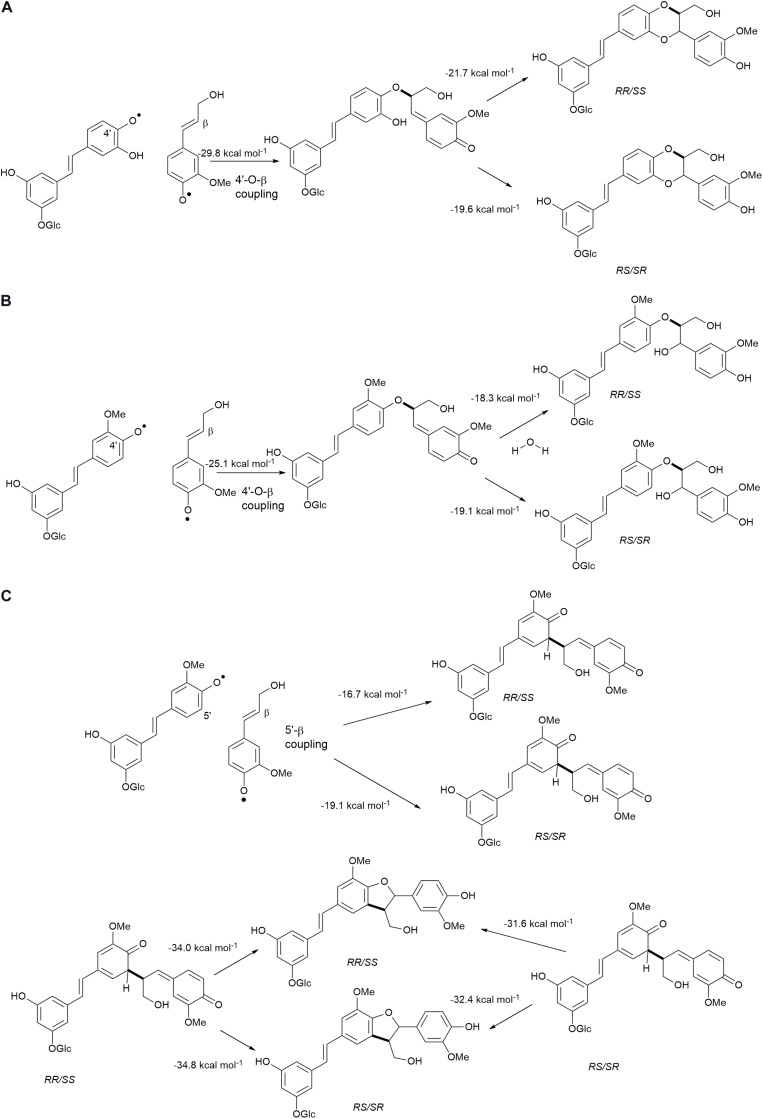
Quinone methide formation and rearomatization reactions of coniferyl alcohol cross-coupled through **(A)** 4′−*O*−β with astringin, **(B)** 4′−*O*−β with isorhapontin, and **(C)** 5′–β with isorhapontin.

The cross-coupling reactions of hydroxystilbene glucoside and coniferyl alcohol radicals to form quinone methides are shown in Figure 9. The 4′−*O*−β couplings (Figures 9A,B) have energies of reaction of −29.8 and −25.1 kcal mol^–1^ for astringin and isorhapontin, respectively. These values are somewhat more exergonic, but compare reasonably well with the cross-coupling reaction of piceatannol and coniferyl alcohol, which has an energy of reaction of −24.5 kcal mol^–1^ (Elder et al., 2019). The 5′–β linked quinone methide made up of isorhapontin and coniferyl alcohol results in the generation of two chiral centers and the finding that the *RS/SR* stereoisomer is more stable by 2.5 kcal mol^–1^. Furthermore, the 5′–β coupling reaction is markedly less exergonic than the 4′−*O*−β couplings, due to the loss of aromaticity in two of the three rings.

#### Rearomatization

The 4′−*O*−β astringin-coniferyl alcohol quinone methide rearomatizes through internal trapping of the *ortho*−OH acting as a nucleophile, resulting in the formation of benzodioxane rings (Figure 9A). The formation of the *RR/SS* stereoisomer, which is the *trans* configuration, is 2.0 kcal mol^–1^ more exergonic. Conversely, due to the presence of the *ortho*-methoxy group in isorhapontin, rearomatization occurs by the nucleophilic addition of water forming an acyclic product (Figure 9B). Among these, the reaction resulting in the *RS/SR* (*erythro*) stereoisomer is slightly (0.8 kcal mol^–1^) more exergonic. The rearomatization energy of the cross-coupled astringin-coniferyl alcohol free radical is somewhat more exergonic (∼20 kcal mol^–1^) than the isorhapontin-coniferyl alcohol quinone methide (∼19 kcal mol^–1^), perhaps due to the ring closure in the former. These are both less exergonic than rearomatization of the cross-coupled piceatannol-coniferyl alcohol quinone methide (∼25 kcal mol^–1^) (Elder et al., 2019).

As was the case with the homo-coupled quinone methides with carbon-carbon bonds, both stereoisomers of the 5′–β linked isorhapontin-coniferyl alcohol quinone methides can form both stereoisomers of the products (Figure 9C). The energies of reaction of the *RS/SR* are somewhat (∼3 kcal mol^–1^) more exergonic due the lower stability of the quinone methide and the similarity in stability of the products. With respect to the latter, the energies of the products only differ by 0.8 kcal mol^–1^, with the *RS/SR* (*trans*) stereoisomer being the more stable.

## Summary

To summarize, in the formation of 8−*O*−4′ couplings, the hydroxystilbene glucoside quinone methides were less exergonic than the corresponding piceatannol reaction, but the rearomatizations were more exergonic, such that overall, the energies of reaction are not dissimilar. Among the carbon-carbon linkages (8−10′, 8−5′, and 8−12′), the homo-coupled astringin quinone methides have energies of reaction that are substantially more exergonic than the homo-coupled isorhapontin quinone methides, whereas the rearomatizations are fairly similar in this regard. In addition, due to decreased aromaticity, the 8−10′ and 8−12′ quinone methides are markedly less stable than the 8−5′ linkage. The results for 8−10′ homo-coupled isorhapontin are consistent with the analogous reactions of homo-coupled piceatannol. Although somewhat variable, there do not appear to be any reactions of the hydroxystilbene glucosides that would be thermodynamically precluded, such that the couplings under consideration should occur readily.

Cross-coupling of astringin and coniferyl alcohol through a 4′−*O*−β linkage is somewhat more exergonic than the isorhapontin-coniferyl alcohol cross-coupling, due to the formation of a benzodioxane ring in the former. The energies of reaction of astringin and coniferyl alcohol via the 4′−*O*−β linkage are quite similar to cross-coupling of piceatannol and coniferyl alcohol, which also results in a benzodioxane ring. The reaction resulting in 5′−β cross-coupled of isorhapontin and coniferyl alcohol quinone methides is less exergonic than the 4′−*O*−β reactions, due to the increased disruption of aromaticity, while the rearomatization is more exergonic because of the ring closures.

As of this writing, the cross-coupling of astringin with coniferyl alcohol to form a benzodioxane unit has been detected experimentally (Rencoret et al., 2019). The other proposed cross-coupled products of the hydroxystilbene glucosides with coniferyl alcohol in the lignin of Norway spruce bark have not been definitively identified yet, but the current results show that there should be no thermodynamic impediments to the reactions and formation of such structures.

## Conclusion

Hydroxystilbene glucosides have been identified in the lignin of Norway spruce and, based on NMR experiments, homo-coupling, and cross-coupling with coniferyl alcohol are observed. Furthermore, their incorporation into the lignin polymer has been confirmed by Diffusion-ordered spectroscopy. Among the possible coupling modes the 8–10′ linkage has been definitively identified. Other possible linkages forming phenylcoumarans have been proposed but not confirmed due to similarities with other NMR signals. Evidence for cross-coupling with coniferyl alcohol, producing benzodioxane units has also been clearly shown.

Based on these observations, the current work has applied contemporary methods in computational chemistry to an assessment of the energetics of radical coupling and rearomatization reactions for homo- and cross-coupling. In addition to the linkages that have been experimentally verified, additional probable combinations were considered. Among these results it is interesting to note that the radical coupling reaction to form the 8–10′ linkage, that has been spectroscopically identified, is less exergonic than other products that have not yet been confirmed. It has also been found that the energetics of the latter linkages are similar to those for other lignin monomers and monolignols. It can therefore be concluded that there should be no thermodynamic impediment to these coupling modes and the incorporation of hydroxystilbene glucosides into the lignin polymer.

## Data Availability Statement

The original contributions presented in the study are included in the article/Supplementary Material, further inquiries can be directed to the corresponding author/s.

## Author Contributions

TE performed the calculations and wrote the preliminary manuscript. JRe, JCR, JRa, and HK performed the experimental isolation and analyses of the compounds. All authors participated in editing and preparation of the final version of the manuscript.

## Conflict of Interest

The authors declare that the research was conducted in the absence of any commercial or financial relationships that could be construed as a potential conflict of interest.
