# Supplementary material for: Radical Coupling Reactions of Hydroxystilbene Glucosides and Coniferyl Alcohol: A Density Functional Theory Study
Source: Front Plant Sci. 2021 Mar 2;12:642848. doi: 10.3389/fpls.2021.642848 (PMC7960926; doi:10.3389/fpls.2021.642848)
Supplement: Supplementary file 1 [file Table_1.DOCX]

Supplementary Material

astringin-astringin-8-O-4 quinone methide

100

Energy: -1821815.8360559

C -0.00750 -3.36646 -0.19957

C 1.54934 -5.07244 -1.08762

C 2.06645 -2.64775 -1.08524

C 2.27614 -3.96097 -1.82840

O 0.67222 -2.39338 -0.95563

C 0.08383 -4.71557 -0.89920

H 2.01159 -5.19185 -0.09617

H 2.52864 -2.72133 -0.08778

H 1.84335 -3.87434 -2.83560

H -0.40290 -4.62278 -1.87836

H 0.42673 -3.43790 0.81041

O -1.34923 -3.01425 -0.14219

O -0.49745 -5.74597 -0.13262

H -1.43653 -5.56064 -0.03160

O 1.71001 -6.25368 -1.84282

H 1.21848 -6.95708 -1.40556

O 3.66114 -4.20613 -1.89767

H 3.77962 -5.10412 -2.22642

C 2.61834 -1.44274 -1.82078

H 3.70369 -1.52332 -1.90271

H 2.19003 -1.41295 -2.82956

O 2.31704 -0.25400 -1.11219

H 1.40527 -0.32837 -0.79773

C -1.69648 -1.85789 0.52437

C -2.58196 0.42894 1.81539

C -0.80969 -1.14511 1.32843

C -3.00478 -1.43178 0.33678

C -3.43660 -0.27264 0.97664

C -1.26672 -0.00109 1.98753

H 0.22791 -1.43312 1.44231

H -3.64292 -1.97577 -0.34966

H -2.89695 1.34670 2.29836

O -0.46821 0.73051 2.78890

H 0.46195 0.46255 2.68611

C -4.75877 0.35776 0.60139

C -5.92858 -0.58422 0.60339

H -5.72027 -1.63660 0.77052

C -7.20671 -0.19428 0.41892

C -9.91131 0.63689 0.01112

C -8.28620 -1.17079 0.43625

C -7.54479 1.21274 0.19219

C -8.81407 1.60575 0.00051

C -9.55844 -0.78841 0.24312

H -8.05169 -2.21544 0.60595

H -6.73728 1.93624 0.16709

H -9.08302 2.64086 -0.17089

O -11.08546 0.92829 -0.15346

O -10.59797 -1.63762 0.24555

H -11.39000 -1.09852 0.08814

C 5.59677 0.92040 0.24233

C 6.54906 -1.31554 0.70701

C 4.30917 -0.61165 1.47514

C 5.51140 -1.48376 1.80644

O 4.72495 0.74263 1.33545

C 6.88622 0.15358 0.50121

H 6.12446 -1.70912 -0.22956

H 3.86558 -0.94953 0.53073

H 5.95177 -1.15835 2.75970

H 7.34614 0.55931 1.41131

H 5.11765 0.56123 -0.68234

O 5.91816 2.26950 0.13682

O 7.77007 0.22149 -0.59528

H 8.01458 1.14204 -0.73400

O 7.68205 -2.07066 1.07767

H 8.35509 -1.95128 0.39921

O 5.06238 -2.81794 1.88908

H 5.83979 -3.38733 1.89541

C 3.23725 -0.60649 2.54233

H 3.65926 -0.24705 3.48747

H 2.84961 -1.61551 2.68534

O 2.15759 0.22206 2.13162

H 2.53493 1.04036 1.77995

C 4.91793 3.13616 -0.24419

C 2.99675 5.01088 -0.97477

C 5.26071 4.48025 -0.28363

C 3.63611 2.71246 -0.57464

C 2.66586 3.65626 -0.92425

C 4.29320 5.40811 -0.65822

H 6.26117 4.80617 -0.03212

H 3.35275 1.66918 -0.55189

H 2.25867 5.74832 -1.27349

O 4.68309 6.71139 -0.69767

H 3.94602 7.26252 -0.97355

C 1.31611 3.13124 -1.16546

H 1.29258 2.06640 -1.37885

C 0.15671 3.78565 -1.03401

H 0.15174 4.83088 -0.73368

C -1.15057 3.11745 -1.12950

C -3.55165 1.68299 -0.94094

C -2.23205 3.62839 -0.40481

C -1.32511 1.92327 -1.83737

C -2.51136 1.21325 -1.73094

C -3.42420 2.92210 -0.30885

H -2.12161 4.56229 0.13995

H -0.52455 1.53750 -2.45659

H -2.64369 0.26063 -2.22955

O -4.66765 0.90916 -0.74689

O -4.48660 3.35280 0.43493

H -4.28234 4.20594 0.82820

H -4.95307 1.19000 1.28425

astringin-astringin 8-O-4-RS

100

Energy: -1821857.7795042

C -1.46494 -1.56308 -1.47282

C 0.96598 -1.80343 -1.82033

C -0.17729 0.39986 -1.65114

C 0.98581 -0.36264 -2.28420

O -1.40539 -0.24183 -1.95416

C -0.38312 -2.43257 -2.11056

H 1.10868 -1.80720 -0.73354

H -0.02598 0.40470 -0.55907

H 0.90592 -0.32536 -3.37912

H -0.55925 -2.47390 -3.19293

H -1.34731 -1.58083 -0.37639

O -2.69232 -2.10727 -1.84345

O -0.37750 -3.72256 -1.53857

H -1.24003 -4.12029 -1.69434

O 2.04513 -2.45875 -2.45227

H 2.25826 -3.25184 -1.94882

O 2.18134 0.26995 -1.85971

H 2.91486 -0.35373 -1.94695

C -0.24243 1.83786 -2.15191

H 0.74575 2.14524 -2.50617

H -0.94676 1.87951 -2.98507

O -0.70592 2.73911 -1.16122

H -0.00525 2.80759 -0.49365

C -3.79547 -1.85000 -1.06621

C -6.07252 -1.62005 0.49110

C -4.91540 -2.61853 -1.37387

C -3.81289 -0.92809 -0.02771

C -4.95256 -0.85435 0.78331

C -6.05983 -2.48181 -0.60382

H -4.86664 -3.31212 -2.20607

H -2.96793 -0.27562 0.13751

H -6.96324 -1.57262 1.10575

O -7.19402 -3.19135 -0.85076

H -7.08357 -3.71938 -1.64580

C -4.98057 -0.07043 2.08175

C -4.32040 1.31698 2.12524

C -4.69606 2.19011 0.94933

C -5.37355 3.77554 -1.24543

C -3.71199 2.60680 0.05684

C -6.01777 2.57226 0.74055

C -6.35500 3.35781 -0.35932

C -4.04352 3.40153 -1.02708

H -2.67827 2.32444 0.20030

H -6.79530 2.26867 1.43382

H -7.37801 3.66412 -0.53944

O -3.13211 3.86900 -1.92323

H -2.23728 3.60091 -1.64655

C 5.53563 0.53961 0.32038

C 6.62273 2.68670 0.85457

C 4.16123 2.43779 0.56376

C 5.34776 3.38574 0.41245

O 4.41847 1.24090 -0.16875

C 6.79318 1.37139 0.11518

H 6.54894 2.47237 1.93131

H 4.01935 2.19580 1.62788

H 5.45200 3.65407 -0.64897

H 6.90579 1.56046 -0.95997

H 5.40321 0.30715 1.38905

O 5.68836 -0.63956 -0.40753

O 7.93552 0.74139 0.64953

H 8.07779 -0.08523 0.17779

O 7.69285 3.57369 0.60995

H 8.51159 3.12986 0.85486

O 5.09572 4.52884 1.19550

H 5.90077 5.05814 1.20373

C 2.87924 3.02998 0.01116

H 3.03708 3.29325 -1.04263

H 2.63666 3.93383 0.56944

O 1.79960 2.12240 0.13827

H 2.01727 1.36444 -0.43710

C 4.85889 -1.69504 -0.11457

C 3.30225 -3.92832 0.38711

C 5.24656 -2.92281 -0.64337

C 3.70688 -1.56131 0.64991

C 2.91838 -2.69007 0.90853

C 4.46274 -4.03708 -0.38193

H 6.14489 -3.00950 -1.23966

H 3.40427 -0.58807 1.00795

H 2.69078 -4.80544 0.57752

O 4.87397 -5.22005 -0.90915

H 4.27495 -5.92367 -0.64452

C 1.64005 -2.59736 1.62793

H 1.11975 -3.53956 1.77577

C 1.03628 -1.45271 1.97299

H 1.52702 -0.50429 1.76277

C -0.33439 -1.31930 2.47340

C -3.06516 -1.01328 2.99575

C -0.96201 -0.08864 2.26908

C -1.08064 -2.37812 3.00948

C -2.43551 -2.22328 3.26546

C -2.31783 0.06023 2.51649

H -0.41560 0.75716 1.86173

H -0.60801 -3.33245 3.20653

H -3.03680 -3.04130 3.64312

O -4.41617 -0.87496 3.13739

O -2.89470 1.23787 2.18006

H -6.02161 0.06751 2.37290

H -4.64993 1.78178 3.06073

O -5.69480 4.55106 -2.31263

H -4.87940 4.74115 -2.79246

astringin-astringin 8-O-4 RR

100

Energy: -1821848.6654813

C -7.00018 -0.00277 -0.29746

C -8.34433 -2.04678 0.11396

C -6.32584 -2.01415 -1.30306

C -7.68774 -2.66793 -1.10653

O -6.47396 -0.60481 -1.46333

C -8.40272 -0.53795 -0.03415

H -7.73158 -2.29027 0.99507

H -5.72540 -2.21531 -0.40978

H -8.31988 -2.48325 -1.98685

H -9.03073 -0.27427 -0.89518

H -6.33589 -0.20328 0.55805

O -7.12805 1.36986 -0.49580

O -8.93772 -0.02399 1.16501

H -8.99898 0.93293 1.08296

O -9.63051 -2.61717 0.23866

H -10.06125 -2.21446 0.99987

O -7.47649 -4.05042 -0.92047

H -8.30320 -4.42647 -0.59823

C -5.57946 -2.52485 -2.51415

H -5.49767 -3.60991 -2.45673

H -6.11959 -2.25217 -3.42682

O -4.25363 -2.00530 -2.54490

H -4.31844 -1.04404 -2.56916

C -6.02288 2.16260 -0.29842

C -3.89880 3.88712 0.15198

C -6.28194 3.48519 0.03841

C -4.72276 1.69091 -0.43538

C -3.66121 2.56479 -0.20030

C -5.21349 4.34139 0.26031

H -7.29931 3.84209 0.12958

H -4.53824 0.66218 -0.71636

H -3.06572 4.56203 0.30993

O -5.50845 5.62716 0.59033

H -4.69543 6.12574 0.70858

C -2.24475 2.05835 -0.32460

C -1.75437 1.30183 0.92945

C -2.61031 0.11044 1.24929

C -4.31645 -2.05078 1.69923

C -2.56022 -1.00561 0.41218

C -3.50511 0.12958 2.31219

C -4.35554 -0.94906 2.54216

C -3.41239 -2.07562 0.62731

H -1.84586 -1.05141 -0.40375

H -3.55188 0.99658 2.96158

H -5.06227 -0.94705 3.36307

O -3.43512 -3.19536 -0.15753

H -3.41389 -2.91449 -1.08893

C 7.88773 -1.41178 -0.23560

C 7.68731 -3.85355 -0.51316

C 5.92760 -2.44715 0.53192

C 6.56581 -3.83122 0.51657

O 6.94264 -1.48536 0.80296

C 8.66993 -2.71553 -0.28129

H 7.24189 -3.72410 -1.51093

H 5.47782 -2.24045 -0.45132

H 6.99896 -4.03230 1.50699

H 9.16967 -2.84689 0.68681

H 7.37956 -1.23693 -1.19774

O 8.77717 -0.37555 0.02602

O 9.59481 -2.74759 -1.34588

H 10.23133 -2.03745 -1.21791

O 8.31340 -5.11596 -0.42416

H 9.03757 -5.13525 -1.05852

O 5.56948 -4.77548 0.20261

H 6.00633 -5.62255 0.06230

C 4.87013 -2.25664 1.60152

H 5.29263 -2.51642 2.57978

H 4.02337 -2.91115 1.39933

O 4.39788 -0.92354 1.59008

H 5.15142 -0.35566 1.78826

C 8.29690 0.91123 -0.07204

C 7.51827 3.56790 -0.24590

C 9.25857 1.91378 0.04893

C 6.96154 1.21645 -0.27945

C 6.56330 2.56061 -0.34889

C 8.85795 3.23909 -0.04594

H 10.29549 1.63968 0.20681

H 6.22244 0.43538 -0.37293

H 7.23889 4.61317 -0.29578

O 9.73937 4.27080 0.05445

H 10.62542 3.93143 0.20529

C 5.14561 2.92561 -0.49223

H 4.95603 3.96014 -0.76458

C 4.12028 2.09733 -0.25551

H 4.32031 1.08305 0.07993

C 2.69185 2.41353 -0.35652

C -0.06027 2.90558 -0.47652

C 1.78287 1.50461 0.18742

C 2.19895 3.57794 -0.96406

C 0.83832 3.81863 -1.02244

C 0.41660 1.74945 0.13927

H 2.13087 0.59730 0.67131

H 2.88047 4.28898 -1.41407

H 0.43900 4.70284 -1.50436

O -1.39726 3.17339 -0.56093

O -0.43177 0.83130 0.68847

H -2.16847 1.36596 -1.17408

H -1.74415 2.01071 1.76726

O -5.17275 -3.08534 1.89032

H -4.99652 -3.75346 1.21295

isorhapontin-astringin 8-O-4 quinone methide

103

Energy: -1846468.3124374

C -0.34314 2.00120 1.37191

C 2.13147 2.35827 1.42360

C 1.07023 0.19664 1.96935

C 2.22148 1.14110 2.32165

O -0.18649 0.85186 2.16933

C 0.77896 3.01527 1.59391

H 2.23278 2.01957 0.38440

H 1.18076 -0.07291 0.91007

H 2.13721 1.45427 3.37292

H 0.68878 3.40067 2.61722

H -0.39876 1.72088 0.30683

O -1.51840 2.66714 1.76028

O 0.68851 4.05703 0.64428

H -0.10665 4.56546 0.82856

O 3.19746 3.22117 1.75732

H 3.28514 3.88371 1.06233

O 3.42645 0.44685 2.10256

H 4.11781 1.09058 1.90498

C 1.08542 -1.07977 2.80451

H 0.40779 -0.94788 3.65403

H 2.09457 -1.24107 3.18366

O 0.76262 -2.24900 2.06581

H -0.14820 -2.23128 1.74339

C -2.68484 2.40352 1.09465

C -5.01774 2.06021 -0.36006

C -3.53093 3.48951 0.89297

C -3.01164 1.13521 0.63176

C -4.16680 0.98602 -0.13567

C -4.70832 3.30673 0.17753

H -3.24756 4.46130 1.28209

H -2.35009 0.30677 0.84747

H -5.93127 1.95061 -0.93432

O -5.58278 4.31778 -0.05923

H -5.28634 5.12228 0.37491

C -4.55399 -0.35093 -0.76222

C -5.68181 -0.96011 0.01283

H -5.39519 -1.30257 1.00453

C -6.97234 -1.06197 -0.36380

C -9.77844 -1.20985 -1.01821

C -7.94071 -1.63150 0.56389

C -7.47122 -0.62370 -1.66650

C -8.77277 -0.70096 -1.97251

C -9.25147 -1.72641 0.27758

H -7.59332 -1.97799 1.53102

H -6.77467 -0.23165 -2.39753

H -9.16199 -0.37437 -2.92957

O -10.96560 -1.19204 -1.27489

O -10.10408 -2.19305 1.21311

C 6.44419 -0.91958 -0.85207

C 7.14466 -3.21976 -0.37478

C 4.83775 -2.40541 0.03851

C 5.90871 -3.39726 0.49401

O 5.39195 -1.10297 0.06282

C 7.62566 -1.77193 -0.42522

H 6.87763 -3.52432 -1.39838

H 4.51447 -2.67250 -0.98147

H 6.16779 -3.17115 1.53883

H 7.93920 -1.44870 0.57576

H 6.13140 -1.21516 -1.86627

O 6.77757 0.43549 -0.82699

O 8.66411 -1.62881 -1.36143

H 9.30698 -2.32227 -1.17654

O 8.23121 -3.99805 0.08451

H 7.92564 -4.90890 0.15550

O 5.50953 -4.74380 0.35246

H 4.61073 -4.82831 0.69977

C 3.63142 -2.34881 0.95570

H 2.95181 -1.57024 0.59545

H 3.96332 -2.04895 1.95566

O 3.00988 -3.62124 0.96664

H 2.15257 -3.50605 1.40176

C 5.77108 1.30818 -1.13786

C 3.76319 3.19128 -1.57324

C 6.00290 2.64682 -0.82134

C 4.56374 0.91539 -1.69805

C 3.53885 1.84812 -1.87306

C 4.99676 3.57616 -1.05033

H 6.95094 2.92753 -0.37661

H 4.38066 -0.11948 -1.93710

H 2.97750 3.93145 -1.67789

O 5.13631 4.89144 -0.71557

H 6.01538 5.04663 -0.35892

C 2.16800 1.40093 -2.17319

H 1.45735 2.17466 -2.45342

C 1.74313 0.15355 -1.92777

H 2.46030 -0.57340 -1.55498

C 0.35241 -0.30674 -1.88640

C -2.28013 -0.98016 -1.25481

C 0.08118 -1.39037 -1.04049

C -0.71523 0.36141 -2.49094

C -2.02872 0.01912 -2.18493

C -1.22027 -1.70303 -0.70268

H 0.88479 -1.94590 -0.56723

H -0.52650 1.17626 -3.17913

H -2.84685 0.58215 -2.61690

O -3.50219 -1.31214 -0.72726

O -1.46275 -2.65063 0.24928

H -2.41970 -2.74261 0.34484

H -4.83687 -0.17680 -1.80333

C -11.13411 -3.09835 0.80174

H -10.76470 -3.77393 0.02644

H -12.00216 -2.55793 0.43207

H -11.38845 -3.67270 1.69064

isorhapontin-astringin 8-O-4 RS

103

Energy: -1846503.9116269

C -2.23946 -2.27001 -1.35075

C -0.09151 -3.34943 -1.95209

C -0.25385 -1.05004 -1.03918

C 0.51556 -1.96085 -1.99603

O -1.61954 -1.00668 -1.43840

C -1.56895 -3.26369 -2.29129

H 0.01591 -3.74686 -0.93094

H -0.16611 -1.45543 -0.02156

H 0.42294 -1.56564 -3.01773

H -1.68869 -2.89472 -3.31807

H -2.18888 -2.64614 -0.31673

O -3.56031 -2.14723 -1.77481

O -2.10560 -4.55963 -2.14778

H -3.03858 -4.53158 -2.38304

O 0.61693 -4.15698 -2.86674

H 0.19223 -5.02088 -2.89442

O 1.86936 -1.97197 -1.59275

H 2.33212 -2.67813 -2.05813

C 0.26376 0.38106 -1.03999

H -0.43384 1.00701 -0.47737

H 0.31445 0.75444 -2.07071

O 1.52218 0.47256 -0.39944

H 2.10847 -0.21629 -0.74934

C -4.44328 -1.44292 -0.98804

C -6.32890 -0.00895 0.44059

C -5.68178 -1.19360 -1.56440

C -4.12839 -0.98653 0.28637

C -5.08215 -0.26022 1.00163

C -6.62146 -0.47112 -0.84231

H -5.90637 -1.54255 -2.56377

H -3.14119 -1.14398 0.68856

H -7.06491 0.57402 0.98587

O -7.81860 -0.24221 -1.44213

H -8.35737 0.32930 -0.88813

C -4.76001 0.37467 2.34069

C -3.74658 1.53877 2.23756

C -4.14775 2.50287 1.15369

C -5.13008 4.08315 -0.92060

C -3.72783 2.26529 -0.15998

C -5.03481 3.53409 1.41992

C -5.52572 4.32807 0.38161

C -4.22182 3.04373 -1.18927

H -3.04076 1.45287 -0.35861

H -5.35321 3.72821 2.43888

H -6.21479 5.14213 0.56884

O -3.91156 2.89388 -2.50769

C 6.04312 0.76749 0.04511

C 7.36177 2.86093 -0.04629

C 4.90077 2.80709 0.26326

C 6.06397 3.60093 -0.32612

O 4.89983 1.49994 -0.31578

C 7.28081 1.43084 -0.54413

H 7.51985 2.84246 1.04233

H 5.03395 2.72645 1.35232

H 5.92937 3.67684 -1.41489

H 7.17116 1.42690 -1.63624

H 6.11751 0.69854 1.14256

O 5.98327 -0.51532 -0.50341

O 8.47011 0.78296 -0.14783

H 8.43954 -0.12429 -0.46723

O 8.40630 3.57763 -0.67296

H 9.22650 3.09478 -0.52727

O 6.08230 4.87995 0.26598

H 6.90853 5.30293 0.00834

C 3.54491 3.43189 0.00125

H 3.34268 3.39252 -1.07723

H 3.58862 4.47802 0.30757

O 2.53327 2.80233 0.74621

H 2.26293 1.98694 0.29840

C 5.13474 -1.42977 0.06746

C 3.46641 -3.40214 1.09153

C 5.40492 -2.76244 -0.21225

C 4.04603 -1.06647 0.85515

C 3.19298 -2.05683 1.35137

C 4.56990 -3.74173 0.31815

H 6.25248 -3.03935 -0.82509

H 3.81069 -0.02449 1.02951

H 2.80673 -4.18317 1.45451

O 4.88217 -5.03436 0.02340

H 4.25938 -5.62445 0.45600

C 1.96251 -1.59598 2.00237

H 1.85695 -0.51555 2.02391

C 0.94801 -2.34743 2.44759

H 1.03853 -3.43043 2.48241

C -0.37136 -1.81225 2.80943

C -3.00463 -0.92817 3.20827

C -0.78039 -0.55398 2.35423

C -1.29760 -2.60057 3.50381

C -2.59724 -2.15862 3.70864

C -2.07881 -0.11508 2.54753

H -0.11747 0.08696 1.78260

H -1.00223 -3.57960 3.86367

H -3.33296 -2.77418 4.21156

O -4.31588 -0.56563 3.31772

O -2.44458 1.04885 1.94272

H -5.67889 0.79238 2.75392

C -3.10413 1.77958 -2.85479

H -2.11049 1.86681 -2.40686

H -3.56599 0.84508 -2.52350

H -3.02062 1.79238 -3.93893

H -3.72275 2.03593 3.21417

O -5.61087 4.83970 -1.93634

H -5.22580 4.51154 -2.75823

isorhapontin-astringin 8-O-4 RR

103

Energy: -1846501.4939208

C 6.94676 0.07143 0.30329

C 8.05277 -2.05982 -0.20071

C 6.11004 -1.84295 1.34812

C 7.34853 -2.66201 1.00689

O 6.46495 -0.47220 1.51176

C 8.28821 -0.56926 -0.01733

H 7.40332 -2.19589 -1.07714

H 5.39473 -1.92871 0.52490

H 8.04243 -2.65092 1.85943

H 8.96925 -0.39103 0.82474

H 6.22851 -0.12428 -0.50768

O 7.12900 1.44334 0.44829

O 8.82903 -0.08798 -1.22793

H 8.96161 0.86141 -1.14313

O 9.26037 -2.77493 -0.37332

H 9.70765 -2.41756 -1.14732

O 6.92116 -3.98159 0.72756

H 7.68443 -4.46169 0.38730

C 5.42055 -2.26576 2.62720

H 6.13042 -2.21965 3.46224

H 5.06491 -3.29118 2.52179

O 4.29465 -1.44440 2.87303

H 4.61122 -0.53481 2.90291

C 6.01882 2.23834 0.28296

C 3.88281 3.95466 -0.15508

C 6.26872 3.56225 -0.05490

C 4.71906 1.76338 0.43544

C 3.65353 2.63147 0.20073

C 5.19501 4.41443 -0.26810

H 7.28387 3.92213 -0.15818

H 4.52886 0.73676 0.72330

H 3.04614 4.62531 -0.31283

O 5.48311 5.70198 -0.59921

H 4.66718 6.19703 -0.71160

C 2.24066 2.11249 0.31621

C 1.75331 1.38440 -0.95810

C 2.60288 0.19250 -1.29757

C 4.32204 -1.95711 -1.73322

C 2.47990 -0.96445 -0.51908

C 3.56214 0.25577 -2.29611

C 4.42520 -0.81902 -2.51380

C 3.32807 -2.03643 -0.73994

H 1.71885 -1.01000 0.24941

H 3.66278 1.15459 -2.89404

H 5.19513 -0.78130 -3.27510

O 3.33658 -3.20341 -0.04242

C -7.87630 -1.37052 0.20762

C -7.66553 -3.81043 0.48804

C -5.90864 -2.39760 -0.55432

C -6.54107 -3.78436 -0.53807

O -6.92591 -1.43948 -0.82744

C -8.65271 -2.67786 0.24991

H -7.22438 -3.67629 1.48715

H -5.45996 -2.18831 0.42897

H -6.97032 -3.98929 -1.52935

H -9.14676 -2.81184 -0.72074

H -7.37236 -1.19471 1.17186

O -8.76935 -0.33962 -0.05741

O -9.58256 -2.71339 1.30958

H -10.22226 -2.00652 1.17767

O -8.28422 -5.07651 0.40002

H -9.01636 -5.09451 1.02532

O -5.54122 -4.72419 -0.21981

H -5.97611 -5.57197 -0.07728

C -4.85203 -2.20570 -1.62469

H -5.27341 -2.47166 -2.60175

H -4.00183 -2.85505 -1.42024

O -4.38673 -0.87008 -1.61937

H -5.14498 -0.30783 -1.81597

C -8.30081 0.95066 0.04656

C -7.53317 3.60576 0.23049

C -9.26517 1.94507 -0.07634

C -6.96404 1.26032 0.26365

C -6.57041 2.60171 0.33798

C -8.87056 3.27147 0.02318

H -10.30380 1.69091 -0.23977

H -6.22219 0.48225 0.36103

H -7.23584 4.64858 0.28586

O -9.84400 4.21499 -0.09449

H -9.45895 5.09087 -0.00682

C -5.15502 2.97225 0.48981

H -4.96837 4.00002 0.78932

C -4.12675 2.15405 0.23165

H -4.32559 1.14868 -0.13038

C -2.69908 2.46883 0.34362

C 0.05334 2.95625 0.48401

C -1.78885 1.57514 -0.22319

C -2.20736 3.61574 0.98430

C -0.84666 3.85401 1.05294

C -0.42279 1.81850 -0.16552

H -2.13613 0.68130 -0.73207

H -2.88966 4.31374 1.45327

H -0.44799 4.72372 1.56106

O 1.38907 3.22040 0.57826

O 0.42585 0.91614 -0.73801

H 2.16892 1.39960 1.14881

C 2.38666 -3.35210 1.00491

H 2.54784 -2.59414 1.77545

H 2.55397 -4.34440 1.41767

H 1.36956 -3.28845 0.60854

H 1.75341 2.11069 -1.78085

O 5.19595 -2.98025 -1.90065

H 5.06402 -3.62905 -1.19374

astringin-atringin 8-10 RS quinone methide

100

Energy: -1821808.0957015

C -0.59383 -2.73757 -0.86219

C -2.27325 -4.16089 0.27938

C -0.06711 -3.55923 1.22796

C -1.13930 -4.64086 1.16723

O 0.42159 -3.32599 -0.08364

C -1.73268 -3.72630 -1.07406

H -2.74694 -3.29238 0.76035

H -0.53097 -2.64096 1.60779

H -0.70634 -5.55610 0.74115

H -1.34304 -4.59496 -1.61871

H -0.95695 -1.83645 -0.34358

O -0.09076 -2.37718 -2.11153

O -2.80398 -3.11997 -1.77393

H -2.51399 -2.94189 -2.67473

O -3.20580 -5.21678 0.17522

H -3.95766 -4.90683 -0.33941

O -1.59080 -4.87818 2.48981

H -2.36618 -5.44860 2.44151

C 1.08379 -3.88933 2.16497

H 1.92917 -3.24341 1.92453

H 1.39448 -4.93427 2.06392

O 0.70971 -3.59981 3.50657

H -0.07784 -4.12120 3.70963

C 0.53171 -1.14136 -2.10287

C 1.53424 1.49661 -1.83180

C -0.26401 -0.04385 -2.60751

C 1.79916 -1.01642 -1.66412

C 2.42917 0.28141 -1.65815

C 0.22612 1.20467 -2.51516

H -1.21815 -0.25241 -3.07747

H 2.32201 -1.90672 -1.34544

O -0.38616 2.30888 -2.96638

H -1.32780 2.14377 -3.16053

C 3.75559 0.49338 -1.42593

H 4.10369 1.52097 -1.46989

C 4.71119 -0.52678 -1.12426

H 4.34490 -1.54851 -1.09519

C 6.04526 -0.36444 -0.86291

C 8.84177 -0.08809 -0.30316

C 6.85651 -1.52719 -0.56864

C 6.69477 0.93467 -0.86366

C 8.00878 1.07356 -0.60197

C 8.17163 -1.40755 -0.30469

H 6.39387 -2.50786 -0.56214

H 6.10472 1.81586 -1.08279

H 8.50048 2.03862 -0.60082

O 10.03961 -0.03483 -0.05654

O 8.96745 -2.45284 -0.02675

H 9.85368 -2.08424 0.12174

C -3.43354 -0.18953 0.46125

C -5.46231 -1.29452 -0.39659

C -4.23090 0.48595 -1.62929

C -5.59503 -0.15533 -1.39462

O -3.68574 0.91192 -0.38510

C -4.76016 -0.81868 0.86356

H -4.84864 -2.08461 -0.85315

H -3.55816 -0.25554 -2.08552

H -6.27255 0.59933 -0.96924

H -5.36482 -0.05685 1.37136

H -2.81928 -0.92346 -0.08145

O -2.78066 0.22539 1.61742

O -4.57283 -1.95412 1.68415

H -4.18143 -1.66558 2.51531

O -6.76676 -1.77008 -0.13320

H -6.70681 -2.45055 0.54522

O -6.07472 -0.61454 -2.63768

H -6.87206 -1.12871 -2.46992

C -4.31341 1.71368 -2.51443

H -5.06607 2.40397 -2.11914

H -4.60044 1.42266 -3.52352

O -3.05252 2.36107 -2.59245

H -2.84571 2.69614 -1.71160

C -1.40247 0.33974 1.53223

C 1.35604 0.51968 1.46997

C -0.65629 -0.36181 2.47304

C -0.79783 1.14000 0.57273

C 0.59593 1.24327 0.55804

C 0.73615 -0.29192 2.42299

H -1.15996 -0.95981 3.22473

H -1.40837 1.71108 -0.11285

H 2.43911 0.57394 1.46519

O 1.53080 -0.97125 3.27720

H 1.07766 -1.75081 3.64353

C 1.29508 2.17293 -0.42108

C 0.65714 3.53402 -0.58163

H 0.65034 3.92093 -1.59527

C 0.12260 4.33873 0.36534

C -1.07752 6.10364 2.28274

C -0.46523 5.60754 -0.05229

C 0.10597 4.02032 1.79374

C -0.44809 4.84794 2.69489

C -1.04096 6.43283 0.83665

H -0.44953 5.87190 -1.10382

H 0.57239 3.10311 2.12907

H -0.45275 4.62789 3.75545

O -1.61565 6.88545 3.05082

O -1.61605 7.59950 0.50138

H -1.94205 7.99018 1.32815

H 2.30969 2.33369 -0.03639

H 2.05624 2.24813 -2.43300

astringin-astringin 8-10 SS quinone methide

100

Energy: -1821808.5330003

C -3.18552 -2.42552 0.57132

C -4.65268 -2.58012 -1.38555

C -4.34910 -0.47889 -0.06459

C -5.31120 -1.28339 -0.94995

O -3.88793 -1.28870 1.01154

C -4.12719 -3.34586 -0.18594

H -3.80280 -2.33414 -2.04105

H -3.49402 -0.14609 -0.67689

H -6.20600 -1.52539 -0.35685

H -4.95210 -3.62200 0.48102

H -2.34485 -2.11574 -0.06949

O -2.68949 -3.11970 1.67481

O -3.42276 -4.50571 -0.59815

H -4.00027 -4.96938 -1.21825

O -5.55000 -3.43595 -2.06074

H -5.92482 -2.94426 -2.79972

O -5.66124 -0.60275 -2.13383

H -5.98290 0.27354 -1.87774

C -5.04062 0.72325 0.57037

H -4.33498 1.25611 1.21579

H -5.85891 0.36116 1.19730

O -5.59506 1.57475 -0.41233

H -4.87257 2.12604 -0.75649

C -1.47182 -2.74404 2.14261

C 1.31237 -2.41530 2.52350

C -0.71101 -3.86096 2.67572

C -0.98793 -1.48342 2.08248

C 0.37645 -1.22534 2.48530

C 0.60398 -3.69686 2.85786

H -1.22260 -4.80170 2.84178

H -1.61316 -0.68494 1.70821

O 1.46305 -4.65195 3.27767

H 0.99540 -5.48002 3.43031

C 0.85257 0.02751 2.72091

H 1.90975 0.15421 2.92910

C 0.00574 1.18651 2.75998

H -1.05057 0.99679 2.93724

C 0.36726 2.49834 2.62621

C 0.98494 5.21403 1.94859

C -0.64045 3.53287 2.73775

C 1.70773 2.89163 2.23054

C 2.00327 4.16459 1.89696

C -0.36614 4.80918 2.40305

H -1.64215 3.26363 3.05637

H 2.47400 2.12729 2.16084

H 2.99351 4.46333 1.57373

O 1.18522 6.38041 1.64151

O -1.27779 5.78857 2.44831

H -0.84924 6.57513 2.07321

C 0.73132 1.61076 -1.79823

C 0.93523 4.07040 -1.98593

C -0.79444 2.94852 -0.61924

C -0.48934 4.18040 -1.46649

O -0.60965 1.76708 -1.39732

C 1.14112 2.75377 -2.71530

H 1.61686 4.09745 -1.12122

H -0.10682 2.94086 0.23172

H -1.18243 4.22975 -2.31720

H 0.50195 2.72144 -3.60704

H 1.37466 1.59167 -0.90576

O 0.83875 0.43027 -2.52773

O 2.50686 2.67723 -3.05761

H 2.65112 1.87236 -3.56534

O 1.16982 5.18050 -2.82372

H 2.07459 5.12538 -3.14841

O -0.63698 5.30484 -0.63004

H -0.19754 6.05190 -1.05259

C -2.21467 2.88534 -0.10062

H -2.48204 3.83140 0.37156

H -2.27056 2.07688 0.63998

O -3.13167 2.64512 -1.16176

H -2.71623 2.00867 -1.75813

C 0.66958 -0.74914 -1.82651

C 0.36217 -3.20189 -0.57799

C -0.24654 -1.65290 -2.33421

C 1.42647 -1.03948 -0.69679

C 1.27125 -2.27278 -0.06701

C -0.40485 -2.88280 -1.70036

H -0.83291 -1.41001 -3.21086

H 2.16010 -0.33041 -0.33574

H 0.24326 -4.17469 -0.11144

O -1.31405 -3.73834 -2.23343

H -1.81788 -4.19674 -1.53996

C 2.10450 -2.61786 1.16586

C 3.41545 -1.86955 1.22734

H 3.55314 -1.18157 2.05759

C 4.43963 -1.98717 0.35588

C 6.59017 -2.17077 -1.52241

C 5.65217 -1.20250 0.54310

C 4.36345 -2.87326 -0.80542

C 5.36894 -2.96402 -1.68966

C 6.66469 -1.28016 -0.33537

H 5.73018 -0.54692 1.40322

H 3.46160 -3.45719 -0.95055

H 5.32596 -3.61227 -2.55630

O 7.53547 -2.20272 -2.29250

O 7.80149 -0.57776 -0.21924

H 8.35478 -0.82015 -0.97924

H 2.33900 -3.68677 1.11523

H 2.06455 -2.26462 3.30199

astringin-astringin 8-10 RR

100

Energy: -1821859.8817111

C 2.11257 -4.16003 1.02484

C 4.35107 -4.89421 0.32466

C 2.84298 -3.54039 -1.11923

C 3.95445 -4.58374 -1.11302

O 1.76085 -4.02410 -0.32961

C 3.13818 -5.27119 1.16318

H 4.80048 -3.98935 0.76087

H 3.21440 -2.59450 -0.69917

H 3.57826 -5.50815 -1.57456

H 2.69408 -6.19944 0.78221

H 2.53176 -3.20976 1.38890

O 0.96730 -4.49792 1.75131

O 3.58653 -5.42182 2.49234

H 2.83673 -5.67512 3.04026

O 5.30586 -5.93305 0.27905

H 5.54370 -6.15970 1.18426

O 5.04596 -4.06850 -1.84038

H 5.78326 -4.67819 -1.72679

C 2.26109 -3.27281 -2.49313

H 1.93434 -4.21665 -2.94400

H 3.01591 -2.81457 -3.13120

O 1.17026 -2.36812 -2.39717

H 0.57195 -2.72109 -1.72600

C -0.02864 -3.53606 1.62834

C -1.88257 -1.56712 1.13288

C -1.18302 -3.89058 0.93550

C 0.18511 -2.25803 2.12142

C -0.73657 -1.23555 1.85541

C -2.09979 -2.87649 0.71715

H -1.31384 -4.88973 0.54336

H 1.08554 -2.04975 2.68616

O -3.25302 -3.03087 0.01627

C -0.48585 0.17068 2.19597

H -1.35852 0.81591 2.17334

C 0.70810 0.73888 2.40604

H 1.60890 0.13176 2.45622

C 0.89386 2.19959 2.45453

C 1.24969 4.97332 2.38459

C 0.04811 3.01310 1.68945

C 1.94601 2.80118 3.14979

C 2.10892 4.18322 3.13534

C 0.23988 4.37809 1.61972

H -0.72747 2.56949 1.08269

H 2.63106 2.18858 3.72499

H 2.90142 4.66448 3.69506

O -0.52320 5.17239 0.78605

H 0.09103 5.49994 0.09480

C 1.48782 1.90914 -1.40531

C 3.94502 1.72514 -1.21591

C 2.74183 3.87207 -0.92537

C 4.03181 3.22296 -1.42540

O 1.62502 3.30193 -1.59532

C 2.71629 1.15518 -1.89789

H 3.85573 1.53355 -0.13478

H 2.66141 3.70584 0.16131

H 4.14339 3.42493 -2.50097

H 2.79305 1.29052 -2.98466

H 1.32324 1.68920 -0.33898

O 0.39309 1.47655 -2.16004

O 2.66990 -0.20682 -1.54526

H 1.94990 -0.67538 -1.99969

O 5.13843 1.15280 -1.70915

H 5.04078 0.19471 -1.67282

O 5.11132 3.77224 -0.70106

H 5.88257 3.22256 -0.87902

C 2.73495 5.35984 -1.22265

H 2.51082 5.49814 -2.28211

H 3.72470 5.76951 -1.01115

O 1.73951 6.06427 -0.48633

H 2.11576 6.35295 0.35441

C -0.81343 1.59058 -1.49870

C -3.11421 1.76893 0.02602

C -1.41982 2.83107 -1.34372

C -1.34709 0.44493 -0.93384

C -2.50103 0.53204 -0.15538

C -2.55923 2.91635 -0.54678

H -0.95948 3.71433 -1.76882

H -0.80634 -0.48859 -1.04335

H -3.97927 1.87628 0.67120

O -3.12003 4.12050 -0.24661

H -2.40180 4.74205 -0.05638

C -2.99297 -0.70363 0.56951

C -3.68534 -1.72144 -0.39540

C -5.18272 -1.61639 -0.40304

C -7.94940 -1.24665 -0.33925

C -5.76434 -0.55763 -1.10635

C -5.98920 -2.48442 0.32086

C -7.37046 -2.30112 0.34663

C -7.13416 -0.37147 -1.06689

H -5.14347 0.12394 -1.68263

H -5.53850 -3.31047 0.85537

H -8.01683 -2.97222 0.89863

O -7.79756 0.63397 -1.71765

H -7.17517 1.23802 -2.13100

H -3.69962 -0.40876 1.35228

H -3.29016 -1.55900 -1.40499

O -9.29350 -1.06757 -0.31001

H -9.50948 -0.28636 -0.83244

O 1.44148 6.32882 2.33455

H 0.60290 6.73850 2.08389

astringin-astringin 8-10 RS

100

Energy: -1821863.7234433

C -3.97178 2.27306 0.93855

C -6.00376 2.11565 -0.44861

C -3.71305 1.77485 -1.34978

C -5.12047 2.26037 -1.67935

O -3.22185 2.50554 -0.22991

C -5.37964 2.81260 0.74936

H -6.10047 1.04487 -0.21464

H -3.73703 0.70061 -1.11400

H -5.07579 3.32555 -1.94940

H -5.30779 3.89056 0.55597

H -3.99465 1.19225 1.15206

O -3.37677 2.95838 2.00102

O -6.20385 2.54428 1.86195

H -5.83536 2.99505 2.62849

O -7.26694 2.65547 -0.77468

H -7.82464 2.60334 0.00867

O -5.61196 1.49202 -2.75278

H -6.54512 1.70721 -2.85875

C -2.71707 2.01464 -2.46533

H -2.69587 3.08205 -2.71221

H -3.01135 1.44968 -3.34932

O -1.42344 1.56867 -2.08563

H -1.20914 1.97001 -1.23348

C -2.11635 2.46011 2.30499

C 0.37408 1.38507 2.70835

C -2.02110 1.32981 3.11253

C -1.01024 3.08086 1.74418

C 0.26838 2.53847 1.92868

C -0.74662 0.81467 3.28966

H -2.89787 0.86215 3.54152

H -1.16709 3.96869 1.14515

O -0.48044 -0.31922 3.98160

C 1.47106 3.06802 1.27744

H 2.40993 2.75763 1.72504

C 1.51846 3.80862 0.16290

H 0.59805 4.15965 -0.29892

C 2.76681 4.14482 -0.53492

C 5.15640 4.73523 -1.88152

C 3.89172 3.32004 -0.39529

C 2.85787 5.25404 -1.37613

C 4.04739 5.55324 -2.03495

C 5.06654 3.60399 -1.05923

H 3.83533 2.42164 0.20662

H 1.99590 5.89758 -1.51065

H 4.13042 6.41639 -2.68368

O 6.18787 2.82584 -0.98797

H 5.99180 2.00602 -0.51153

C 0.24700 -2.21875 -1.48988

C -1.74490 -3.46734 -2.25945

C 0.37221 -4.56111 -1.63072

C -0.83394 -4.64572 -2.55626

O 1.05080 -3.32702 -1.83834

C -0.99068 -2.15277 -2.37383

H -2.10535 -3.56563 -1.22393

H 0.01725 -4.61074 -0.59368

H -0.49895 -4.58602 -3.60178

H -0.66303 -2.00081 -3.41097

H -0.04748 -2.30095 -0.43278

O 1.00081 -1.06611 -1.71203

O -1.87143 -1.14180 -1.95270

H -1.47183 -0.26123 -2.07147

O -2.83140 -3.53012 -3.15966

H -3.38002 -2.75258 -3.00884

O -1.48653 -5.87129 -2.30701

H -2.34965 -5.82836 -2.73347

C 1.39667 -5.65089 -1.85639

H 1.76894 -5.60237 -2.88510

H 0.94427 -6.62694 -1.68388

O 2.47462 -5.50830 -0.93486

H 2.76981 -4.59076 -0.99001

C 1.83110 -0.68834 -0.68418

C 3.52090 0.14855 1.34311

C 3.19219 -0.57130 -0.94653

C 1.30471 -0.36023 0.55337

C 2.15340 0.05018 1.57789

C 4.02318 -0.13504 0.07515

H 3.56325 -0.78714 -1.94204

H 0.23166 -0.39961 0.70807

H 4.20310 0.48469 2.11681

O 5.36385 0.09499 -0.13684

H 5.63407 -0.32379 -0.96065

C 1.55478 0.45800 2.91046

C 0.88747 -0.70732 3.74965

C 0.92616 -2.07950 3.11291

C 1.05752 -4.59666 1.91575

C -0.23376 -2.68434 2.63841

C 2.14961 -2.73509 2.98328

C 2.21517 -3.98762 2.38397

C -0.16979 -3.93924 2.04352

H -1.19725 -2.20075 2.73753

H 3.05829 -2.26863 3.34874

H 3.15848 -4.51488 2.29441

O -1.30526 -4.52232 1.57935

H -1.07692 -5.40984 1.26993

H 2.33628 0.92575 3.51574

O 1.02360 -5.84993 1.36642

H 1.71106 -5.91075 0.67668

O 6.30954 5.01201 -2.53756

H 6.95190 4.32627 -2.31893

H 1.36730 -0.75646 4.72899

isorhapontin-isorhapontin 8-10 RS quinone methide

106

Energy: -1871112.8478686

C 2.29645 2.07514 -0.74459

C 2.64264 4.36678 0.17802

C 0.55078 3.64498 -0.85519

C 1.47666 4.84021 -0.67085

O 1.27971 2.63481 -1.53805

C 3.33754 3.13530 -0.39254

H 2.24077 4.08777 1.16463

H 0.25717 3.28703 0.14401

H 1.84280 5.17303 -1.65175

H 3.86625 3.40646 -1.31559

H 1.86970 1.62557 0.16866

O 2.93313 1.07370 -1.49449

O 4.22013 2.72571 0.62179

H 4.80232 1.99802 0.35686

O 3.54478 5.44320 0.32219

H 4.29139 5.12502 0.84156

O 0.77298 5.87289 -0.01634

H 1.42386 6.52033 0.27625

C -0.72671 3.91555 -1.62304

H -0.51374 3.92931 -2.69314

H -1.12128 4.89022 -1.32177

O -1.67694 2.88351 -1.39374

H -1.93964 2.96344 -0.46481

C 2.22151 -0.05347 -1.76393

C 1.18636 -2.67505 -1.76898

C 3.03167 -1.11956 -2.33437

C 0.89263 -0.20161 -1.54283

C 0.27566 -1.48083 -1.81523

C 2.52679 -2.36228 -2.36220

H 4.03946 -0.88653 -2.65974

H 0.28670 0.64305 -1.25242

O 3.18427 -3.46879 -2.78249

H 4.09611 -3.24484 -3.00170

C -1.05936 -1.64862 -2.00305

H -1.43151 -2.66239 -2.11425

C -1.99301 -0.55756 -2.01989

H -1.58405 0.44480 -2.12808

C -3.34780 -0.63649 -1.86650

C -6.13209 -0.65053 -1.12586

C -4.10748 0.59916 -1.80711

C -4.05234 -1.88374 -1.62390

C -5.35419 -1.89192 -1.29178

C -5.39627 0.61477 -1.40611

H -3.55980 1.51451 -2.00523

H -3.50780 -2.81877 -1.69331

H -5.89538 -2.81169 -1.10113

O -7.29404 -0.65673 -0.77643

O -6.13441 1.71798 -1.17697

C -2.61057 -0.72138 1.73723

C -3.62948 1.48469 1.58483

C -1.17840 1.13585 1.88894

C -2.34666 2.08980 2.13044

O -1.50412 -0.14648 2.40747

C -3.84979 0.07474 2.11635

H -3.52288 1.40215 0.49344

H -0.99310 1.05800 0.80279

H -2.45809 2.26730 3.20789

H -3.94133 0.08181 3.21031

H -2.44851 -0.67891 0.65038

O -2.75880 -2.04437 2.13611

O -5.01924 -0.39743 1.50025

H -5.18994 -1.30264 1.77986

O -4.68823 2.35958 1.89727

H -5.49829 1.98859 1.52257

O -2.05691 3.30750 1.45756

H -2.78599 3.91569 1.62894

C 0.10683 1.54201 2.58614

H -0.08457 1.65549 3.65952

H 0.46524 2.49538 2.19721

O 1.11453 0.57566 2.35275

H 0.79720 -0.25059 2.73613

C -1.91921 -2.95995 1.54517

C -0.32758 -4.90386 0.37309

C -2.35028 -4.27926 1.54868

C -0.70116 -2.59993 0.97204

C 0.09950 -3.57909 0.38640

C -1.54800 -5.24676 0.95736

H -3.29707 -4.55086 1.99644

H -0.38271 -1.56783 0.97603

H 0.29267 -5.67373 -0.07721

O -2.00985 -6.52484 0.97726

H -1.38175 -7.10851 0.54345

C 1.41933 -3.21269 -0.25868

C 2.21034 -2.21147 0.51873

H 1.66856 -1.37129 0.93985

C 3.56143 -2.14055 0.55955

C 6.37303 -1.65485 0.13750

C 4.17759 -0.88839 0.97828

C 4.44264 -3.17876 0.03991

C 5.75681 -2.96386 -0.13582

C 5.47548 -0.63440 0.73489

H 3.54299 -0.10432 1.38710

H 4.02395 -4.14880 -0.19935

H 6.42338 -3.73229 -0.51045

O 7.53896 -1.41853 -0.12315

O 5.97665 0.62096 0.92086

H 1.99991 -4.12654 -0.39605

H 0.74281 -3.51781 -2.30368

C 7.01330 0.74764 1.89909

H 7.24871 1.80862 1.95078

H 7.89238 0.17996 1.59743

H 6.64496 0.40588 2.86994

C -5.52251 2.96321 -1.45657

H -4.64325 3.12101 -0.82178

H -6.27072 3.72213 -1.24073

H -5.22145 3.02338 -2.50657

isorhapontin-isorhapontin 8-10 SS quinone methide

106

Energy: -1870558.2588089

C -2.78713 -1.40876 -0.15260

C -2.89269 -3.90704 -0.08251

C -2.44165 -2.55609 1.95332

C -2.09148 -3.83872 1.20560

O -2.22484 -1.41259 1.13119

C -2.55210 -2.70504 -0.94081

H -3.96963 -3.89758 0.15729

H -3.49504 -2.60732 2.27173

H -1.01688 -3.83803 0.95722

H -1.50375 -2.78784 -1.24415

H -3.86962 -1.22581 -0.10002

O -2.27961 -0.28511 -0.83758

O -3.39309 -2.74587 -2.07836

H -3.04921 -2.13641 -2.74783

O -2.54489 -5.11853 -0.71866

H -2.93686 -5.14529 -1.60691

O -2.40180 -4.91401 2.06147

H -2.26353 -5.73467 1.56212

C -1.55387 -2.32153 3.16550

H -1.55003 -3.21133 3.79825

H -1.93949 -1.46521 3.73408

O -0.20945 -2.07817 2.76676

H -0.24133 -1.40917 2.05640

C -0.92860 -0.07576 -0.93793

C 1.75679 0.93006 -0.98228

C -0.64416 1.16437 -1.63993

C 0.05551 -0.88260 -0.47473

C 1.44978 -0.51452 -0.63929

C 0.61705 1.63155 -1.67010

H -1.47030 1.71911 -2.06877

H -0.15645 -1.82367 0.01161

O 0.99549 2.80605 -2.20498

H 0.21198 3.30966 -2.52131

C 2.46965 -1.38321 -0.40036

H 3.48810 -0.99325 -0.40699

C 2.32003 -2.78368 -0.02524

H 2.88680 -3.11428 0.84718

C 1.56848 -3.72465 -0.66761

C 0.00052 -5.74395 -2.01471

C 1.38324 -5.04637 -0.09288

C 0.95426 -3.46520 -1.96417

C 0.22170 -4.40530 -2.59019

C 0.62568 -5.99680 -0.68681

H 1.84294 -5.26972 0.86694

H 1.13444 -2.50045 -2.43013

H -0.20964 -4.24308 -3.57442

O -0.63183 -6.60198 -2.61792

O 0.53401 -7.21654 -0.10931

C -2.87715 3.44781 0.77528

C -5.12582 4.02253 -0.11437

C -3.06687 4.09254 -1.49921

C -4.43670 4.73267 -1.26473

O -2.28804 4.13993 -0.31093

C -4.23575 4.05569 1.11043

H -5.29547 2.97112 -0.40262

H -3.23261 3.04503 -1.80638

H -4.30241 5.79384 -0.99792

H -4.06920 5.09922 1.41758

H -2.96567 2.37499 0.52834

O -2.09474 3.61631 1.91863

O -4.89338 3.32724 2.12341

H -4.34450 3.35919 2.92227

O -6.36156 4.67061 0.10131

H -6.77787 4.27432 0.88305

O -5.18103 4.60811 -2.45701

H -6.10040 4.84393 -2.25256

C -2.30087 4.82634 -2.58719

H -1.80702 5.69549 -2.14436

H -2.99941 5.16412 -3.35798

O -1.27545 4.02501 -3.17471

H -1.63099 3.55032 -3.93882

C -1.03925 2.73372 2.07493

C 1.01486 0.90862 2.40028

C -1.07591 1.86988 3.16049

C 0.02214 2.73748 1.17802

C 1.05173 1.80565 1.32931

C -0.03319 0.95947 3.32534

H -1.90252 1.88521 3.86299

H 0.02975 3.45813 0.36650

H 1.79702 0.16760 2.54599

O -0.07329 0.13982 4.41176

H 0.29144 -0.72952 4.15664

C 2.15178 1.80644 0.28437

C 3.50485 1.41930 0.80465

H 3.54787 0.76819 1.67483

C 4.68744 1.79791 0.26260

C 7.24434 2.45194 -0.91054

C 5.93632 1.33375 0.85372

C 4.78120 2.65436 -0.91913

C 5.97259 2.96342 -1.46037

C 7.14314 1.63512 0.33202

H 5.89286 0.70894 1.74265

H 3.86869 3.03890 -1.36812

H 6.06186 3.58751 -2.34446

O 8.31462 2.67699 -1.45683

O 8.25972 1.10253 0.88185

H 2.21391 2.82697 -0.10630

H 2.62374 0.97020 -1.65181

C -0.69757 -7.94015 -0.18654

H -0.70902 -8.58578 -1.06530

H -1.55157 -7.25892 -0.23370

H -0.73852 -8.53923 0.72567

C 9.38243 1.96484 1.08454

H 9.98316 1.48244 1.85674

H 9.05203 2.94660 1.44106

H 9.95712 2.08052 0.16600

isorhapontin-isorhapontin 8-10 SS

106

Energy: -1871172.3472811

C -2.39063 -4.04951 0.25602

C -4.49058 -5.05053 -0.54199

C -4.32429 -2.73489 0.34587

C -5.24228 -3.94611 0.18620

O -3.14944 -3.14827 1.02729

C -3.16609 -5.35179 0.13766

H -4.27837 -4.70404 -1.56458

H -4.05370 -2.36302 -0.65150

H -5.52495 -4.31344 1.18339

H -3.34436 -5.73527 1.15065

H -2.18908 -3.60922 -0.73111

O -1.18101 -4.32792 0.90613

O -2.49913 -6.30811 -0.65897

H -1.64303 -6.48296 -0.25568

O -5.33718 -6.18248 -0.58320

H -4.85053 -6.89869 -1.00444

O -6.38517 -3.55460 -0.54382

H -6.86600 -4.35706 -0.77387

C -4.95976 -1.61709 1.15040

H -5.07893 -1.96079 2.18173

H -5.95004 -1.40866 0.73557

O -4.15043 -0.45571 1.17902

H -4.41176 0.13992 0.46212

C -0.23056 -3.32812 0.80152

C 1.64985 -1.32359 0.51113

C 0.97988 -3.65017 0.18773

C -0.49263 -2.05987 1.29516

C 0.44218 -1.02432 1.14742

C 1.89856 -2.62343 0.07403

H 1.17603 -4.64405 -0.19110

H -1.44156 -1.88524 1.77832

O 3.13515 -2.77684 -0.47946

C 0.13092 0.34281 1.58904

H 0.96752 1.03329 1.62480

C -1.11021 0.77712 1.85595

H -1.94365 0.07910 1.81654

C -1.51388 2.16656 2.08860

C -2.45567 4.80072 2.24321

C -0.64820 3.24650 1.83195

C -2.83673 2.43419 2.44366

C -3.30262 3.74586 2.53260

C -1.11435 4.54413 1.89877

H 0.37360 3.05723 1.52476

H -3.51258 1.60319 2.61475

H -4.33090 3.96382 2.79436

O -0.38590 5.66171 1.62772

C -0.84724 2.55137 -1.62783

C -3.14761 3.22588 -1.13656

C -2.48332 0.85849 -1.57632

C -3.60782 1.88676 -1.68349

O -1.30547 1.35436 -2.21584

C -1.87385 3.65749 -1.83744

H -2.92645 3.10908 -0.06731

H -2.27528 0.65963 -0.51400

H -3.89283 2.01091 -2.73759

H -2.05142 3.77925 -2.91342

H -0.68414 2.39536 -0.55406

O 0.34716 2.95140 -2.23294

O -1.45528 4.86929 -1.24617

H -0.70735 5.20371 -1.75046

O -4.21337 4.13578 -1.31340

H -3.97815 4.96003 -0.87384

O -4.71503 1.40423 -0.93822

H -5.32650 2.14219 -0.82408

C -2.83877 -0.44245 -2.26371

H -2.81601 -0.28015 -3.34835

H -3.85183 -0.73074 -1.97952

O -1.98422 -1.50125 -1.88443

H -1.07280 -1.23061 -2.03864

C 1.50329 2.49845 -1.64126

C 3.82438 1.74565 -0.33608

C 2.58071 3.37368 -1.62309

C 1.58163 1.23484 -1.06326

C 2.74363 0.86673 -0.39018

C 3.74573 2.98361 -0.97162

H 2.51186 4.34893 -2.08787

H 0.73742 0.55981 -1.11591

H 4.73321 1.45648 0.18418

O 4.77636 3.87083 -0.96274

H 5.53486 3.48293 -0.51695

C 2.89092 -0.50537 0.22526

C 3.63627 -1.46024 -0.76343

C 5.13099 -1.41960 -0.65398

C 7.89272 -1.19349 -0.33812

C 5.76070 -2.04582 0.42900

C 5.88601 -0.70010 -1.56773

C 7.26752 -0.58051 -1.40755

C 7.13137 -1.93785 0.58184

H 5.16322 -2.62463 1.12249

H 5.39885 -0.22106 -2.40977

H 7.87384 -0.02662 -2.11351

O 7.87046 -2.50609 1.57770

H 3.48942 -0.41327 1.14029

C 7.18402 -3.29500 2.53194

H 6.68170 -4.13741 2.04870

H 6.45119 -2.69384 3.07756

H 7.93941 -3.66436 3.22111

C 0.90397 5.47208 1.07542

H 0.84742 4.86015 0.17009

H 1.57201 4.99229 1.79672

H 1.28280 6.46275 0.83451

O 9.23365 -1.08871 -0.17846

H 9.48321 -1.59735 0.60259

O -2.91208 6.07612 2.26308

H -2.18242 6.65472 2.00798

H 3.32530 -1.20058 -1.78228

isorhapontin-isorhapontin 8-10 RS

106

Energy: -1871169.5043055

C -3.87771 -1.57585 -1.53116

C -6.30150 -1.61011 -1.03881

C -4.54584 -1.85528 0.70006

C -5.95428 -2.27204 0.27928

O -3.62422 -2.24170 -0.30971

C -5.24798 -1.96432 -2.07067

H -6.30130 -0.51872 -0.89751

H -4.53587 -0.76396 0.81969

H -5.98004 -3.36249 0.14676

H -5.24909 -3.04875 -2.24309

H -3.81989 -0.48611 -1.38181

O -2.94005 -1.99711 -2.47218

O -5.56495 -1.25905 -3.24882

H -4.88701 -1.44892 -3.90578

O -7.58958 -2.05378 -1.41102

H -7.78726 -1.69548 -2.28293

O -6.85837 -1.88302 1.30077

H -7.75295 -2.01126 0.96525

C -4.11967 -2.49770 2.01569

H -3.04374 -2.37085 2.14871

H -4.33163 -3.57284 1.99520

O -4.76034 -1.87279 3.11764

H -5.71146 -1.90273 2.94850

C -1.67273 -1.45220 -2.34871

C 0.90480 -0.46967 -2.15022

C -1.48208 -0.08689 -2.55585

C -0.62292 -2.31289 -2.07413

C 0.69385 -1.83693 -1.94517

C -0.17421 0.35705 -2.45844

H -2.30207 0.58561 -2.78252

H -0.84490 -3.36274 -1.92742

O 0.17845 1.66177 -2.61654

C 1.73625 -2.80542 -1.58194

H 1.47628 -3.84437 -1.76826

C 2.90372 -2.52128 -0.98933

H 3.12040 -1.49634 -0.71419

C 3.93037 -3.48341 -0.57747

C 5.91778 -5.23693 0.32884

C 4.87101 -3.06072 0.37704

C 4.01703 -4.78287 -1.07630

C 5.00299 -5.65307 -0.62498

C 5.84931 -3.92765 0.83018

H 4.80581 -2.05006 0.76296

H 3.32410 -5.11839 -1.83827

H 5.08420 -6.66250 -1.00879

O 6.80668 -3.63562 1.75910

C -0.74957 1.06383 2.13762

C -3.10446 0.80915 2.78004

C -2.37737 1.68065 0.57422

C -3.43159 1.80078 1.67015

O -1.12815 2.01239 1.16357

C -1.70599 1.07489 3.32512

H -3.09045 -0.19534 2.34366

H -2.35159 0.64255 0.19964

H -3.39643 2.82338 2.07150

H -1.66830 2.05779 3.81178

H -0.75470 0.05415 1.69721

O 0.53070 1.38320 2.58757

O -1.38780 0.03604 4.21845

H -0.50897 0.20044 4.57379

O -4.11079 0.87944 3.75855

H -4.33162 -0.03191 3.99456

O -4.71349 1.53996 1.12632

H -5.31447 1.41915 1.87360

C -2.60931 2.59615 -0.61802

H -1.71347 2.59051 -1.24403

H -2.78564 3.62281 -0.27210

O -3.67988 2.12652 -1.41317

H -4.46360 2.07995 -0.85231

C 1.55166 1.25699 1.67291

C 3.66625 1.17106 -0.12277

C 2.80385 1.69285 2.08239

C 1.34703 0.76470 0.38768

C 2.40029 0.74711 -0.51669

C 3.85810 1.64153 1.17527

H 2.95692 2.08811 3.07808

H 0.36694 0.46327 0.05216

H 4.48658 1.18470 -0.83496

O 5.07010 2.07887 1.61510

H 5.66471 2.18425 0.86695

C 2.11341 0.42485 -1.97400

C 1.61253 1.70455 -2.73598

C 2.14743 3.00895 -2.22536

C 3.35013 5.24411 -1.07365

C 1.54886 3.62798 -1.12145

C 3.31928 3.52664 -2.75574

C 3.92486 4.64452 -2.17892

C 2.14907 4.73407 -0.54796

H 0.63871 3.21299 -0.70633

H 3.77750 3.05490 -3.61881

H 4.83912 5.06452 -2.57966

O 1.67881 5.41964 0.53143

H 3.01414 0.04080 -2.45840

C 6.78893 -2.34031 2.32882

H 7.60839 -2.31022 3.04272

H 5.84305 -2.15509 2.84547

H 6.94214 -1.57576 1.56175

C 0.53863 4.88732 1.18774

H 0.73051 3.87087 1.53867

H -0.32560 4.87729 0.51746

H 0.34171 5.54479 2.03143

O 6.87872 -6.08395 0.76759

H 7.41136 -5.61767 1.42329

O 3.93292 6.32494 -0.50051

H 3.39128 6.58719 0.25409

H 1.85688 1.58135 -3.79692

astringin-astringin 8-5 RS quinone methide

100

Energy: -1821821.3193215

O 4.65877 3.06096 0.40060

C 3.61222 3.90711 0.11431

C 1.64417 5.76297 -0.48025

C 3.95487 5.25076 0.00835

C 2.30469 3.47459 -0.06113

C 1.31477 4.41077 -0.38447

C 2.96139 6.17354 -0.28443

H 4.97587 5.57328 0.16289

H 2.03937 2.43651 0.07856

H 0.87665 6.49224 -0.72113

O 3.33709 7.47849 -0.37366

H 2.57187 8.02396 -0.57361

C -0.07529 3.98936 -0.62631

H -0.82939 4.76401 -0.51997

C -0.41692 2.74248 -0.97316

H 0.37360 2.01072 -1.11613

C -1.76615 2.21290 -1.17684

C -4.36357 1.19438 -1.82377

C -2.94225 3.08853 -1.15126

C -1.91532 0.88952 -1.38217

C -3.25911 0.24220 -1.44655

C -4.16307 2.62169 -1.46589

H -2.83109 4.14373 -0.93342

H -1.04783 0.24050 -1.40489

O -5.42398 0.84198 -2.29563

O -5.26037 3.39626 -1.53634

H -5.98470 2.83302 -1.85182

C 1.11502 -3.62606 -0.43072

C 3.12687 -4.50992 -1.53501

C 2.61809 -2.08823 -1.36729

C 3.30965 -3.15403 -2.20500

O 1.25369 -2.44555 -1.18473

C 1.65967 -4.79176 -1.23869

H 3.66804 -4.49106 -0.57650

H 3.11016 -2.04007 -0.38445

H 2.85138 -3.19020 -3.20328

H 1.09297 -4.85707 -2.17611

H 1.67797 -3.52489 0.50943

O -0.24152 -3.82711 -0.18439

O 1.61493 -6.00551 -0.52236

H 0.69498 -6.21901 -0.33618

O 3.69084 -5.47878 -2.39114

H 3.55246 -6.34544 -1.99428

O 4.67540 -2.80929 -2.28893

H 5.13513 -3.54323 -2.71134

C 2.58427 -0.70833 -1.99193

H 2.11031 -0.76698 -2.97852

H 3.59509 -0.31414 -2.11175

O 1.86456 0.17143 -1.14166

H 1.08403 -0.31484 -0.84243

C -0.83067 -2.78804 0.52526

C -1.97696 -0.65255 1.85090

C -0.31949 -2.40751 1.76001

C -1.91249 -2.14166 -0.04860

C -2.48609 -1.05984 0.62595

C -0.89214 -1.32302 2.42138

H 0.51155 -2.93472 2.21055

H -2.25746 -2.46529 -1.02382

H -2.39271 0.19818 2.37765

O -0.43288 -0.89030 3.61189

H 0.45583 -1.25598 3.76503

C -3.63358 -0.27384 0.01655

C -4.88924 -1.08729 0.00279

H -4.76700 -2.12074 -0.31538

C -6.13628 -0.67141 0.31728

C -8.80853 0.18270 0.90254

C -7.24918 -1.60508 0.22966

C -6.42954 0.70290 0.72123

C -7.67942 1.11043 0.99419

C -8.50452 -1.21301 0.50164

H -7.05385 -2.62915 -0.06695

H -5.61632 1.41620 0.79663

H -7.90727 2.12798 1.28745

O -9.96693 0.49026 1.13491

O -9.56716 -2.03037 0.43183

H -10.34049 -1.49462 0.67111

H -3.28430 -0.61160 -2.12722

H -3.75918 0.62360 0.62397

C 4.40728 1.69575 0.47965

C 5.52063 -0.50226 0.55419

C 3.46916 0.05264 1.88825

C 4.75428 -0.77572 1.83438

O 3.79261 1.42276 1.72081

C 5.75303 0.98818 0.39257

H 4.93337 -0.85443 -0.30457

H 2.78795 -0.27976 1.09277

H 5.39093 -0.49595 2.68527

H 6.39388 1.35844 1.20296

H 3.75463 1.36926 -0.34173

O 6.35437 1.17661 -0.86869

H 6.50314 2.11945 -0.99442

O 6.72302 -1.23886 0.63616

H 7.19338 -1.13585 -0.19764

O 4.40708 -2.15345 1.92093

H 5.20908 -2.66030 1.74327

C 2.76580 -0.05452 3.23404

H 1.91642 0.63000 3.24606

H 3.45896 0.23852 4.03024

O 2.25308 -1.35983 3.46416

H 2.93565 -2.00767 3.24389

astringin-astringin 8-5 SS quinone methide

100

Energy: -1821814.0582463

O 4.84078 1.36860 0.92167

C 4.03404 1.87005 -0.07090

C 2.19135 2.75955 -1.95109

C 4.40209 1.91683 -1.41070

C 2.77443 2.28473 0.34477

C 1.83908 2.70790 -0.60021

C 3.46261 2.36232 -2.34276

H 5.36427 1.55505 -1.75224

H 2.51741 2.20837 1.39491

H 1.48939 3.08368 -2.70871

O 3.74588 2.39751 -3.66905

H 4.60089 1.98998 -3.83724

C 0.45965 2.94792 -0.14988

H 0.34684 3.25191 0.88643

C -0.60871 2.69259 -0.91588

H -0.45297 2.31612 -1.92332

C -2.01014 2.76916 -0.50195

C -4.75338 2.99658 0.30799

C -2.37000 3.31566 0.80819

C -2.96322 2.29965 -1.32852

C -4.40323 2.21286 -0.93729

C -3.65135 3.43229 1.19101

H -1.59964 3.67311 1.47981

H -2.68589 1.89785 -2.29937

O -5.90111 3.23014 0.64263

O -4.02872 3.96715 2.36503

H -4.99752 4.00337 2.35684

C 0.09765 -1.18705 0.69755

C 2.54804 -1.66192 0.37192

C 1.07210 -0.71015 -1.38248

C 2.29591 -1.59253 -1.12522

O -0.06615 -1.24777 -0.70071

C 1.27762 -2.06321 1.10191

H 2.84856 -0.65994 0.72270

H 1.27219 0.30140 -1.00669

H 2.09633 -2.60380 -1.49923

H 1.02001 -3.09284 0.82088

H 0.23496 -0.13925 1.01080

O -1.01060 -1.74418 1.34055

O 1.55349 -1.96879 2.48301

H 0.79321 -2.30975 2.96450

O 3.58019 -2.59226 0.62251

H 3.65391 -2.68260 1.57906

O 3.41218 -1.07657 -1.82055

H 4.03000 -0.66658 -1.19385

C 0.69881 -0.62498 -2.84825

H 0.54112 -1.63636 -3.24385

H 1.51039 -0.15004 -3.39934

O -0.46083 0.16891 -3.02330

H -1.11694 -0.17297 -2.40662

C -2.16626 -1.00162 1.45673

C -4.59815 0.28451 1.78895

C -2.67857 -0.84082 2.73355

C -2.83996 -0.51168 0.33950

C -4.05884 0.13837 0.51130

C -3.90128 -0.19501 2.89391

H -2.15184 -1.23009 3.59495

H -2.42564 -0.67547 -0.64574

H -5.56559 0.76066 1.92214

O -4.36999 -0.07214 4.16152

H -5.20388 0.40617 4.15879

C -4.78763 0.70110 -0.70332

C -6.28728 0.52643 -0.64357

H -6.88099 1.43101 -0.55162

C -6.93657 -0.65659 -0.69112

C -8.33560 -3.15202 -0.77638

C -8.39167 -0.69009 -0.63785

C -6.22167 -1.92872 -0.78424

C -6.87163 -3.10236 -0.82634

C -9.05529 -1.85688 -0.67840

H -8.93733 0.24391 -0.56629

H -5.13780 -1.91279 -0.80617

H -6.35549 -4.05234 -0.89202

O -8.98453 -4.18484 -0.80887

O -10.39353 -1.95031 -0.63300

H -10.60485 -2.89656 -0.67956

H -5.04758 2.57473 -1.74515

H -4.41262 0.17015 -1.58462

C 6.01593 0.70968 0.56072

C 7.85595 -0.64343 1.51974

C 6.83007 -1.12995 -0.69262

C 7.49010 -1.74568 0.53742

O 5.68613 -0.38197 -0.26177

C 6.64356 0.20595 1.85228

H 8.61585 -0.00064 1.05080

H 7.53908 -0.45123 -1.18907

H 6.77046 -2.42439 1.01804

H 5.89276 -0.41097 2.36335

H 6.69890 1.39388 0.03211

O 7.08419 1.26352 2.67361

H 6.31961 1.77849 2.94964

O 8.38928 -1.26342 2.67067

H 8.61074 -0.57509 3.30652

O 8.64092 -2.44238 0.12111

H 9.12185 -2.70379 0.91387

C 6.36060 -2.16275 -1.69889

H 5.55817 -2.75333 -1.24191

H 7.20299 -2.81910 -1.92380

O 5.93270 -1.54474 -2.89471

H 4.96952 -1.49189 -2.87000

astringin-astringin 8-5 SS

100

Energy: -1821859.7799604

O 7.28898 -0.57831 0.85101

C 6.84393 0.71369 1.02001

C 6.09204 3.35794 1.41679

C 7.76348 1.60777 1.54843

C 5.55500 1.11583 0.69375

C 5.17551 2.44865 0.88511

C 7.37429 2.92956 1.74465

H 8.76378 1.28819 1.80848

H 4.82746 0.40349 0.32906

H 5.80577 4.38929 1.59431

O 8.30906 3.76780 2.26898

H 7.94024 4.65049 2.35878

C 3.79687 2.79504 0.52761

H 3.19855 1.93737 0.23243

C 3.20893 3.99796 0.49006

H 3.77172 4.90609 0.69074

C 1.79282 4.14167 0.11253

C -0.77995 4.24735 -0.88967

C 1.34373 5.27228 -0.58177

C 0.90351 3.08640 0.36607

C -0.36541 3.14228 -0.16821

C 0.04772 5.34233 -1.09078

H 2.01577 6.09849 -0.78530

H 1.21330 2.23155 0.95354

O -0.35955 6.43579 -1.78591

H -1.25345 6.27900 -2.11045

C -4.15957 -2.23924 0.39462

C -6.37478 -3.36779 0.36365

C -6.00209 -1.11227 1.30555

C -6.80149 -2.40535 1.45495

O -4.62426 -1.42259 1.45293

C -4.87510 -3.58577 0.43767

H -6.61755 -2.92513 -0.61381

H -6.19002 -0.69541 0.30674

H -6.57378 -2.85029 2.43337

H -4.62216 -4.06412 1.39300

H -4.32993 -1.72682 -0.56613

O -2.80479 -2.51158 0.56318

O -4.52004 -4.40583 -0.65204

H -3.56871 -4.54960 -0.62164

O -7.09867 -4.56630 0.55007

H -6.79685 -5.20233 -0.10707

O -8.18493 -2.11029 1.36340

H -8.65773 -2.94611 1.27639

C -6.34844 -0.05696 2.34693

H -5.65064 0.77571 2.24678

H -6.24880 -0.47844 3.35315

O -7.65596 0.46638 2.14620

H -8.26932 -0.28205 2.13941

C -1.90096 -1.55749 0.14268

C -0.02055 0.25181 -0.78190

C -0.68971 -2.03296 -0.35063

C -2.16950 -0.19876 0.22523

C -1.21425 0.70896 -0.24003

C 0.25161 -1.11758 -0.81447

H -0.49353 -3.09863 -0.37415

H -3.09307 0.15299 0.66640

H 0.73105 0.93459 -1.15727

O 1.44918 -1.49946 -1.31491

H 1.63755 -2.43008 -1.09414

C -1.52381 2.18127 -0.12883

C -2.37114 2.72720 -1.31594

C -3.83894 2.47172 -1.15719

C -6.47933 1.75387 -0.64782

C -4.40974 1.39836 -1.83860

C -4.59398 3.19183 -0.23315

C -5.91673 2.83765 0.01325

C -5.72425 1.03613 -1.58113

H -3.83548 0.81713 -2.55183

H -4.14725 4.03101 0.28704

H -6.52351 3.39059 0.72163

O -6.27391 -0.03944 -2.20361

H -7.20226 -0.07772 -1.93355

H -2.09488 2.34482 0.79309

C 6.57731 -1.41665 0.00100

C 6.75710 -3.55985 -1.22643

C 4.65518 -2.76313 -0.15692

C 5.49214 -3.99246 -0.51241

O 5.43512 -1.91131 0.66929

C 7.51025 -2.56612 -0.36222

H 6.48356 -3.06897 -2.17207

H 4.36119 -2.24000 -1.08033

H 5.77202 -4.50266 0.41961

H 7.82562 -3.05239 0.56988

H 6.27224 -0.86951 -0.90516

O 8.61939 -2.12381 -1.11019

H 9.11432 -1.49451 -0.57560

O 7.51120 -4.72508 -1.48494

H 8.34381 -4.45994 -1.88989

O 4.71536 -4.85482 -1.32515

H 5.29808 -5.54136 -1.66971

C 3.39192 -3.12391 0.61511

H 2.92570 -2.20785 0.98031

H 3.64652 -3.75322 1.47440

O 2.44256 -3.77120 -0.22226

H 2.89444 -4.48464 -0.69431

H -1.99798 2.27552 -2.24256

O -2.05797 4.14106 -1.37207

O -7.76706 1.32850 -0.46165

H -7.90749 1.17200 0.49248

astringin-astringin 8-5 RS

100

Energy: -1821855.2713049

O 5.99889 0.85930 -1.01703

C 5.12176 1.86789 -0.67599

C 3.49461 4.03023 -0.06944

C 5.65475 3.15299 -0.70710

C 3.78742 1.64674 -0.36001

C 2.96328 2.73888 -0.05521

C 4.82607 4.23235 -0.40881

H 6.69681 3.28683 -0.97805

H 3.39106 0.63995 -0.36822

H 2.89752 4.89145 0.20044

O 5.28019 5.51364 -0.41205

H 6.20251 5.53672 -0.68052

C 1.55020 2.49322 0.27355

H 1.31400 1.48856 0.61553

C 0.57644 3.40366 0.14807

H 0.82945 4.37333 -0.27336

C -0.83889 3.23857 0.49894

C -3.52322 3.06083 1.09862

C -1.74622 4.19193 0.01607

C -1.29941 2.18469 1.30711

C -2.65105 2.10803 1.59352

C -3.10321 4.11525 0.30324

H -1.40193 5.00634 -0.61117

H -0.61030 1.45701 1.72012

O -3.97203 5.03387 -0.18778

H -4.86262 4.80422 0.10171

C -0.95606 -2.06885 -0.71175

C 0.77841 -2.24968 -2.47584

C -0.99515 -0.49278 -2.46387

C -0.10844 -1.36543 -3.34116

O -1.76283 -1.32451 -1.59316

C -0.07259 -3.03572 -1.49063

H 1.46073 -1.60018 -1.90259

H -0.37134 0.19179 -1.86744

H -0.75158 -2.01927 -3.94859

H -0.72252 -3.72681 -2.04150

H -0.33540 -1.38642 -0.10696

O -1.76474 -2.83652 0.12386

O 0.79232 -3.72790 -0.61840

H 0.25410 -4.27360 -0.03669

O 1.50760 -3.07800 -3.34403

H 2.28472 -3.40625 -2.86190

O 0.66681 -0.52585 -4.16256

H 1.32284 -1.08795 -4.59097

C -2.00293 0.30760 -3.25994

H -2.59874 -0.38475 -3.86830

H -1.48468 1.00868 -3.91267

O -2.84759 1.05104 -2.39268

H -2.99178 0.51000 -1.60299

C -2.36119 -2.21630 1.19286

C -3.53612 -1.11545 3.45085

C -2.77996 -3.05536 2.22138

C -2.54418 -0.84220 1.26641

C -3.14214 -0.29560 2.40216

C -3.36707 -2.49528 3.34851

H -2.62800 -4.12506 2.13011

H -2.26012 -0.20085 0.44506

H -4.00022 -0.70949 4.34253

O -3.79250 -3.25060 4.39635

H -3.64689 -4.18218 4.21090

C -3.47100 1.17475 2.45724

C -4.92726 1.51649 1.95436

C -5.44129 0.59742 0.87252

C -6.25591 -1.21225 -1.09834

C -5.22737 0.88605 -0.47644

C -6.08582 -0.58629 1.21858

C -6.49004 -1.48909 0.23983

C -5.62538 -0.01440 -1.45418

H -4.76709 1.81937 -0.78363

H -6.26668 -0.81783 2.26247

H -6.98803 -2.41528 0.49860

O -5.45625 0.19715 -2.79302

H -4.67174 0.75714 -2.91931

H -3.39555 1.50987 3.49778

C 6.06192 -0.29328 -0.23629

C 6.21559 -1.35576 1.98971

C 5.07322 -2.42633 0.05779

C 5.08886 -2.28854 1.57566

O 4.96931 -1.13493 -0.54231

C 6.08182 -0.02486 1.27049

H 7.17526 -1.81560 1.70953

H 6.00361 -2.90924 -0.27471

H 4.13688 -1.84390 1.90236

H 5.14018 0.44683 1.57286

H 6.99895 -0.77926 -0.53493

O 7.18830 0.76808 1.64451

H 7.03130 1.67464 1.35966

O 6.14123 -1.20659 3.39087

H 6.80901 -0.56573 3.65734

O 5.25609 -3.57261 2.12776

H 5.41362 -3.46259 3.07197

C 3.87602 -3.20128 -0.45070

H 2.95663 -2.75668 -0.05597

H 3.92924 -4.23794 -0.12186

O 3.86013 -3.19114 -1.87119

H 4.12951 -2.30538 -2.14611

H -5.62585 1.53516 2.79051

O -4.82544 2.87031 1.45647

O -6.64215 -2.08673 -2.05824

H -6.36652 -1.72665 -2.91058

isorhapontin-isorhapontin 8-5 RS quinone methide

106

Energy: -1871120.1640580

C -5.44540 1.45276 -0.66582

C -7.48875 0.13741 -0.15064

C -5.73222 -0.62818 -1.70663

C -7.22818 -0.65723 -1.41786

O -5.31445 0.72525 -1.86764

C -6.91162 1.53839 -0.26416

H -6.98736 -0.37994 0.67994

H -5.19684 -1.07557 -0.85492

H -7.76394 -0.18254 -2.25261

H -7.44972 2.09997 -1.03855

H -4.87005 0.96136 0.12982

O -4.98784 2.75345 -0.85667

O -7.05973 2.13942 1.00504

H -6.68514 3.02564 0.96985

O -8.88579 0.15754 0.05792

H -9.06253 0.67430 0.85086

O -7.62265 -2.00168 -1.26846

H -8.51477 -2.00215 -0.90441

C -5.36007 -1.35405 -2.98110

H -5.89284 -0.90778 -3.82762

H -5.64130 -2.40323 -2.90074

O -3.95604 -1.30297 -3.19444

H -3.71773 -0.37609 -3.31695

C -3.62985 2.96673 -0.80396

C -0.91866 3.54453 -0.61429

C -3.22831 4.27951 -0.60496

C -2.69872 1.94145 -0.93356

C -1.33300 2.22853 -0.83278

C -1.86785 4.55722 -0.51431

H -3.95634 5.07352 -0.50519

H -3.00504 0.92181 -1.12967

H 0.13488 3.78186 -0.50962

O -1.52406 5.85667 -0.30310

H -0.56725 5.93868 -0.27043

C -0.40805 1.09453 -0.96787

H -0.88953 0.12655 -0.87304

C 0.90759 1.16674 -1.20892

H 1.39034 2.13471 -1.32223

C 1.79890 0.01092 -1.34368

C 3.45086 -2.29165 -1.84915

C 1.23574 -1.34557 -1.31889

C 3.12893 0.19254 -1.44637

C 4.08024 -0.95611 -1.48535

C 1.99519 -2.43093 -1.56338

H 0.18478 -1.46311 -1.08438

H 3.54777 1.19450 -1.44429

O 4.12936 -3.21265 -2.23694

O 1.58536 -3.71059 -1.54795

C 0.25233 -3.93120 -1.12159

H -0.46166 -3.47613 -1.81621

H 0.10310 -3.50730 -0.12223

H 0.11616 -5.00991 -1.09895

C -0.56609 -0.83142 2.65130

C -2.80226 0.14743 3.04509

C -2.40758 -1.76245 1.49508

C -3.24185 -0.51031 1.75341

O -1.03027 -1.40083 1.45099

C -1.32251 0.45425 2.96422

H -2.97996 -0.54046 3.88599

H -2.58541 -2.48659 2.30500

H -3.05169 0.19340 0.93519

H -1.13814 1.15552 2.13852

H -0.67108 -1.55189 3.47951

O 0.76544 -0.44878 2.49280

O -0.93456 1.00187 4.20425

H -0.00316 1.23896 4.15208

O -3.58350 1.31356 3.20173

H -3.24479 1.80307 3.95885

O -4.61038 -0.86483 1.80357

H -5.07279 -0.13643 2.23907

C -2.75103 -2.42062 0.17302

H -3.81939 -2.66325 0.18668

H -2.18281 -3.35290 0.08099

O -2.42535 -1.52641 -0.87017

H -2.94624 -1.70796 -1.66921

C 1.68651 -1.38112 2.08365

C 3.59447 -3.09281 1.04478

C 1.49277 -2.74921 2.19802

C 2.82442 -0.85411 1.48145

C 3.75548 -1.71163 0.91085

C 2.46716 -3.59931 1.67965

H 0.59718 -3.17989 2.62474

H 2.87827 0.21959 1.36120

H 4.29883 -3.77105 0.57291

O 2.22739 -4.93440 1.78143

H 2.87749 -5.41786 1.26299

C 4.78954 -1.19845 -0.07192

C 5.50550 0.04035 0.37086

H 4.99370 0.67145 1.09236

C 6.71541 0.45266 -0.06657

C 9.23288 1.46171 -1.04543

C 7.28279 1.69502 0.43783

C 7.50059 -0.29466 -1.04532

C 8.67490 0.17095 -1.49404

C 8.46047 2.18067 0.00634

H 6.72798 2.25842 1.18060

H 7.11913 -1.23993 -1.41553

H 9.26565 -0.36296 -2.22901

O 10.25598 1.91534 -1.52150

O 8.89420 3.38011 0.45656

C 10.26559 3.48671 0.84967

H 10.90603 3.66102 -0.01174

H 10.58621 2.57962 1.36884

H 10.30841 4.32968 1.53716

H 4.89037 -0.77006 -2.19557

H 5.50868 -1.99920 -0.25976

isorhapontin-isorhapontin 8-5 SS quinone methide

106

Energy: -1871119.5414431

C 6.71320 0.52191 -0.54006

C 8.87791 0.70947 0.62448

C 6.73979 0.14785 1.77468

C 8.10244 0.82422 1.92569

O 6.04099 0.72457 0.67754

C 8.05195 1.24407 -0.52914

H 9.08974 -0.35400 0.43755

H 6.88943 -0.92781 1.59634

H 7.94619 1.89140 2.14185

H 7.85989 2.31499 -0.38484

H 6.86524 -0.55581 -0.70265

O 5.95574 1.07560 -1.57172

O 8.78298 1.00818 -1.71215

H 8.27807 1.35711 -2.45384

O 10.08644 1.42099 0.78977

H 10.56891 1.38689 -0.04287

O 8.79298 0.19709 2.98199

H 9.69953 0.52312 2.96722

C 5.88622 0.32498 3.01240

H 5.69374 1.39260 3.16263

H 6.44111 -0.05374 3.87132

O 4.67436 -0.39699 2.91866

H 3.95860 0.15429 2.57489

C 4.82303 0.37356 -1.93802

C 2.52254 -0.93546 -2.74179

C 4.91935 -0.92320 -2.42081

C 3.60040 1.01921 -1.83228

C 2.43385 0.37023 -2.24746

C 3.75121 -1.58274 -2.79667

H 5.86885 -1.43733 -2.50177

H 3.57185 2.02083 -1.42159

H 1.64264 -1.47565 -3.07140

O 3.86049 -2.87042 -3.21944

H 3.09017 -3.35406 -2.89282

C 1.15519 1.07257 -2.07952

H 1.23414 2.02369 -1.56204

C -0.04792 0.62764 -2.46724

H -0.12742 -0.31929 -2.99563

C -1.33317 1.27610 -2.20323

C -3.84744 2.61281 -1.68181

C -1.39502 2.58171 -1.54168

C -2.47520 0.63572 -2.50999

C -3.82192 1.16124 -2.14192

C -2.55037 3.21235 -1.27040

H -0.48109 3.09959 -1.27654

H -2.43948 -0.34767 -2.97187

O -4.88665 3.23502 -1.63583

O -2.54111 4.46730 -0.76183

C -3.23497 4.64408 0.47375

H -2.76513 4.04014 1.25558

H -4.28841 4.37988 0.37119

H -3.14223 5.69994 0.71829

C 0.12730 -0.92325 1.14807

C 2.41405 -1.89425 0.79177

C 0.33706 -2.65035 -0.39676

C 1.68763 -3.07700 0.15273

O -0.42528 -2.13245 0.69015

C 1.49331 -1.17850 1.77776

H 2.69629 -1.17987 0.00392

H 0.46874 -1.85994 -1.14918

H 1.51865 -3.82899 0.93566

H 1.34735 -1.81116 2.66182

H 0.21036 -0.21069 0.31171

O -0.68113 -0.38630 2.15057

O 2.07932 0.06433 2.13611

H 1.48836 0.49189 2.76592

O 3.55870 -2.45452 1.39205

H 4.08087 -1.77889 1.85829

O 2.46510 -3.63438 -0.89097

H 3.35976 -3.73786 -0.54011

C -0.47942 -3.77592 -0.99769

H -0.57729 -4.58684 -0.26601

H 0.02473 -4.16647 -1.88141

O -1.74721 -3.29867 -1.40494

H -2.20047 -3.00452 -0.60807

C -1.87550 0.19023 1.79197

C -4.31496 1.39981 1.25850

C -2.41995 1.06386 2.72463

C -2.53008 -0.08513 0.59234

C -3.75451 0.52953 0.32908

C -3.64106 1.66390 2.44918

H -1.90871 1.26442 3.65697

H -2.10259 -0.77027 -0.12613

H -5.27360 1.86903 1.06001

O -4.13100 2.52646 3.37898

H -5.00457 2.82869 3.11429

C -4.44588 0.26526 -1.00473

C -5.94494 0.43985 -0.95944

H -6.35230 1.28504 -1.50674

C -6.79643 -0.36149 -0.28713

C -8.64408 -2.02522 1.17536

C -8.23129 -0.09927 -0.32656

C -6.33126 -1.49103 0.51432

C -7.18769 -2.26370 1.19536

C -9.10930 -0.86684 0.34983

H -8.56073 0.74048 -0.92626

H -5.26569 -1.68665 0.56504

H -6.85954 -3.10121 1.79938

O -9.42007 -2.72142 1.78996

O -10.44075 -0.71634 0.38239

C -10.98771 0.34759 -0.36835

H -10.75763 0.23261 -1.43233

H -10.60628 1.31098 -0.01505

H -12.06329 0.30168 -0.21850

H -4.50112 1.08564 -2.99727

H -4.22493 -0.76910 -1.29070

isorhapontin-isorhapontin 8-5 RS

106

Energy: -1871164.2047182

C 5.53321 0.94813 0.26258

C 5.26786 3.07788 1.44532

C 3.67766 2.29524 -0.32443

C 4.28003 3.51007 0.37430

O 4.70959 1.42330 -0.77321

C 6.29442 2.11124 0.87765

H 4.71238 2.55811 2.24043

H 3.01134 1.76837 0.37515

H 4.81525 4.12720 -0.36064

H 6.88205 2.59990 0.09026

H 4.92971 0.45806 1.04183

O 6.44994 0.05729 -0.28146

O 7.10924 1.70202 1.95250

H 7.76663 1.08262 1.61977

O 5.86200 4.25050 1.95762

H 6.51852 3.99117 2.61288

O 3.21003 4.23722 0.94543

H 3.57949 4.96579 1.45692

C 2.90139 2.67264 -1.57003

H 3.58194 3.15625 -2.27985

H 2.10164 3.36796 -1.31277

O 2.30263 1.52880 -2.15561

H 2.99891 0.88227 -2.32055

C 5.96888 -1.15684 -0.71976

C 5.15977 -3.63724 -1.67301

C 6.87663 -1.94916 -1.40819

C 4.66276 -1.58381 -0.50037

C 4.26398 -2.84339 -0.95717

C 6.46021 -3.18607 -1.88755

H 7.88627 -1.60407 -1.58660

H 3.92477 -0.95051 -0.02993

H 4.85078 -4.61193 -2.03762

O 7.38234 -3.92213 -2.56393

H 6.98967 -4.75068 -2.85141

C 2.89678 -3.31130 -0.66566

H 2.37442 -3.85940 -1.44591

C 2.30628 -3.09992 0.51670

H 2.89707 -2.64864 1.31186

C 0.91332 -3.41882 0.85582

C -1.71080 -3.88272 1.60093

C 0.59180 -3.74422 2.18558

C -0.09922 -3.32619 -0.10114

C -1.40724 -3.55442 0.29463

C -0.72030 -3.99325 2.57961

H 1.39281 -3.78914 2.91289

H 0.12623 -3.01115 -1.11354

O -1.12891 -4.29547 3.83382

C -0.13731 -4.37194 4.83711

H 0.37496 -3.41234 4.95885

H 0.59583 -5.15139 4.60747

H -0.65981 -4.62389 5.75674

C -1.89347 1.82399 -0.76602

C -0.71881 3.63838 0.53252

C -3.16542 3.59387 0.15573

C -1.94565 4.49498 0.27839

O -2.98006 2.70646 -0.94646

C -0.58123 2.57594 -0.54376

H -0.83674 3.14084 1.50651

H -3.29295 3.01876 1.08349

H -1.80527 5.03195 -0.67153

H -0.32375 3.06594 -1.49256

H -2.10098 1.14923 0.07745

O -1.73583 1.11439 -1.96020

O 0.42924 1.67875 -0.14067

H 0.85078 1.30533 -0.92925

O 0.38541 4.51851 0.55546

H 1.20062 4.05957 0.80083

O -2.15673 5.40311 1.33589

H -1.30670 5.81908 1.51782

C -4.43341 4.37160 -0.13067

H -4.30347 4.93994 -1.05932

H -4.62519 5.06583 0.68636

O -5.55431 3.50900 -0.22821

H -5.34363 2.81148 -0.86145

C -2.39113 -0.08142 -2.11306

C -3.59924 -2.52378 -2.60609

C -3.18511 -0.22967 -3.24583

C -2.19780 -1.12760 -1.22362

C -2.81902 -2.35263 -1.46702

C -3.78235 -1.46137 -3.48877

H -3.30755 0.61242 -3.91815

H -1.57166 -0.98802 -0.35005

H -4.07976 -3.47148 -2.82174

O -4.57469 -1.68250 -4.57546

H -4.57840 -0.90420 -5.13929

C -2.71002 -3.47523 -0.46575

C -3.72811 -3.39710 0.73674

C -4.13758 -1.99094 1.11564

C -4.87354 0.65542 1.68321

C -5.04085 -1.31404 0.28707

C -3.61003 -1.33886 2.21890

C -3.98458 -0.02362 2.50063

C -5.38628 0.00072 0.54504

H -5.44621 -1.82026 -0.58022

H -2.92538 -1.85587 2.87974

H -3.60314 0.49240 3.37363

O -6.22117 0.75188 -0.23085

C -6.77292 0.13457 -1.37823

H -7.38844 0.89133 -1.86037

H -7.39782 -0.71917 -1.10008

H -5.98285 -0.19595 -2.06074

H -2.87880 -4.42552 -0.98385

O -3.04134 -4.02700 1.83482

O -5.25671 1.90737 2.01416

H -5.65789 2.36534 1.25164

H -4.61231 -4.00020 0.52766

isorhapontin-isorhapontin 8-5 SS

106

Energy: -1871162.2426969

C -5.30648 -1.04139 -1.20801

C -6.19105 -3.32543 -0.80559

C -3.87346 -2.62598 -0.27781

C -4.74822 -3.78865 -0.73490

O -3.99835 -1.56797 -1.21577

C -6.29226 -2.09657 -1.69367

H -6.52275 -3.05261 0.20722

H -4.21350 -2.28785 0.70838

H -4.42815 -4.10976 -1.73544

H -6.01460 -2.36313 -2.72176

H -5.55791 -0.70893 -0.18690

O -5.38514 0.03312 -2.09235

O -7.62573 -1.64298 -1.62585

H -7.71076 -0.87399 -2.19900

O -6.96241 -4.40254 -1.29434

H -7.87060 -4.09828 -1.39506

O -4.58657 -4.84695 0.19435

H -5.24385 -5.52264 -0.00566

C -2.40342 -3.00343 -0.15062

H -1.80541 -2.09106 -0.13216

H -2.08350 -3.60973 -1.00594

O -2.16275 -3.68488 1.07351

H -2.79151 -4.41765 1.12136

C -4.66304 1.15097 -1.69603

C -3.18777 3.34155 -0.88410

C -5.26748 2.09831 -0.88530

C -3.34740 1.27474 -2.11428

C -2.59058 2.37544 -1.70313

C -4.50910 3.19854 -0.48060

H -6.29262 1.98702 -0.55433

H -2.91385 0.48918 -2.71996

H -2.60781 4.17902 -0.50927

O -5.10873 4.10108 0.34127

H -4.43184 4.69159 0.68818

C -1.15690 2.37172 -2.00941

H -0.82053 1.48925 -2.54653

C -0.24150 3.24402 -1.56311

H -0.54824 4.15564 -1.05577

C 1.20043 2.96135 -1.52326

C 3.85167 2.36884 -0.90705

C 2.12476 3.97608 -1.25581

C 1.64263 1.63067 -1.53768

C 2.95111 1.35814 -1.21598

C 3.46620 3.70896 -0.96407

H 1.80525 5.01176 -1.21404

H 0.94493 0.81322 -1.66587

O 4.26672 4.76246 -0.66756

C 5.65743 4.62770 -0.94280

H 5.80940 4.18756 -1.93240

H 6.15267 4.01165 -0.19282

H 6.06242 5.63750 -0.92554

C -0.87488 0.05003 1.76649

C -3.00648 1.18551 2.32033

C -0.93290 2.39592 1.73872

C -2.19221 2.43311 2.60493

O -0.18974 1.23568 2.10563

C -2.16093 -0.04839 2.57992

H -3.30613 1.19131 1.25942

H -1.22475 2.33337 0.68336

H -1.88985 2.43038 3.66110

H -1.87763 -0.08606 3.64006

H -1.08964 0.04965 0.68365

O -0.11041 -1.06803 2.10385

O -2.93898 -1.16273 2.21928

H -2.41477 -1.97767 2.26695

O -4.15089 1.23144 3.14885

H -4.64389 0.41431 3.02226

O -2.95518 3.60117 2.33090

H -3.82876 3.45559 2.71726

C -0.02286 3.60273 1.91050

H 0.90310 3.41981 1.36076

H 0.22451 3.73536 2.97106

O -0.60900 4.76903 1.36370

H -1.45425 4.90209 1.80749

C 0.89047 -1.44903 1.23611

C 2.83559 -2.33043 -0.53085

C 0.99407 -2.81110 0.96978

C 1.75480 -0.53012 0.66114

C 2.71639 -0.97380 -0.25294

C 1.98490 -3.24242 0.09602

H 0.27713 -3.49397 1.41381

H 1.65667 0.51853 0.90505

H 3.57436 -2.69657 -1.23495

O 2.15465 -4.55994 -0.20834

H 1.49030 -5.08420 0.24783

C 3.61656 0.03281 -0.93528

C 4.81148 0.51889 -0.05970

C 6.04729 -0.31996 -0.19618

C 8.21603 -2.03967 -0.53727

C 6.05681 -1.56669 0.44212

C 7.12054 0.05835 -0.98767

C 8.20883 -0.80046 -1.14979

C 7.12608 -2.42614 0.26072

H 5.21793 -1.85397 1.06459

H 7.11152 1.02533 -1.47383

H 9.06090 -0.52139 -1.75724

O 7.24175 -3.67246 0.80385

C 6.12503 -4.17711 1.51462

H 5.23283 -4.18711 0.88087

H 5.93278 -3.58019 2.41063

H 6.38300 -5.19319 1.80326

H 4.01410 -0.40270 -1.85836

O 5.05293 1.88250 -0.46210

O 9.26718 -2.88023 -0.70201

H 9.08012 -3.69069 -0.21328

H 4.47728 0.54627 0.98553

astringin-astringin 8-12 RS quinone methide

100

Energy: -1821808.4019073

C 1.27340 -0.97765 1.90460

C 3.65323 -1.87368 2.08087

C 2.70821 -0.11669 3.55139

C 3.94731 -0.56844 2.79018

O 1.70182 0.17430 2.59043

C 2.40986 -1.73422 1.21319

H 3.47335 -2.65950 2.82811

H 2.36015 -0.92741 4.20942

H 4.17795 0.19175 2.03203

H 2.67389 -1.14032 0.32214

H 0.73467 -1.64097 2.59877

O 0.41904 -0.59582 0.85550

O 1.98768 -3.03164 0.87329

H 1.34613 -2.96779 0.14325

O 4.80115 -2.17344 1.31222

H 4.65836 -2.94647 0.74928

O 5.01865 -0.68666 3.70741

H 5.73556 -1.14512 3.25363

C 2.96816 1.11454 4.41429

H 3.29738 0.78930 5.40383

H 2.03753 1.67288 4.52257

O 3.92918 2.00097 3.86215

H 4.78762 1.56370 3.93859

C -0.73122 0.09411 1.15748

C -3.04008 1.58786 1.69992

C -0.57169 1.57824 1.29464

C -1.91166 -0.53041 1.09673

C -3.16002 0.19633 1.29832

C -1.84940 2.20604 1.74947

H -1.90980 -1.59171 0.88459

H -3.94153 2.11626 1.99562

O -1.65149 3.46711 2.19609

H -2.49378 3.89690 2.38099

C -4.39702 -0.36922 1.16149

H -5.24624 0.27644 1.35597

C -4.65037 -1.72092 0.78014

H -3.78830 -2.34806 0.57439

C -5.87100 -2.32613 0.63204

C -8.39276 -3.64666 0.30611

C -5.92829 -3.71475 0.22755

C -7.12587 -1.63299 0.86336

C -8.31579 -2.24633 0.71242

C -7.10879 -4.34444 0.07178

H -5.00431 -4.25335 0.04842

H -7.10291 -0.59363 1.16612

H -9.25601 -1.73634 0.88266

O -9.43738 -4.26566 0.15079

O -7.21650 -5.62972 -0.30072

H -8.16807 -5.82144 -0.33489

C 3.74391 -0.61370 -3.06101

C 4.68978 -2.78375 -2.30684

C 2.30748 -2.51134 -3.04757

C 3.27155 -3.29771 -2.16401

O 2.45058 -1.11397 -2.82876

C 4.74083 -1.27972 -2.11537

H 5.06595 -3.02543 -3.31107

H 2.50904 -2.75681 -4.10029

H 2.97891 -3.17388 -1.11666

H 4.47513 -1.03938 -1.07765

H 4.04544 -0.78354 -4.10208

O 3.72638 0.78119 -2.91597

O 6.06626 -0.88336 -2.39983

H 6.12976 0.07360 -2.32335

O 5.46243 -3.45809 -1.32465

H 6.35630 -3.09806 -1.34881

O 3.17079 -4.65196 -2.54833

H 3.78689 -5.15782 -2.00866

C 0.85491 -2.82283 -2.72503

H 0.69604 -3.89966 -2.77316

H 0.20760 -2.32881 -3.45539

O 0.51644 -2.40693 -1.40988

H 0.66318 -1.45409 -1.32975

C 3.24459 1.28838 -1.71912

C 2.31421 2.43165 0.62547

C 4.15459 1.74093 -0.77850

C 1.87232 1.39849 -1.50729

C 1.40716 1.96617 -0.32431

C 3.68386 2.31184 0.40384

H 5.22288 1.67037 -0.94276

H 1.19108 1.05396 -2.27514

H 1.97308 2.88371 1.55168

O 4.60245 2.73416 1.30176

H 4.20306 2.76060 2.18763

C -0.09110 2.13074 -0.11961

C -0.47780 3.56110 -0.35232

C -1.59914 3.99524 -0.97242

C -4.03290 4.97157 -2.13128

C -1.83970 5.42571 -1.09696

C -2.62651 3.08558 -1.47300

C -3.77205 3.53728 -2.01296

C -2.97932 5.89133 -1.63576

H -1.08615 6.11863 -0.73998

H -2.46967 2.01668 -1.38820

H -4.54206 2.86985 -2.38032

O -5.05313 5.44744 -2.60582

O -3.25778 7.19853 -1.76409

H -4.13074 7.25116 -2.18596

H 0.20283 1.80673 2.02823

H 0.21072 4.30783 0.03530

H -0.59050 1.50825 -0.86342

astringin-astringin 8-12 SS quinone methide

100

Energy: -1821812.2508872

C -0.99738 -0.26397 -1.90952

C -1.88571 2.03914 -2.24335

C -3.30033 0.05912 -1.89686

C -3.20082 1.39243 -2.63341

O -2.23175 -0.76459 -2.36238

C -0.73082 1.09794 -2.54615

H -1.93081 2.21681 -1.16155

H -3.19003 0.24314 -0.81707

H -3.21091 1.19848 -3.71521

H -0.65061 0.95630 -3.63016

H -1.01803 -0.20196 -0.81029

O 0.05514 -1.10308 -2.29701

O 0.44516 1.69752 -2.02451

H 1.21174 1.32165 -2.46753

O -1.77293 3.26220 -2.94019

H -0.95132 3.69271 -2.67892

O -4.29771 2.20493 -2.26988

H -4.12071 3.10048 -2.57810

C -4.62358 -0.66125 -2.10665

H -4.51263 -1.69521 -1.77248

H -4.88867 -0.66598 -3.17155

O -5.63635 -0.07443 -1.31852

H -5.64113 0.87187 -1.50911

C 0.21478 -2.29138 -1.63382

C 0.78499 -4.81300 -0.54737

C 1.66404 -2.68670 -1.55059

C -0.78728 -2.98184 -1.08184

C -0.54533 -4.24687 -0.40092

C 1.78988 -4.10976 -1.08802

H -1.80288 -2.63030 -1.18479

H 0.95477 -5.82893 -0.20218

O 3.03722 -4.61320 -1.28332

H 3.05946 -5.54154 -1.02780

C -1.52266 -4.95131 0.24094

H -1.28559 -5.97337 0.52553

C -2.87952 -4.53219 0.48716

H -3.64064 -5.30187 0.36677

C -3.31627 -3.29552 0.87743

C -4.22690 -0.67410 1.56424

C -4.73201 -2.98390 0.89119

C -2.39107 -2.27042 1.32286

C -2.81784 -1.03382 1.64439

C -5.17469 -1.74413 1.19143

H -5.44693 -3.74833 0.60684

H -1.34278 -2.52754 1.42948

H -2.14619 -0.27132 2.01593

O -4.64415 0.45473 1.82444

O -6.47461 -1.42333 1.20573

H -6.52417 -0.45557 1.21276

C -0.39848 2.12978 1.38614

C -1.46643 4.31336 0.83044

C 0.95285 3.91472 0.65452

C -0.10244 4.95911 0.98745

O 0.80485 2.82941 1.56618

C -1.62058 3.01560 1.62134

H -1.58327 4.06742 -0.23570

H 0.80380 3.54985 -0.37236

H 0.03117 5.29021 2.02681

H -1.70209 3.23959 2.69112

H -0.45156 1.73703 0.36249

O -0.40796 1.08970 2.31723

O -2.78810 2.41634 1.10314

H -3.25855 1.82199 1.71059

O -2.44564 5.25965 1.20044

H -3.30839 4.85060 1.06952

O 0.04152 6.03806 0.08958

H -0.72604 6.60972 0.20144

C 2.38103 4.39434 0.80124

H 2.53458 4.77644 1.81783

H 2.57298 5.19732 0.09086

O 3.27987 3.34038 0.51437

H 3.08675 2.62741 1.13425

C 0.38060 -0.00339 2.06378

C 1.78326 -2.37527 1.65906

C 0.39024 -0.97383 3.06143

C 1.08337 -0.19567 0.87812

C 1.75844 -1.40108 0.66972

C 1.10090 -2.15063 2.85443

H -0.16615 -0.79662 3.97535

H 1.10730 0.56543 0.10849

H 2.31767 -3.30814 1.53264

O 1.14959 -3.13793 3.78633

H 0.64126 -2.88509 4.56207

C 2.42679 -1.61469 -0.67985

C 3.90167 -1.87645 -0.53687

C 4.86454 -0.92925 -0.47703

C 6.94929 1.02957 -0.29244

C 6.25526 -1.33657 -0.32575

C 4.57677 0.50389 -0.52399

C 5.54631 1.42902 -0.42961

C 7.23813 -0.42569 -0.24285

H 6.49122 -2.39391 -0.28605

H 3.54767 0.83613 -0.60880

H 5.32407 2.48906 -0.43529

O 7.88119 1.81428 -0.21465

O 8.53573 -0.74488 -0.10981

H 9.01984 0.09597 -0.07350

H 2.10419 -2.62703 -2.55388

H 4.21258 -2.91078 -0.44505

H 2.29273 -0.69050 -1.24332

astringin-astringin 8-12 RS

100

Energy: -1821860.9946619

C -1.83372 3.02155 0.29916

C -1.62946 4.92793 -1.25735

C -1.19688 2.57105 -1.91806

C -0.81210 4.02469 -2.16911

O -0.99032 2.27550 -0.53920

C -1.47971 4.49611 0.19135

H -2.68977 4.84135 -1.53899

H -2.25767 2.41997 -2.16751

H 0.25227 4.15117 -1.92073

H -0.43237 4.60787 0.50192

H -2.88399 2.85525 0.01317

O -1.61333 2.63468 1.62370

O -2.33889 5.30649 0.96321

H -2.26767 5.03430 1.88373

O -1.18163 6.25097 -1.46179

H -1.67647 6.82876 -0.87166

O -1.05436 4.31471 -3.52607

H -0.91540 5.26003 -3.64881

C -0.34279 1.58849 -2.70116

H 0.71318 1.85441 -2.59657

H -0.61389 1.63201 -3.75536

O -0.57187 0.26511 -2.24588

H -0.57090 0.30081 -1.27898

C -1.82367 1.29537 1.89975

C -2.13861 -1.41603 2.53895

C -0.78776 0.60222 2.49824

C -3.02797 0.65585 1.63193

C -3.18364 -0.70562 1.93289

C -0.95957 -0.73945 2.79970

H -3.85884 1.21880 1.22609

H -2.23742 -2.46803 2.77498

O 0.15109 -1.30696 3.32849

C -4.43123 -1.41943 1.62329

H -4.61363 -2.31929 2.20444

C -5.29458 -1.07191 0.66124

H -5.05093 -0.22703 0.02077

C -6.54688 -1.76546 0.33951

C -8.92056 -3.08574 -0.37325

C -7.11872 -1.57705 -0.92602

C -7.20068 -2.60827 1.24298

C -8.37226 -3.26357 0.88911

C -8.28414 -2.23200 -1.27922

H -6.63520 -0.92059 -1.64423

H -6.80463 -2.73891 2.24238

H -8.88678 -3.91396 1.58559

O -8.90571 -2.11029 -2.49432

H -8.38186 -1.56879 -3.08983

C 3.02578 -0.81822 -1.52318

C 3.23314 -3.06699 -2.52243

C 5.11938 -1.88030 -1.46055

C 4.73729 -2.85851 -2.56430

O 4.42309 -0.65172 -1.64004

C 2.50378 -1.73755 -2.61815

H 2.97681 -3.53223 -1.55817

H 4.83831 -2.32123 -0.49552

H 5.01127 -2.43386 -3.54086

H 2.71871 -1.26696 -3.58748

H 2.79270 -1.23455 -0.53287

O 2.43682 0.43212 -1.70115

O 1.13417 -2.00489 -2.46105

H 0.61652 -1.18572 -2.55191

O 2.89122 -3.93329 -3.58423

H 1.93100 -4.01258 -3.59374

O 5.42958 -4.06525 -2.33057

H 5.03000 -4.73850 -2.89241

C 6.59119 -1.52923 -1.43928

H 6.88159 -1.09331 -2.40096

H 7.18426 -2.42493 -1.25756

O 6.86202 -0.61275 -0.38240

H 6.23546 0.11583 -0.47651

C 2.22943 1.20701 -0.58425

C 1.74685 2.87381 1.56394

C 2.62534 2.53580 -0.66105

C 1.56950 0.70566 0.52918

C 1.34980 1.54250 1.61824

C 2.36367 3.37140 0.41654

H 3.12849 2.90903 -1.54377

H 1.22405 -0.32243 0.55081

H 1.55481 3.53217 2.40678

O 2.73598 4.67555 0.29567

H 2.52794 5.15240 1.10370

C 0.61689 1.02774 2.83741

C 1.16208 -0.29455 3.51057

C 2.49564 -0.80879 3.02072

C 4.97880 -1.70996 2.12204

C 2.61065 -2.07361 2.45167

C 3.62376 0.00180 3.13860

C 4.86020 -0.44698 2.68861

C 3.84768 -2.52346 2.00431

H 1.74944 -2.72135 2.35203

H 3.53985 0.99303 3.57155

H 5.74630 0.17060 2.78456

O 3.95087 -3.75272 1.43871

H 4.88347 -3.90735 1.23423

H 0.60778 1.82216 3.58729

O -10.06532 -3.72405 -0.71457

H -10.28784 -3.49021 -1.62340

O 6.16203 -2.24799 1.69409

H 6.61575 -1.60192 1.11964

H 1.22613 -0.11581 4.58767

astringin-astringin 8-12 SS

100

Energy: -1821860.2413682

C -1.96142 2.74649 1.25616

C -1.48878 5.16908 1.46490

C -2.78659 4.19111 -0.41113

C -1.79906 5.28629 -0.01559

O -2.21222 2.92524 -0.11730

C -0.95381 3.77754 1.75063

H -2.41348 5.32110 2.04236

H -3.71827 4.32267 0.16094

H -0.86162 5.13473 -0.56797

H -0.02024 3.61733 1.19408

H -2.90349 2.83003 1.82171

O -1.36688 1.50050 1.44682

O -0.74653 3.68416 3.14238

H -0.32685 2.83842 3.32976

O -0.55072 6.17379 1.78310

H -0.26104 6.03387 2.69100

O -2.37589 6.53235 -0.34253

H -1.83132 7.21604 0.06381

C -3.13201 4.21676 -1.88883

H -3.77417 5.07709 -2.08860

H -3.67113 3.30179 -2.13690

O -1.97552 4.25244 -2.71312

H -1.70272 5.16921 -2.82081

C -2.00894 0.38144 0.96651

C -3.14199 -2.03921 0.08666

C -1.20604 -0.59832 0.41707

C -3.38576 0.18003 1.05024

C -3.95664 -1.01861 0.60445

C -1.78662 -1.79524 0.00263

H -4.02844 0.95319 1.45333

H -3.54583 -2.99253 -0.22621

O -0.86388 -2.68222 -0.46437

C -5.41688 -1.16106 0.69184

H -5.91526 -0.44314 1.33792

C -6.15382 -2.05297 0.01849

H -5.66011 -2.71713 -0.68668

C -7.61115 -2.20281 0.10413

C -10.40387 -2.50581 0.16286

C -8.28061 -2.88318 -0.92215

C -8.36850 -1.69900 1.16606

C -9.74870 -1.84548 1.19274

C -9.65560 -3.02849 -0.89569

H -7.71550 -3.29279 -1.75490

H -7.87574 -1.20690 1.99527

H -10.34015 -1.46391 2.01585

O -10.38697 -3.67240 -1.85921

H -9.81269 -4.00967 -2.55113

C 5.27978 0.48006 -0.33209

C 7.61828 -0.33822 -0.55623

C 5.94976 -1.36420 0.95032

C 7.43014 -1.02839 0.78050

O 5.21389 -0.14985 0.93438

C 6.72143 0.88354 -0.62614

H 7.32767 -1.03341 -1.35780

H 5.63703 -2.00497 0.11521

H 7.73146 -0.34194 1.58368

H 7.02590 1.60453 0.14402

H 4.89132 -0.20507 -1.10280

O 4.54087 1.65927 -0.31584

O 6.86233 1.42891 -1.91779

H 6.29176 2.20175 -1.98100

O 8.98597 -0.00411 -0.67195

H 9.10421 0.48567 -1.49275

O 8.18955 -2.22352 0.84989

H 9.08540 -2.01868 0.55795

C 5.63181 -2.07905 2.25628

H 4.54851 -2.16355 2.35436

H 6.01639 -1.49983 3.10295

O 6.15724 -3.40101 2.26909

H 7.10614 -3.33621 2.09068

C 3.17713 1.56540 -0.51165

C 0.45202 1.47040 -1.00635

C 2.57958 2.60568 -1.20861

C 2.43309 0.50077 -0.01627

C 1.06251 0.46235 -0.27017

C 1.20829 2.55597 -1.44987

H 3.16663 3.44101 -1.56762

H 2.90779 -0.27561 0.56976

H -0.61542 1.44870 -1.19387

O 0.65522 3.59541 -2.11696

H -0.30012 3.47293 -2.24885

C 0.28267 -0.71822 0.24943

C 0.33747 -1.92904 -0.72866

C 1.57608 -2.75860 -0.57071

C 4.00575 -4.04679 -0.14267

C 2.61861 -2.59290 -1.48009

C 1.74622 -3.57981 0.54249

C 2.95848 -4.22968 0.75076

C 3.83182 -3.23143 -1.26534

H 2.51027 -1.94947 -2.34647

H 0.92697 -3.71540 1.23896

H 3.10819 -4.87941 1.60585

O 4.87007 -3.03759 -2.12083

H 5.58924 -3.61423 -1.82659

H 0.72525 -1.02992 1.20353

O -11.75065 -2.65072 0.19428

H -12.02490 -3.13835 -0.59127

O 5.23439 -4.63702 -0.01163

H 5.58856 -4.42165 0.87292

H 0.26277 -1.54383 -1.75237

isorhapontin-isorhapontin 8-12 RS quinone methide

106

Energy: -1871106.4009995

C 2.33953 -1.94674 0.89540

C 3.15946 -4.26374 0.62716

C 4.65091 -2.35339 1.16457

C 4.55437 -3.69612 0.44846

O 3.64041 -1.47049 0.66574

C 2.11329 -3.26566 0.16414

H 2.99873 -4.46350 1.69769

H 4.50174 -2.50299 2.24370

H 4.73105 -3.53861 -0.62638

H 2.23454 -3.07804 -0.91177

H 2.15740 -2.07749 1.97339

O 1.42954 -1.00134 0.37974

O 0.85157 -3.81054 0.44871

H 0.20473 -3.36394 -0.12276

O 3.08795 -5.46880 -0.10346

H 2.17857 -5.78368 -0.05323

O 5.53029 -4.55324 0.99350

H 5.35664 -5.43890 0.65627

C 5.98294 -1.66732 0.93247

H 6.14998 -1.56980 -0.14880

H 6.77764 -2.28325 1.35209

O 6.03044 -0.41168 1.56696

H 5.32179 0.12346 1.19319

C 0.82949 -0.15568 1.28685

C -0.39415 1.57658 3.12578

C 1.64945 0.99407 1.78867

C -0.44773 -0.37561 1.61147

C -1.17393 0.53020 2.49064

C 0.90641 1.75269 2.84756

H -0.93466 -1.23378 1.16385

H -0.88647 2.21462 3.85363

O 1.69055 2.69119 3.43019

H 1.18854 3.17831 4.09183

C -2.52143 0.44588 2.69254

H -2.98524 1.23411 3.27644

C -3.36173 -0.58257 2.15458

H -2.88280 -1.51639 1.87058

C -4.70719 -0.50200 1.91787

C -7.30236 -0.17742 0.74324

C -5.38199 -1.61385 1.28286

C -5.46173 0.72341 2.10381

C -6.68779 0.86976 1.57157

C -6.60301 -1.48427 0.72238

H -4.84836 -2.54833 1.14488

H -5.00497 1.55028 2.63421

H -7.24273 1.79713 1.64670

O -8.29321 0.04234 0.06050

O -7.08592 -2.49820 -0.03892

C -8.48493 -2.79417 0.04077

H -9.05716 -2.13636 -0.60896

H -8.83574 -2.69147 1.07031

H -8.58314 -3.83150 -0.27396

C -2.72030 1.27693 -1.52428

C -4.85471 0.03654 -1.73075

C -2.64182 -1.07649 -1.59115

C -4.05168 -1.17830 -2.16672

O -2.01151 0.14264 -1.97828

C -4.12723 1.30925 -2.11900

H -4.92879 0.03103 -0.63716

H -2.72314 -1.11549 -0.49434

H -4.00070 -1.18624 -3.26583

H -4.03613 1.37659 -3.21059

H -2.76644 1.26993 -0.42189

O -2.07940 2.43532 -1.96896

O -4.86057 2.39130 -1.59302

H -4.44867 3.20688 -1.89374

O -6.13100 -0.08350 -2.31287

H -6.75412 0.49512 -1.85860

O -4.60658 -2.37813 -1.68419

H -5.57155 -2.30267 -1.67051

C -1.75036 -2.20204 -2.06097

H -1.55095 -2.08067 -3.13111

H -2.25460 -3.15311 -1.89499

O -0.53577 -2.23787 -1.32641

H -0.12027 -1.36730 -1.33142

C -1.08783 2.96872 -1.17995

C 0.86194 4.17497 0.35999

C -1.16517 4.33248 -0.93811

C -0.04319 2.19430 -0.68166

C 0.93394 2.80747 0.10274

C -0.17872 4.93465 -0.16885

H -1.97855 4.91983 -1.34347

H -0.00108 1.14193 -0.92950

H 1.62514 4.65128 0.96863

O -0.28939 6.27190 0.04760

H 0.46805 6.58660 0.54833

C 2.10265 2.03424 0.68468

C 2.94753 1.39530 -0.37197

H 2.44638 0.68522 -1.02129

C 4.27388 1.57584 -0.55566

C 7.14474 1.70755 -0.87249

C 4.96168 0.74657 -1.54002

C 5.08539 2.50961 0.22371

C 6.41577 2.57268 0.07472

C 6.30223 0.77315 -1.68080

H 4.34923 0.05940 -2.11054

H 4.60064 3.16650 0.93525

H 7.02950 3.25670 0.64828

O 8.34686 1.75469 -0.99887

O 7.02910 0.00210 -2.50262

C 6.32641 -0.92111 -3.30806

H 5.63147 -0.40515 -3.97804

H 5.77145 -1.63540 -2.68964

H 7.07772 -1.44768 -3.89086

H 2.58367 0.60111 2.21457

H 2.70647 2.74610 1.24352

isorhapontin-isorhapontin 8-12 SS quinone methide

106

Energy: -1871110.0229277

C -2.00795 2.38895 0.74731

C -3.12096 4.46667 -0.01361

C -4.34636 2.56357 0.99443

C -4.43226 3.69892 -0.02014

O -3.24326 1.72062 0.64691

C -1.94122 3.53920 -0.25069

H -2.99964 4.93186 0.97644

H -4.18112 2.98303 1.99749

H -4.58915 3.27032 -1.02106

H -2.01855 3.10341 -1.25726

H -1.84981 2.74897 1.77565

O -0.98034 1.49782 0.39808

O -0.77304 4.30339 -0.10634

H -0.01483 3.79608 -0.44079

O -3.20189 5.46479 -1.00784

H -2.34787 5.90945 -1.04348

O -5.50760 4.53355 0.34269

H -5.45940 5.32207 -0.20878

C -5.58848 1.69663 1.03481

H -5.73710 1.22896 0.05309

H -6.44764 2.33051 1.25303

O -5.50144 0.72547 2.05665

H -4.86679 0.06442 1.76211

C -0.58731 0.54992 1.31472

C 0.28704 -1.57378 2.92637

C -1.59961 -0.49818 1.66963

C 0.69584 0.52665 1.68242

C 1.23725 -0.57783 2.46300

C -1.01185 -1.51624 2.59666

H 1.33656 1.31265 1.30218

H 0.65111 -2.37394 3.56402

O -1.94663 -2.40271 3.01830

H -1.53523 -3.09004 3.55321

C 2.57479 -0.72057 2.69055

H 2.90846 -1.63816 3.16427

C 3.55859 0.24892 2.30169

H 3.21120 1.27034 2.16775

C 4.87990 0.02943 2.03121

C 7.41096 -0.45568 0.78095

C 5.69679 1.13446 1.56697

C 5.46257 -1.29909 2.01938

C 6.65775 -1.52686 1.44782

C 6.89328 0.92760 0.97356

H 5.27097 2.12877 1.62695

H 4.89436 -2.12586 2.42884

H 7.08355 -2.51998 1.37303

O 8.36813 -0.68762 0.06320

O 7.67948 1.86721 0.42534

C 7.28071 3.21692 0.59426

H 7.20381 3.46326 1.65801

H 6.32408 3.40194 0.09775

H 8.06075 3.81867 0.13433

C 2.82921 -1.28902 -1.46316

C 5.11950 -0.35199 -1.70983

C 3.09893 1.06169 -1.45874

C 4.47225 0.96360 -2.11168

O 2.29140 -0.04391 -1.86503

C 4.21425 -1.51182 -2.07227

H 5.23733 -0.34978 -0.62098

H 3.22998 1.04388 -0.36569

H 4.35472 0.98368 -3.20506

H 4.09836 -1.56414 -3.16238

H 2.87287 -1.33511 -0.36201

O 2.03372 -2.32764 -1.95722

O 4.79142 -2.68974 -1.55798

H 4.27988 -3.43633 -1.88274

O 6.37375 -0.39801 -2.35023

H 6.99686 -0.92151 -1.83238

O 5.25990 2.05050 -1.67784

H 6.17885 1.81443 -1.86710

C 2.35484 2.31980 -1.84846

H 2.10298 2.27196 -2.91375

H 2.99414 3.18381 -1.67084

O 1.18848 2.49273 -1.05894

H 0.65526 1.68880 -1.08537

C 0.90342 -2.68542 -1.26053

C -1.38396 -3.55330 0.05034

C 0.71809 -4.04645 -1.03906

C -0.03775 -1.75582 -0.84331

C -1.18209 -2.19763 -0.17212

C -0.43337 -4.47281 -0.38811

H 1.46863 -4.74871 -1.38496

H 0.11322 -0.70764 -1.06381

H -2.26969 -3.91940 0.55434

O -0.68168 -5.78671 -0.13624

H 0.01015 -6.33252 -0.51953

C -2.15450 -1.15220 0.34159

C -3.56371 -1.63122 0.53101

H -3.77338 -2.17963 1.44714

C -4.58803 -1.39891 -0.32072

C -6.82887 -0.87096 -2.06629

C -5.93869 -1.81678 0.04223

C -4.41141 -0.72035 -1.60516

C -5.44835 -0.48732 -2.42044

C -7.00018 -1.55670 -0.74720

H -6.05988 -2.30725 1.00037

H -3.41532 -0.42202 -1.90740

H -5.33082 0.00169 -3.38025

O -7.76891 -0.64024 -2.79217

O -8.27864 -1.84963 -0.48234

C -8.55729 -2.43729 0.77347

H -8.22670 -1.78367 1.58681

H -8.06921 -3.41291 0.86340

H -9.63609 -2.56299 0.81421

H -2.45302 -0.01919 2.16568

H -2.17005 -0.33364 -0.37381

isorhapontin-isorhapontin 8-12 RS

106

Energy: -1871167.9493622

C 1.42759 3.21151 -0.21896

C 0.86374 5.16547 1.18003

C 0.67989 2.81542 1.97632

C 0.10366 4.22112 2.09784

O 0.62105 2.41444 0.61099

C 0.87987 4.63050 -0.24194

H 1.90237 5.24003 1.53613

H 1.72659 2.81213 2.31408

H -0.94617 4.19244 1.76884

H -0.14515 4.58790 -0.63372

H 2.46405 3.20295 0.15282

O 1.36312 2.71405 -1.52212

O 1.68933 5.50025 -1.00321

H 1.68647 5.19702 -1.91656

O 0.23328 6.42640 1.26313

H 0.66909 7.01921 0.64176

O 0.19963 4.62430 3.44379

H -0.02509 5.56038 3.48204

C -0.11698 1.78485 2.75822

H -1.18474 1.92113 2.56080

H 0.06391 1.91612 3.82444

O 0.29063 0.47445 2.40365

H 0.37219 0.45993 1.43946

C 1.76697 1.40143 -1.68857

C 2.48553 -1.26927 -2.14149

C 0.87555 0.53967 -2.30155

C 3.02440 0.94937 -1.30812

C 3.38190 -0.39194 -1.51518

C 1.24684 -0.77743 -2.51628

H 3.74089 1.64366 -0.88809

H 2.74247 -2.30747 -2.31004

O 0.26800 -1.51483 -3.09272

C 4.69005 -0.91267 -1.09269

H 5.00072 -1.83170 -1.58222

C 5.46337 -0.37637 -0.14007

H 5.09654 0.49197 0.40208

C 6.77072 -0.86838 0.30437

C 9.24607 -1.78370 1.25250

C 7.50659 -1.81386 -0.43255

C 7.30670 -0.38973 1.49850

C 8.53375 -0.84459 1.97406

C 8.72411 -2.26631 0.03767

H 7.12530 -2.17155 -1.37962

H 6.75222 0.34352 2.07302

H 8.95201 -0.47923 2.90354

O 9.53284 -3.17363 -0.58409

C 9.08876 -3.71544 -1.81255

H 8.96603 -2.92876 -2.56249

H 8.14380 -4.25054 -1.68159

H 9.86009 -4.41028 -2.13568

C -3.12429 -1.06029 1.54936

C -3.15603 -3.17856 2.84396

C -5.08562 -2.37784 1.53521

C -4.67156 -3.12030 2.80053

O -4.53440 -1.06004 1.55397

C -2.57049 -1.78100 2.77271

H -2.80505 -3.74769 1.96950

H -4.70966 -2.92574 0.66085

H -5.03108 -2.56615 3.67991

H -2.87878 -1.21282 3.66128

H -2.75908 -1.52942 0.62458

O -2.70885 0.27175 1.64644

O -1.17484 -1.92124 2.70464

H -0.74227 -1.04993 2.74120

O -2.77974 -3.84160 4.03289

H -1.81940 -3.79419 4.09697

O -5.23368 -4.41219 2.76024

H -4.80146 -4.93318 3.44587

C -6.58500 -2.20698 1.40914

H -6.96351 -1.66751 2.28519

H -7.05875 -3.18664 1.36068

O -6.91905 -1.51296 0.21660

H -6.38597 -0.70959 0.18528

C -2.46620 0.97625 0.49499

C -1.94818 2.54703 -1.72237

C -3.00363 2.26106 0.43074

C -1.64980 0.48307 -0.50924

C -1.41685 1.26821 -1.63825

C -2.71889 3.05053 -0.67512

H -3.62759 2.61258 1.24555

H -1.21033 -0.50567 -0.42910

H -1.75473 3.17999 -2.58096

O -3.17441 4.32820 -0.80048

H -3.65608 4.58542 -0.00921

C -0.54153 0.75553 -2.76128

C -0.86974 -0.66827 -3.36827

C -2.13175 -1.35053 -2.89165

C -4.50231 -2.55326 -1.99663

C -3.33656 -0.63674 -2.90773

C -2.12390 -2.65817 -2.43242

C -3.30942 -3.25622 -1.99779

C -4.50223 -1.21207 -2.43197

H -3.34433 0.39082 -3.24905

H -1.19677 -3.21441 -2.40887

H -3.32416 -4.28249 -1.64989

O -5.70000 -0.57175 -2.33060

C -5.71816 0.81522 -2.60836

H -4.99901 1.34846 -1.97591

H -6.72886 1.15334 -2.39013

H -5.49156 1.00897 -3.66107

H -0.57145 1.49193 -3.56781

H -0.90964 -0.56053 -4.45613

O -5.64038 -3.16193 -1.59960

H -6.27874 -2.50027 -1.27422

O 10.44261 -2.22900 1.70048

H 10.78236 -2.86924 1.06354

isorhapontin-isorhapontin 8-12 SS

106

Energy: -1871167.9493622

C 1.42759 3.21151 -0.21896

C 0.86374 5.16547 1.18003

C 0.67989 2.81542 1.97632

C 0.10366 4.22112 2.09784

O 0.62105 2.41444 0.61099

C 0.87987 4.63050 -0.24194

H 1.90237 5.24003 1.53613

H 1.72659 2.81213 2.31408

H -0.94617 4.19244 1.76884

H -0.14515 4.58790 -0.63372

H 2.46405 3.20295 0.15282

O 1.36312 2.71405 -1.52212

O 1.68933 5.50025 -1.00321

H 1.68647 5.19702 -1.91656

O 0.23328 6.42640 1.26313

H 0.66909 7.01921 0.64176

O 0.19963 4.62430 3.44379

H -0.02509 5.56038 3.48204

C -0.11698 1.78485 2.75822

H -1.18474 1.92113 2.56080

H 0.06391 1.91612 3.82444

O 0.29063 0.47445 2.40365

H 0.37219 0.45993 1.43946

C 1.76697 1.40143 -1.68857

C 2.48553 -1.26927 -2.14149

C 0.87555 0.53967 -2.30155

C 3.02440 0.94937 -1.30812

C 3.38190 -0.39194 -1.51518

C 1.24684 -0.77743 -2.51628

H 3.74089 1.64366 -0.88809

H 2.74247 -2.30747 -2.31004

O 0.26800 -1.51483 -3.09272

C 4.69005 -0.91267 -1.09269

H 5.00072 -1.83170 -1.58222

C 5.46337 -0.37637 -0.14007

H 5.09654 0.49197 0.40208

C 6.77072 -0.86838 0.30437

C 9.24607 -1.78370 1.25250

C 7.50659 -1.81386 -0.43255

C 7.30670 -0.38973 1.49850

C 8.53375 -0.84459 1.97406

C 8.72411 -2.26631 0.03767

H 7.12530 -2.17155 -1.37962

H 6.75222 0.34352 2.07302

H 8.95201 -0.47923 2.90354

O 9.53284 -3.17363 -0.58409

C 9.08876 -3.71544 -1.81255

H 8.96603 -2.92876 -2.56249

H 8.14380 -4.25054 -1.68159

H 9.86009 -4.41028 -2.13568

C -3.12429 -1.06029 1.54936

C -3.15603 -3.17856 2.84396

C -5.08562 -2.37784 1.53521

C -4.67156 -3.12030 2.80053

O -4.53440 -1.06004 1.55397

C -2.57049 -1.78100 2.77271

H -2.80505 -3.74769 1.96950

H -4.70966 -2.92574 0.66085

H -5.03108 -2.56615 3.67991

H -2.87878 -1.21282 3.66128

H -2.75908 -1.52942 0.62458

O -2.70885 0.27175 1.64644

O -1.17484 -1.92124 2.70464

H -0.74227 -1.04993 2.74120

O -2.77974 -3.84160 4.03289

H -1.81940 -3.79419 4.09697

O -5.23368 -4.41219 2.76024

H -4.80146 -4.93318 3.44587

C -6.58500 -2.20698 1.40914

H -6.96351 -1.66751 2.28519

H -7.05875 -3.18664 1.36068

O -6.91905 -1.51296 0.21660

H -6.38597 -0.70959 0.18528

C -2.46620 0.97625 0.49499

C -1.94818 2.54703 -1.72237

C -3.00363 2.26106 0.43074

C -1.64980 0.48307 -0.50924

C -1.41685 1.26821 -1.63825

C -2.71889 3.05053 -0.67512

H -3.62759 2.61258 1.24555

H -1.21033 -0.50567 -0.42910

H -1.75473 3.17999 -2.58096

O -3.17441 4.32820 -0.80048

H -3.65608 4.58542 -0.00921

C -0.54153 0.75553 -2.76128

C -0.86974 -0.66827 -3.36827

C -2.13175 -1.35053 -2.89165

C -4.50231 -2.55326 -1.99663

C -3.33656 -0.63674 -2.90773

C -2.12390 -2.65817 -2.43242

C -3.30942 -3.25622 -1.99779

C -4.50223 -1.21207 -2.43197

H -3.34433 0.39082 -3.24905

H -1.19677 -3.21441 -2.40887

H -3.32416 -4.28249 -1.64989

O -5.70000 -0.57175 -2.33060

C -5.71816 0.81522 -2.60836

H -4.99901 1.34846 -1.97591

H -6.72886 1.15334 -2.39013

H -5.49156 1.00897 -3.66107

H -0.57145 1.49193 -3.56781

H -0.90964 -0.56053 -4.45613

O -5.64038 -3.16193 -1.59960

H -6.27874 -2.50027 -1.27422

O 10.44261 -2.22900 1.70048

H 10.78236 -2.86924 1.06354

coniferyl alcohol-astringin beta-O-4 quinone methide

74

Energy: -1295725.3438392

C -4.67025 0.08619 0.30520

C -5.69921 -2.03029 1.05703

C -3.58972 -1.91484 -0.23329

C -4.83079 -2.75868 0.03824

O -4.02377 -0.64215 -0.69884

C -5.98928 -0.60060 0.62755

H -5.14772 -1.99638 2.00872

H -3.02851 -1.79423 0.70673

H -5.39857 -2.86990 -0.89632

H -6.59455 -0.60574 -0.28778

H -4.02856 0.15052 1.20006

O -4.95508 1.37328 -0.15264

O -6.67938 0.01877 1.69316

H -6.87772 0.92417 1.43539

O -6.88706 -2.77995 1.21796

H -7.44895 -2.31275 1.84484

O -4.43051 -4.01540 0.53616

H -5.22429 -4.47131 0.83565

C -2.63884 -2.46095 -1.27800

H -3.14373 -2.45551 -2.24938

H -2.37257 -3.48967 -1.02192

O -1.50095 -1.62662 -1.34670

H -0.75448 -2.10794 -0.96735

C -3.89705 2.24435 -0.28060

C -1.88942 4.15538 -0.52958

C -4.22322 3.59660 -0.22115

C -2.58380 1.83197 -0.47367

C -1.57582 2.79895 -0.58273

C -3.21308 4.54338 -0.35262

H -5.25834 3.88734 -0.07948

H -2.33400 0.78425 -0.58649

H -1.13464 4.92077 -0.65805

O -3.46998 5.88101 -0.31552

H -4.41576 6.02911 -0.23604

C -0.19796 2.31370 -0.75505

H -0.11308 1.29797 -1.13855

C 0.90999 2.97728 -0.40522

H 0.82738 3.95093 0.07154

C 2.26475 2.41226 -0.51253

C 4.76204 1.17552 -0.49466

C 3.21091 2.74508 0.46182

C 2.60712 1.49501 -1.51441

C 3.85315 0.88188 -1.50018

C 4.45832 2.13133 0.48353

H 2.97857 3.46541 1.23832

H 1.89850 1.26334 -2.29993

H 4.13346 0.15525 -2.25413

O 5.33146 2.44560 1.46414

H 6.17051 1.96787 1.35449

C 4.34514 -2.29403 -0.14541

C 1.69952 -3.28618 -0.67244

C 3.18300 -1.63485 0.43818

C 4.12038 -3.51779 -0.91726

C 2.89160 -3.99085 -1.16162

C 1.93813 -2.08316 0.17952

H 3.32794 -0.73937 1.02760

H 4.99590 -4.02520 -1.30899

H 2.71346 -4.88305 -1.74916

O 0.56932 -3.63965 -0.94683

C 5.60471 -1.81199 -0.07094

H 6.39168 -2.35892 -0.58617

C 6.01313 -0.51799 0.56515

H 5.34980 -0.25631 1.39620

C 7.43121 -0.54628 1.09304

H 8.12096 -0.63778 0.24693

H 7.55885 -1.40293 1.76351

O 7.62535 0.67598 1.78668

H 8.56134 0.84157 1.91674

O 5.98310 0.54101 -0.41334

O 0.78761 -1.54026 0.59073

C 0.85343 -0.36568 1.38564

H 1.31885 -0.58761 2.35082

H -0.17562 -0.04420 1.52924

H 1.41459 0.41875 0.87225

coniferyl alcohol-astringin beta-O-4 RS

74

Energy: -1295745.2041688

C 5.97924 0.48229 -0.42335

C 7.52570 -1.27923 -1.18950

C 5.46826 -1.74178 0.12193

C 6.88623 -2.21636 -0.17423

O 5.52835 -0.39451 0.57974

C 7.43521 0.16896 -0.73319

H 6.97889 -1.37449 -2.13957

H 4.86576 -1.79169 -0.79797

H 7.47403 -2.17663 0.75414

H 8.01598 0.30588 0.18779

H 5.36211 0.36941 -1.32937

O 5.90360 1.79010 0.04233

O 7.94121 0.96306 -1.78323

H 7.89950 1.88614 -1.51479

O 8.86412 -1.69546 -1.35914

H 9.28579 -1.09723 -1.98487

O 6.81880 -3.53276 -0.67007

H 7.69858 -3.77016 -0.98278

C 4.76261 -2.52619 1.21030

H 5.38158 -2.52712 2.11575

H 4.61774 -3.55476 0.88257

O 3.48737 -1.97112 1.46727

H 3.62921 -1.06886 1.77605

C 4.64946 2.33540 0.20219

C 2.21309 3.61167 0.55847

C 4.62993 3.69451 0.51376

C 3.47633 1.61115 0.06578

C 2.24322 2.25170 0.26406

C 3.40482 4.32376 0.68346

H 5.56684 4.23145 0.60929

H 3.49939 0.55929 -0.17572

H 1.27564 4.13080 0.71598

O 3.30551 5.64773 0.98067

H 4.18211 6.03386 1.05507

C 0.97916 1.50264 0.20315

H 0.08032 2.11061 0.14686

C 0.87892 0.16892 0.27091

H 1.77817 -0.42959 0.39234

C -0.35509 -0.62213 0.24531

C -2.65612 -2.22225 0.27701

C -1.59391 -0.09050 -0.11971

C -0.28427 -1.97618 0.59913

C -1.42354 -2.76510 0.61880

C -2.73341 -0.87836 -0.10900

H -1.70004 0.93844 -0.44228

H 0.67587 -2.40081 0.87261

H -1.38366 -3.81049 0.90031

O -3.92196 -0.30563 -0.46968

C -6.24996 -0.50860 -0.95749

C -8.72963 0.75372 -1.14511

C -6.44085 0.73695 -0.35228

C -7.29323 -1.10564 -1.65176

C -8.53330 -0.47725 -1.74455

C -7.67318 1.36298 -0.44889

H -5.61690 1.19780 0.17525

H -7.14466 -2.06664 -2.13294

H -9.35681 -0.92706 -2.28476

C -4.94114 -1.23997 -0.80778

C -5.00873 -2.33121 0.26467

H -5.74512 -3.08313 -0.02443

C -5.34042 -1.81027 1.65837

H -6.33964 -1.37351 1.65588

H -4.62275 -1.03068 1.94632

O -5.34933 -2.86520 2.59361

H -4.51609 -3.33903 2.50578

O -3.75998 -3.02959 0.30757

O -7.99065 2.57702 0.08669

C -6.98432 3.25336 0.81662

H -6.66512 2.66011 1.67818

H -7.43026 4.18424 1.15820

H -6.12221 3.46985 0.17969

O -9.93059 1.37298 -1.23604

H -9.87144 2.21218 -0.76371

H -4.67115 -1.72322 -1.75525

coniferyl alcohol-astringin beta-O-4 RR

74

Energy: -1295747.6448481

C -6.12327 0.24781 0.59289

C -7.43346 -1.69321 1.36431

C -5.48065 -1.88087 -0.15867

C -6.81826 -2.50033 0.22950

O -5.69704 -0.51765 -0.50810

C -7.51110 -0.21668 1.00790

H -6.79077 -1.79891 2.25119

H -4.78924 -1.93699 0.69612

H -7.49440 -2.45409 -0.63632

H -8.18655 -0.07413 0.15485

H -5.41552 0.13197 1.42971

O -6.20452 1.58334 0.21920

O -7.98004 0.45878 2.15358

H -8.04329 1.39721 1.94956

O -8.70865 -2.24065 1.62488

H -9.11897 -1.72050 2.32374

O -6.59273 -3.83497 0.61939

H -7.41492 -4.16984 0.99356

C -4.82220 -2.52430 -1.36312

H -5.52645 -2.52356 -2.20401

H -4.55701 -3.55410 -1.12747

O -3.63203 -1.83717 -1.69737

H -3.88191 -0.93151 -1.91423

C -5.02422 2.25460 -0.00809

C -2.75056 3.76408 -0.47904

C -5.15122 3.62305 -0.22056

C -3.78330 1.63029 -0.02343

C -2.63429 2.38827 -0.27905

C -4.00528 4.37075 -0.44973

H -6.12304 4.09761 -0.20046

H -3.69341 0.56776 0.14514

H -1.86405 4.35827 -0.67884

O -4.16923 5.70735 -0.64478

H -3.31414 6.12142 -0.78803

C -1.30987 1.75666 -0.37734

H -0.46255 2.43562 -0.33086

C -1.10364 0.44769 -0.57104

H -1.95704 -0.21635 -0.68293

C 0.19089 -0.22845 -0.69892

C 2.60655 -1.60434 -1.02640

C 1.40346 0.38015 -0.36755

C 0.20714 -1.54826 -1.16983

C 1.40428 -2.22642 -1.33702

C 2.60229 -0.29739 -0.52382

H 1.44595 1.38231 0.04201

H -0.73154 -2.03192 -1.41853

H 1.43321 -3.24034 -1.71715

O 3.75972 0.34020 -0.17871

C 6.11712 0.29854 0.16673

C 8.49801 1.73190 0.37771

C 7.16561 -0.20560 0.94585

C 6.26529 1.50878 -0.49441

C 7.45567 2.22773 -0.38338

C 8.35017 0.50353 1.04443

H 7.04186 -1.16050 1.44352

H 5.44498 1.90171 -1.08178

H 7.58943 3.17883 -0.88361

C 4.87138 -0.52970 0.01497

C 4.95136 -1.48874 -1.17853

H 5.01019 -0.89269 -2.09925

C 6.11035 -2.46467 -1.11387

H 7.05460 -1.92296 -1.16463

H 6.04490 -3.13684 -1.97600

O 6.11218 -3.19231 0.09979

H 5.28382 -3.68123 0.14409

O 3.77286 -2.29408 -1.20862

O 9.44795 0.12147 1.75922

C 9.38340 -1.12108 2.43493

H 8.59371 -1.10995 3.19137

H 10.34904 -1.25607 2.91601

H 9.20288 -1.93813 1.73045

O 9.65707 2.42551 0.48522

H 10.25459 1.92297 1.05199

H 4.70163 -1.12635 0.91864

coniferyl alcohol-isorhapontin beta-O-4 quinone methide

77

Energy: -1320375.1671170

C 5.32454 -0.28772 -0.79904

C 5.48657 -1.85850 1.10424

C 4.06386 -2.33573 -0.89107

C 4.21007 -2.51716 0.61986

O 4.20124 -0.97193 -1.27164

C 5.46368 -0.39590 0.72023

H 6.34861 -2.34471 0.62247

H 4.83224 -2.94799 -1.38566

H 3.35374 -2.04394 1.11684

H 4.60569 0.08316 1.20647

H 6.24344 -0.66177 -1.26906

O 5.24082 1.05142 -1.21609

O 6.67781 0.18002 1.16044

H 6.64850 1.12805 0.99725

O 5.55622 -2.03361 2.50525

H 6.30866 -1.52617 2.82717

O 4.23329 -3.90573 0.87186

H 4.42324 -4.02164 1.80862

C 2.68974 -2.75850 -1.37271

H 2.52429 -3.80072 -1.08181

H 2.66380 -2.68044 -2.46585

O 1.73828 -1.89839 -0.78281

H 0.86342 -2.29316 -0.90302

C 4.26452 1.86694 -0.69011

C 2.44020 3.69710 0.34141

C 4.64830 3.18875 -0.49932

C 2.96878 1.44352 -0.38514

C 2.05849 2.36780 0.13176

C 3.72874 4.09867 0.01401

H 5.65274 3.50844 -0.74609

H 2.65972 0.42047 -0.56221

H 1.74769 4.40565 0.78420

O 4.16545 5.37597 0.19658

H 3.45556 5.91134 0.56047

C 0.69720 1.89438 0.42781

H 0.59834 0.81843 0.56269

C -0.39856 2.66155 0.47070

H -0.32565 3.71862 0.22560

C -1.75069 2.14483 0.73614

C -4.32016 1.08806 1.03966

C -2.83347 2.70331 0.03729

C -1.97953 1.08596 1.61338

C -3.26296 0.56619 1.76282

C -4.11079 2.17460 0.17060

H -2.64785 3.53263 -0.63390

H -1.15715 0.67187 2.18355

H -3.46051 -0.26403 2.43077

O -5.20708 2.61070 -0.50043

C -4.28177 -2.17129 -0.10023

C -1.67788 -3.31304 -0.52987

C -3.26758 -1.43082 -0.84156

C -3.95441 -3.53146 0.32724

C -2.74511 -4.07401 0.12725

C -2.04121 -1.95612 -1.03471

H -3.49604 -0.43095 -1.18743

H -4.73186 -4.08826 0.84001

H -2.48887 -5.07222 0.46019

O -0.54474 -3.73796 -0.65791

C -5.47800 -1.66401 0.26926

H -6.14529 -2.28835 0.85811

C -5.97261 -0.26769 0.03165

H -5.57530 0.16265 -0.89138

C -7.49093 -0.19060 0.00303

H -7.88154 -0.84389 -0.77960

H -7.77609 0.84349 -0.21869

O -8.04674 -0.62715 1.22286

H -7.68611 -0.06493 1.91681

O -5.58087 0.56422 1.15045

O -1.00105 -1.37443 -1.64428

C -1.16070 -0.05223 -2.12734

H -1.89437 -0.02968 -2.93968

H -0.18256 0.24961 -2.49460

H -1.47695 0.61635 -1.32269

C -5.04880 3.72763 -1.35440

H -4.69789 4.60028 -0.79628

H -6.03371 3.93249 -1.76712

H -4.35019 3.50731 -2.16742

coniferyl alcohol-isorhapontin beta-O-4 RR

80

Energy: -1368366.5088393

C 4.25124 -0.53591 -0.18028

C 3.95870 -2.88975 -0.72334

C 2.40995 -1.61418 0.77825

C 2.97141 -2.99096 0.43084

O 3.49718 -0.72179 0.99726

C 5.01242 -1.81871 -0.46644

H 3.40190 -2.60057 -1.62760

H 1.79986 -1.23972 -0.05608

H 3.49943 -3.39409 1.30539

H 5.62048 -2.07921 0.40872

H 3.58083 -0.32627 -1.02733

O 5.12790 0.52715 -0.00481

O 5.79437 -1.73400 -1.63571

H 6.43466 -1.02392 -1.52498

O 4.52206 -4.17341 -0.89367

H 5.15512 -4.12765 -1.61805

O 1.88767 -3.84008 0.08285

H 2.26521 -4.64770 -0.28601

C 1.57386 -1.61701 2.04334

H 2.15934 -2.05151 2.86334

H 0.67934 -2.22044 1.88098

O 1.15452 -0.30494 2.36699

H 1.95020 0.23158 2.46338

C 4.55592 1.78301 -0.04177

C 3.50564 4.35346 -0.17896

C 5.42312 2.86800 -0.03850

C 3.18210 1.96824 -0.10545

C 2.64452 3.25694 -0.15878

C 4.88440 4.14826 -0.11154

H 6.49486 2.72690 0.00285

H 2.50524 1.13025 -0.12301

H 3.10601 5.36214 -0.21977

O 5.76919 5.18266 -0.11443

H 5.29433 6.01562 -0.17588

C 1.17670 3.40398 -0.18327

H 0.76556 4.27959 -0.67826

C 0.38627 2.48079 0.37962

H 0.86599 1.67608 0.92856

C -1.07143 2.34957 0.35942

C -3.80266 1.80212 0.32195

C -1.61221 1.37583 1.21301

C -1.91516 3.06442 -0.48996

C -3.27709 2.78591 -0.50170

C -2.97263 1.11013 1.21397

H -0.93521 0.81536 1.84667

H -1.51151 3.81023 -1.16408

H -3.95084 3.30974 -1.17163

O -3.57293 0.18083 2.00374

C -3.77282 -1.09689 -0.94119

C -1.07927 -1.81662 -1.33459

C -3.14279 -1.99155 -0.08039

C -3.03093 -0.57097 -2.00171

C -1.70141 -0.92136 -2.19915

C -1.81084 -2.33249 -0.26355

H -3.68645 -2.41730 0.75488

H -3.48482 0.12690 -2.69610

H -1.13009 -0.51284 -3.02364

C -5.24567 -0.74911 -0.72238

C -5.57092 0.74672 -0.87437

H -5.09084 1.17012 -1.76207

C -7.06679 0.99795 -0.95659

H -7.55361 0.55055 -0.08378

H -7.46512 0.52771 -1.85756

O -7.34085 2.37859 -1.05535

H -6.94173 2.79476 -0.28437

O -5.13642 1.47794 0.28297

O -1.14809 -3.16101 0.61926

C -1.39775 -4.54788 0.41220

H -2.45497 -4.77669 0.57094

H -0.78693 -5.08641 1.13450

H -1.11368 -4.84192 -0.60356

C -2.73136 -0.58088 2.85835

H -3.38725 -1.27530 3.37978

H -2.22935 0.06613 3.58240

H -1.98645 -1.13782 2.28213

H -5.83354 -1.28039 -1.48040

O 0.21019 -2.17745 -1.54628

H 0.49483 -2.82378 -0.87676

O -5.72085 -1.20852 0.52168

H -5.28964 -0.66422 1.19821

coniferyl alcohol-isorhapontin beta-O-4 RS

80

Energy: -1368364.9141620

C 4.77848 -0.01032 -0.10867

C 5.63231 -2.28064 -0.60095

C 3.44677 -1.84121 0.50370

C 4.63404 -2.79888 0.41729

O 3.93373 -0.56124 0.87673

C 6.03574 -0.85948 -0.24779

H 5.15667 -2.27413 -1.59286

H 2.94773 -1.79230 -0.47723

H 5.12229 -2.83432 1.40114

H 6.55633 -0.85321 0.71838

H 4.24456 0.04458 -1.07137

O 5.18073 1.26015 0.28559

O 6.87159 -0.39707 -1.28410

H 7.15186 0.49907 -1.07221

O 6.73352 -3.16384 -0.59691

H 7.39410 -2.82238 -1.20885

O 4.15438 -4.08173 0.06206

H 4.91469 -4.64119 -0.13293

C 2.41693 -2.26317 1.54249

H 1.70898 -1.44714 1.68593

H 2.91499 -2.46936 2.49693

O 1.66907 -3.38058 1.10462

H 2.28541 -4.06318 0.80594

C 4.27221 2.28791 0.17908

C 2.57265 4.46933 0.02167

C 4.80046 3.56799 0.29634

C 2.91247 2.07818 -0.01101

C 2.05516 3.17988 -0.10616

C 3.93975 4.65442 0.21901

H 5.85997 3.71738 0.45563

H 2.50393 1.07893 -0.00697

H 1.91066 5.32767 -0.04126

O 4.49543 5.89006 0.34127

H 3.81404 6.56268 0.26138

C 0.61070 2.98765 -0.30348

H -0.02534 3.80079 0.03642

C 0.06920 1.90475 -0.87583

H 0.73443 1.14567 -1.28048

C -1.35638 1.59839 -1.02514

C -4.03260 0.78710 -1.18080

C -2.35402 2.31946 -0.34692

C -1.73202 0.52285 -1.82797

C -3.06375 0.12953 -1.91304

C -3.68173 1.93344 -0.43930

H -2.08118 3.16635 0.26868

H -0.97328 -0.04268 -2.35738

H -3.35515 -0.73967 -2.48840

O -4.71837 2.57391 0.16079

C -4.23840 -2.11714 -0.01460

C -1.58922 -2.89261 -0.50036

C -3.19909 -1.61773 0.78456

C -3.94091 -3.00980 -1.03216

C -2.62260 -3.39974 -1.27050

C -1.88585 -1.97603 0.53077

H -3.43396 -0.91483 1.57487

H -4.73755 -3.39767 -1.65796

H -2.37297 -4.10080 -2.05760

C -5.65665 -1.65233 0.21016

C -5.83777 -0.13436 0.08233

H -5.30975 0.35342 0.90673

C -7.30378 0.25673 0.12194

H -7.77312 -0.20102 0.99264

H -7.78216 -0.13606 -0.78606

O -7.47949 1.65203 0.22157

H -6.80120 2.06566 -0.32438

O -5.32826 0.34488 -1.17800

O -0.79733 -1.48984 1.19229

C -1.02307 -0.43318 2.10622

H -1.51848 0.40637 1.60759

H -0.04331 -0.11768 2.46052

H -1.62408 -0.77042 2.95599

C -4.43158 3.65068 1.03098

H -3.96375 4.47863 0.49018

H -3.77836 3.32861 1.84784

H -5.39014 3.96914 1.43441

H -6.30157 -2.13841 -0.53464

O -0.32059 -3.25328 -0.77331

H 0.27242 -3.11080 -0.01026

O -6.11236 -1.95270 1.52675

H -5.84886 -2.85359 1.73337

coniferyl alcohol-isorhapontin 5-beta RS quinone methide

77

Energy: -1320370.8675790

C 3.86352 0.02814 -0.06980

C 4.55356 -2.31125 -0.37523

C 2.58033 -1.64430 0.95253

C 3.68865 -2.68087 0.82083

O 3.18207 -0.35775 1.10415

C 5.06712 -0.88319 -0.25044

H 3.93309 -2.37593 -1.28109

H 1.96852 -1.66029 0.04396

H 4.31321 -2.66552 1.72582

H 5.70815 -0.79240 0.63616

H 3.19815 -0.04282 -0.93944

O 4.31850 1.34047 0.08677

O 5.78569 -0.59987 -1.43130

H 6.08102 0.31497 -1.39464

O 5.61092 -3.24820 -0.43577

H 6.17393 -3.00982 -1.17962

O 3.09830 -3.95333 0.64930

H 3.79713 -4.55248 0.36371

C 1.68980 -1.84319 2.15914

H 2.29814 -1.85396 3.07187

H 1.16947 -2.79696 2.07390

O 0.70776 -0.82047 2.22373

H 1.18886 0.01621 2.24692

C 3.36605 2.32768 0.11793

C 1.57814 4.45354 0.24174

C 3.85283 3.62931 0.24043

C 2.00386 2.08050 0.03964

C 1.10269 3.14991 0.13537

C 2.95225 4.68427 0.28842

H 4.92365 3.79136 0.29336

H 1.63345 1.07459 -0.08993

H 0.90194 5.29629 0.31759

O 3.35849 5.97886 0.39770

H 4.31757 6.01832 0.43815

C -0.34777 2.90141 0.19236

H -0.99432 3.76380 0.04697

C -0.88560 1.71254 0.48648

H -0.23457 0.87147 0.71393

C -2.31252 1.38280 0.57140

C -5.03274 0.60202 1.03145

C -2.64277 0.29619 1.49810

C -3.26838 1.94655 -0.18867

C -4.66404 1.42125 -0.19059

C -3.91572 -0.08274 1.73371

H -1.81022 -0.17880 2.00708

H -3.01213 2.70505 -0.92156

O -6.18673 0.46442 1.37466

O -4.32736 -1.03039 2.58975

C -3.31873 -1.70494 3.31899

H -2.75729 -1.00433 3.94402

H -2.62530 -2.21553 2.64290

H -3.83223 -2.43152 3.94310

H -5.40126 2.22502 -0.27130

C -4.87563 0.51913 -1.49498

H -4.58995 1.17203 -2.32445

C -6.35311 0.13536 -1.72292

H -6.46381 -0.14393 -2.77301

H -6.98526 1.01186 -1.53934

O -6.78935 -0.97511 -0.97612

H -6.99210 -0.68516 -0.08004

C -3.99208 -0.68361 -1.42541

H -4.42704 -1.53978 -0.91516

C -2.68913 -0.74737 -1.78339

C 0.17104 -0.71656 -2.07032

C -2.00482 0.32474 -2.49466

C -1.88494 -1.87922 -1.33971

C -0.54005 -1.86519 -1.44205

C -0.67052 0.33628 -2.64397

H -2.59586 1.13037 -2.91254

H -2.40449 -2.69838 -0.85676

H -0.14859 1.13521 -3.15672

O 1.38978 -0.66524 -2.08981

O 0.31071 -2.80451 -0.99632

C -0.22943 -3.89093 -0.26994

H -0.80283 -3.52740 0.59016

H 0.62646 -4.47020 0.06994

H -0.87686 -4.50207 -0.90659

coniferyl alcohol-isorhapontin 5-beta RR quinone methide

77

Energy: -1320368.0606413

C 3.84886 1.07684 -0.08474

C 5.41692 -0.76477 -0.51401

C 3.57291 -0.90860 1.14503

C 4.99864 -1.35279 0.82105

O 3.54675 0.51433 1.17135

C 5.28480 0.74909 -0.46502

H 4.74840 -1.15190 -1.29629

H 2.90350 -1.29463 0.36656

H 5.67192 -0.97094 1.60202

H 5.95198 1.15675 0.30587

H 3.16711 0.69624 -0.85607

O 3.73655 2.46713 0.01850

O 5.62277 1.24016 -1.74338

H 5.50465 2.19488 -1.74188

O 6.75066 -1.17319 -0.75173

H 7.03399 -0.78476 -1.58579

O 5.04764 -2.76999 0.79288

H 5.89318 -3.01936 0.40262

C 3.09359 -1.39342 2.50552

H 2.15690 -0.88804 2.74604

H 3.83217 -1.12212 3.27100

O 2.83568 -2.78222 2.50252

H 3.61779 -3.21767 2.14124

C 2.47453 2.98902 0.11973

C -0.01468 4.23034 0.32254

C 2.39563 4.37809 0.13943

C 1.32205 2.21548 0.19854

C 0.07263 2.83948 0.29935

C 1.14849 4.98679 0.24157

H 3.30848 4.96038 0.08008

H 1.37454 1.13529 0.21766

H -0.96098 4.74754 0.41255

O 1.01050 6.34112 0.26754

H 1.87497 6.75895 0.23466

C -1.09659 1.95540 0.37983

H -0.85154 0.89758 0.35984

C -2.38828 2.29932 0.44411

H -2.67916 3.34618 0.41581

C -3.48430 1.31617 0.50439

C -5.79214 -0.39878 0.68640

C -4.80172 1.75317 0.02510

C -3.31833 0.05861 0.94914

C -4.39656 -0.96754 0.83250

C -5.89726 0.96845 0.10397

H -4.87542 2.75826 -0.37367

H -2.36396 -0.26703 1.34512

O -6.77406 -1.05092 0.96442

O -7.14891 1.28787 -0.25702

C -7.36040 2.59159 -0.76155

H -6.77847 2.75580 -1.67403

H -7.09008 3.34449 -0.01470

H -8.42158 2.66010 -0.98582

H -4.40378 -1.63972 1.69450

C -4.12439 -1.86563 -0.44315

H -4.11731 -1.20267 -1.31273

C -5.23545 -2.91344 -0.65568

H -4.89723 -3.62426 -1.41320

H -6.13875 -2.42768 -1.03480

O -5.50949 -3.64782 0.51477

H -6.13113 -3.12412 1.03559

C -2.80618 -2.55770 -0.28591

H -2.84254 -3.45772 0.32683

C -1.61353 -2.17531 -0.79646

C 0.97571 -1.29192 -1.69586

C -1.45596 -0.96155 -1.59163

C -0.41578 -2.96259 -0.52188

C 0.80377 -2.56960 -0.94725

C -0.25009 -0.54856 -2.01243

H -2.33056 -0.36340 -1.81639

H -0.54146 -3.87522 0.04770

H -0.11701 0.36643 -2.57820

O 2.08080 -0.88104 -2.00234

O 1.96690 -3.20580 -0.75687

C 1.93268 -4.42314 -0.03115

H 1.53905 -4.25779 0.97454

H 2.96642 -4.75448 0.03337

H 1.33216 -5.16728 -0.56409

coniferyl alcohol-isorhapontin 5-beta RS

77

Energy: -1320402.8336707

C -5.25665 -0.81706 -0.02608

C -5.55303 -3.15046 0.73535

C -3.39657 -2.24127 -0.11196

C -4.27840 -3.48521 -0.01702

O -4.13462 -1.22834 -0.77956

C -6.24777 -1.96866 0.07955

H -5.29292 -2.87290 1.76762

H -3.12268 -1.91070 0.90207

H -4.54416 -3.79883 -1.03594

H -6.56104 -2.24045 -0.93657

H -4.93273 -0.49760 0.97759

O -5.90148 0.23179 -0.67148

O -7.35514 -1.64137 0.88771

H -7.80830 -0.89030 0.49112

O -6.35760 -4.31059 0.73415

H -7.19164 -4.09697 1.16570

O -3.54968 -4.50886 0.63573

H -4.15566 -5.23715 0.81340

C -2.11244 -2.48452 -0.89513

H -1.63837 -1.52439 -1.10397

H -2.34553 -2.96779 -1.85097

O -1.18816 -3.24981 -0.14222

H -1.63902 -4.04229 0.17711

C -5.30604 1.47444 -0.62581

C -4.22267 4.02980 -0.56745

C -6.10535 2.55502 -0.97613

C -3.98385 1.65188 -0.24750

C -3.42378 2.93320 -0.24407

C -5.55299 3.83024 -0.93525

H -7.13886 2.41530 -1.26354

H -3.37210 0.80835 0.02883

H -3.80106 5.03035 -0.56864

O -6.37279 4.86309 -1.27477

H -5.89078 5.69199 -1.21504

C -1.99199 3.07480 0.07983

H -1.68038 3.97360 0.60489

C -1.12068 2.11162 -0.24896

H -1.51140 1.28157 -0.83321

C 0.28956 1.94596 0.09991

C 2.87650 1.37479 0.90786

C 1.02416 2.88169 0.85076

C 0.88321 0.73753 -0.27221

C 2.17108 0.45948 0.15243

C 2.32889 2.61607 1.25644

H 0.55454 3.81491 1.13298

H 0.34112 0.00860 -0.86086

O 3.11505 3.44645 1.98315

C 2.55983 4.68282 2.37709

H 1.67982 4.53584 3.01145

H 2.28510 5.28766 1.50694

H 3.33457 5.19355 2.94410

C 3.03742 -0.75843 -0.02665

H 3.44499 -0.79710 -1.04318

C 2.31346 -2.07098 0.24055

H 1.80817 -2.00679 1.21278

H 3.04559 -2.88938 0.28444

O 1.38658 -2.28117 -0.80324

H 0.63458 -2.80039 -0.48554

C 4.16775 -0.47295 0.99786

C 5.54358 -0.86069 0.53965

C 8.03999 -1.64151 -0.42805

C 6.10956 -2.06084 0.94078

C 6.23374 -0.03127 -0.35208

C 7.47387 -0.41760 -0.82726

C 7.35583 -2.45644 0.45277

H 5.58387 -2.69752 1.64404

H 5.79207 0.91480 -0.63854

H 7.81335 -3.38957 0.75675

O 8.25684 0.29933 -1.68585

C 7.76138 1.54783 -2.13045

H 6.82451 1.41995 -2.68008

H 8.52050 1.95840 -2.79191

H 7.60205 2.22469 -1.28648

O 9.25632 -2.01064 -0.89953

H 9.57190 -1.31341 -1.48684

H 3.93445 -0.98655 1.93956

O 4.11341 0.94823 1.27182

coniferyl alcohol-isorhapontin 5-beta RR

77

Energy: -1320402.7352646

C -4.45890 -0.96876 0.32841

C -4.35848 -3.41474 0.66437

C -2.68536 -2.13541 -0.66394

C -3.41555 -3.46994 -0.52360

O -3.65921 -1.11466 -0.82612

C -5.29519 -2.22539 0.52988

H -3.76522 -3.28855 1.58217

H -2.08500 -1.95169 0.24078

H -4.00798 -3.63677 -1.43383

H -5.93375 -2.35462 -0.35326

H -3.81608 -0.79402 1.20621

O -5.33458 0.09919 0.16474

O -6.06008 -2.16818 1.71272

H -6.65961 -1.41788 1.64998

O -5.05564 -4.64167 0.70315

H -5.68742 -4.60505 1.42904

O -2.45352 -4.49687 -0.37010

H -2.91979 -5.31041 -0.14675

C -1.75235 -2.09660 -1.86813

H -1.43781 -1.06592 -2.03863

H -2.28342 -2.44499 -2.76157

O -0.58071 -2.85683 -1.63501

H -0.84364 -3.73858 -1.34090

C -4.80903 1.37135 0.25002

C -3.88498 3.98584 0.44599

C -5.72774 2.40866 0.39263

C -3.45024 1.62348 0.20699

C -2.98026 2.94275 0.27508

C -5.25108 3.71076 0.49805

H -6.78791 2.18513 0.43194

H -2.74438 0.81568 0.10169

H -3.55599 5.01616 0.50311

O -6.08857 4.77330 0.65054

H -7.00163 4.47483 0.64089

C -1.53226 3.18309 0.13401

H -1.11193 4.03494 0.66128

C -0.77330 2.37190 -0.61483

H -1.29088 1.58800 -1.16270

C 0.67695 2.32239 -0.79371

C 3.41033 1.96627 -1.04434

C 1.56915 3.23968 -0.21024

C 1.17423 1.24192 -1.52733

C 2.54320 1.06902 -1.63360

C 2.94701 3.07881 -0.33110

H 1.17306 4.07324 0.35502

H 0.50422 0.53092 -1.99257

O 3.88845 3.88903 0.21234

C 3.43504 5.02351 0.91950

H 2.83880 4.73448 1.79135

H 2.84147 5.67898 0.27453

H 4.32802 5.54937 1.24907

C 3.36750 -0.01378 -2.28244

H 3.41965 0.16331 -3.36240

C 2.80243 -1.41629 -2.07799

H 2.61262 -1.58495 -1.01188

H 3.52873 -2.16721 -2.41300

O 1.60936 -1.50187 -2.83322

H 1.01196 -2.14796 -2.43165

C 4.76461 0.26051 -1.64100

C 5.08737 -0.65044 -0.47816

C 5.54334 -2.42966 1.62656

C 5.78425 -1.83149 -0.68948

C 4.62628 -0.34439 0.80718

C 4.85044 -1.22826 1.84827

C 6.01135 -2.72286 0.35925

H 6.15955 -2.06690 -1.67986

H 4.10778 0.58905 0.98059

H 6.55843 -3.64543 0.21018

O 4.45235 -1.04488 3.14193

C 3.72865 0.13526 3.43748

H 4.33235 1.02339 3.22996

H 3.49483 0.09042 4.49850

H 2.80290 0.17902 2.85612

O 5.76218 -3.28744 2.65400

H 5.37441 -2.90303 3.44928

H 5.56110 0.20113 -2.38249

O 4.71971 1.63496 -1.18659
